# Supplementary figures and images for: Spatial variability of soil chemical properties under different land-uses in Northwest Ethiopia
Source: PLoS One. 2021 Jun 23;16(6):e0253156. doi: 10.1371/journal.pone.0253156 (PMC8241222; doi:10.1371/journal.pone.0253156)

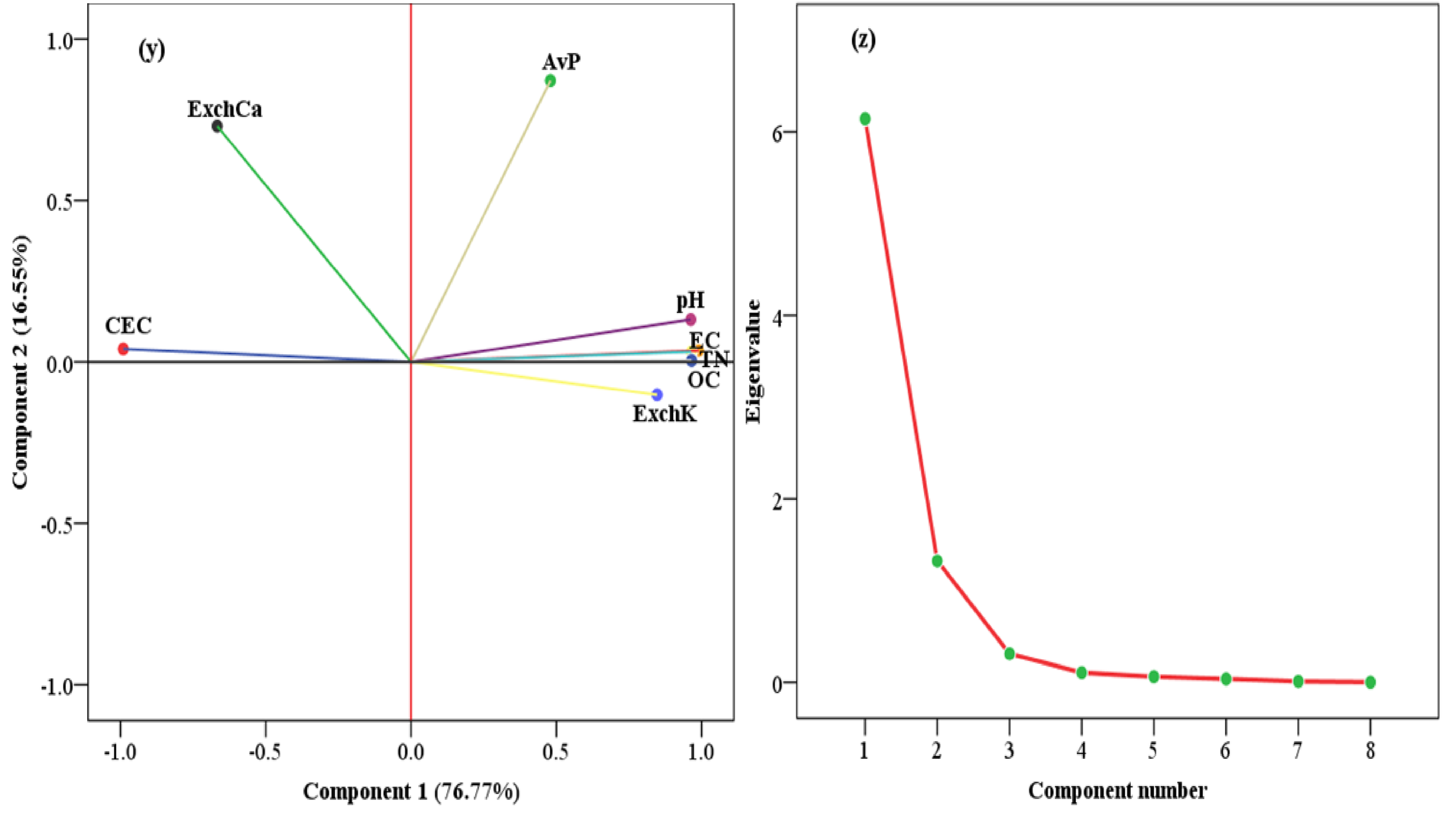

Supplement: S1 Fig — Organic carbon (OC), Total nitrogen (TN) Available phosphorus (avP), Exchangeable calcium (ExchCa), Exchangeable potassium (ExchK), Cation exchange capacity (CEC), and Electrical conductivity (EC). (TIF) [file pone.0253156.s001.tif]

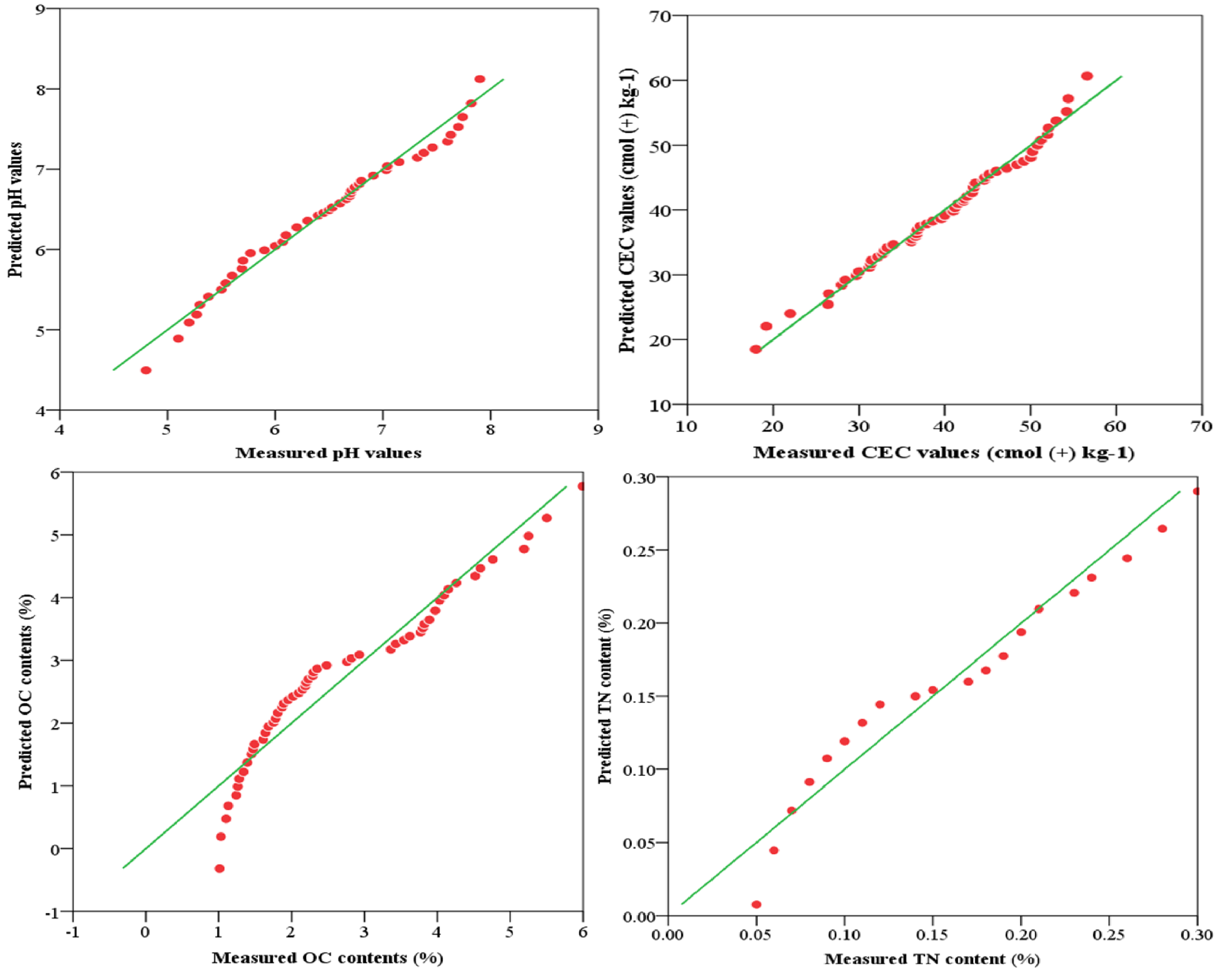

Supplement: S2 Fig — pH, Cation exchange capacity (CEC), Organic carbon (OC), and Total nitrogen (TN). (TIF) [file pone.0253156.s002.tif]
