# Supplementary material for: Spatial variability of soil chemical properties under different land-uses in Northwest Ethiopia
Source: PLoS One. 2021 Jun 23;16(6):e0253156. doi: 10.1371/journal.pone.0253156 (PMC8241222; doi:10.1371/journal.pone.0253156)
Supplement: Attachment — Submitted filename: PONE-D-21-03433_reviewer.pdf [file pone.0253156.s005.pdf]

PLOS ONE

Spatial variability of soil properties under different land-uses in Northwest Ethiopia

--Manuscript Draft--

**Manuscript Number:**	PONE-D-21-03433
**Article Type:**	Research Article
**Full Title:**	Spatial variability of soil properties under different land-uses in Northwest Ethiopia
**Short Title:**	Spatial variability of soil properties under different land-uses in Northwest Ethiopia
**Corresponding Author:**	Gizachew Ayalew Tiruneh, MSc, PhD candidate<br>Bahir Dar University College of Agriculture and Environmental Sciences<br>Bahir Dar, ETHIOPIA
**Keywords:**	land-use; soil properties; spatial variability
**Abstract:**	Inverse Distance Weighting (IDW), pair-wise comparisons, and descriptive analysis were used to evaluate the spatial variability of soil chemical properties under different land-uses of Fogera plain, northwest Ethiopia. We collected soil samples from three representative land-uses (cropland, plantation forestland, and grazing land) at topsoil using a grid-sampling design during the dry season of 2019. The analysis of variance for soil pH, available phosphorus (av. P), organic carbon (OC), total nitrogen (TN), electrical conductivity (EC), exchangeable Ca, and K, and cation exchange capacity (CEC) showed significant variations among the land-uses. The highest mean values of pH (8.44), av. P (31.51 ppm), OC (3.49 %), TN (0.313 %), EC (2.22 dS m −1), and exch. K (3.6 cmol(+) kg-1) were determined under grazing land. The lowest pH (6.58), OC (2.44 %), TN (0.17 %), exch. K (0.87 cmol(+) kg -1), and EC (0.13 dS m −1) were recorded in cultivated land. None of the soil variables showed high variability (coefficient of variation ≥35 %). As a change from grassland to cultivated land caused a substantial decline in soil organic carbon and total nitrogen, it is recommended to replenish soil nutrient content and maintain agricultural productivity within the Fogera plain.
**Order of Authors:**	Gizachew Ayalew Tiruneh, MSc, PhD candidate<br>Tiringo Yilak Alemayehu<br>Derege Tsegaye Meshesha<br>Eduardo Saldanha Vogelmann<br>José Miguel Reichert<br>Nigussie Haregeweyn
**Additional Information:**	
**Question**	**Response**
**Financial Disclosure**<br><br>Enter a financial disclosure statement that describes the sources of funding for the work included in this submission. Review the submission guidelines for detailed requirements. View published research articles from *PLOS ONE* for specific examples.<br><br>This statement is required for submission and **will appear in the published article** if the submission is accepted. Please make sure it is accurate.	The first author is a Ph.D. candidate in soil science at Bahir Dar University, Ethiopia. Our grateful acknowledgment directs to Debre Tabor University and Bahir Dar University for the financial grant. The Amhara Regional Soil Laboratory Center was substantially recognized for laboratory analysis for this study. Water and Land Resource Centre (WLRC) and Ethiopian Mapping Agency (EMA) are also acknowledged for various data access. The funders had no role in study design, data collection and analysis, decision to publish, or preparation of the manuscript.

Powered by Editorial Manager® and ProduXion Manager® from Aries Systems Corporation

**Unfunded studies**<br>Enter: *The author(s) received no specific funding for this work.*<br><br>**Funded studies**<br>Enter a statement with the following details:<br>• Initials of the authors who received each award<br>• Grant numbers awarded to each author<br>• The full name of each funder<br>• URL of each funder website<br>• Did the sponsors or funders play any role in the study design, data collection and analysis, decision to publish, or preparation of the manuscript?<br>• **NO** - Include this sentence at the end of your statement: *The funders had no role in study design, data collection and analysis, decision to publish, or preparation of the manuscript.*<br>• **YES** - Specify the role(s) played.<br><br>* typeset	
**Competing Interests**<br><br>Use the instructions below to enter a competing interest statement for this submission. On behalf of all authors, disclose any competing interests that could be perceived to bias this work—acknowledging all financial support and any other relevant financial or non-financial competing interests.<br><br>This statement is required for submission and **will appear in the published article** if the submission is accepted. Please make sure it is accurate and that any funding sources listed in your Funding Information later in the submission form are also declared in your Financial Disclosure statement.<br><br>View published research articles from *PLOS ONE* for specific examples.	The authors have declared that no competing interests exist.

Powered by Editorial Manager® and ProduXion Manager® from Aries Systems Corporation

**NO authors have competing interests**<br><br>Enter: *The authors have declared that no competing interests exist.*<br><br>**Authors with competing interests**<br><br>Enter competing interest details beginning with this statement:<br><br>*I have read the journal's policy and the authors of this manuscript have the following competing interests: [insert competing interests here]*<br><br>* typeset	
**Ethics Statement**<br><br>Enter an ethics statement for this submission. This statement is required if the study involved:<br><br>• Human participants<br>• Human specimens or tissue<br>• Vertebrate animals or cephalopods<br>• Vertebrate embryos or tissues<br>• Field research<br><br>Write "N/A" if the submission does not require an ethics statement.<br><br>General guidance is provided below. Consult the submission guidelines for detailed instructions. **Make sure that all information entered here is included in the Methods section of the manuscript.**	The Research and Publication Directorates of Debre Tabor University and Bahir Dar University approved the present study to collect soil samples and access the field site. As the present research has no negative impacts on a human, farmers allowed us to collect the soil samples in the study region.

Powered by Editorial Manager® and ProduXion Manager® from Aries Systems Corporation

**Format for specific study types**<br><br>**Human Subject Research (involving human participants and/or tissue)**<br>• Give the name of the institutional review board or ethics committee that approved the study<br>• Include the approval number and/or a statement indicating approval of this research<br>• Indicate the form of consent obtained (written/oral) or the reason that consent was not obtained (e.g. the data were analyzed anonymously)<br><br>**Animal Research (involving vertebrate animals, embryos or tissues)**<br>• Provide the name of the Institutional Animal Care and Use Committee (IACUC) or other relevant ethics board that reviewed the study protocol, and indicate whether they approved this research or granted a formal waiver of ethical approval<br>• Include an approval number if one was obtained<br>• If the study involved *non-human primates*, add *additional details* about animal welfare and steps taken to ameliorate suffering<br>• If anesthesia, euthanasia, or any kind of animal sacrifice is part of the study, include briefly which substances and/or methods were applied<br><br>**Field Research**<br><br>Include the following details if this study involves the collection of plant, animal, or other materials from a natural setting:<br>• Field permit number<br>• Name of the institution or relevant body that granted permission	
**Data Availability**<br><br>Authors are required to make all data underlying the findings described fully available, without restriction, and from the time of publication. PLOS allows rare exceptions to address legal and ethical concerns. See the PLOS Data Policy and FAQ for detailed information.	Yes - all data are fully available without restriction

A Data Availability Statement describing where the data can be found is required at submission. Your answers to this question constitute the Data Availability Statement and **will be published in the article**, if accepted.<br><br>**Important:** Stating 'data available on request from the author' is not sufficient. If your data are only available upon request, select 'No' for the first question and explain your exceptional situation in the text box.<br><br>Do the authors confirm that all data underlying the findings described in their manuscript are fully available without restriction?	
**Describe where the data may be found in full sentences. If you are copying our sample text, replace any instances of XXX with the appropriate details.**<br><br>• If the data are **held or will be held in a public repository**, include URLs, accession numbers or DOIs. If this information will only be available after acceptance, indicate this by ticking the box below. For example: *All XXX files are available from the XXX database (accession number(s) XXX, XXX.).*<br>• If the data are all contained **within the manuscript and/or Supporting Information files**, enter the following: *All relevant data are within the manuscript and its Supporting Information files.*<br>• If neither of these applies but you are able to provide **details of access elsewhere**, with or without limitations, please do so. For example:<br><br>*Data cannot be shared publicly because of [XXX]. Data are available from the XXX Institutional Data Access / Ethics Committee (contact via XXX) for researchers who meet the criteria for access to confidential data.*<br><br>*The data underlying the results presented in the study are available from (include the name of the third party*	All relevant data are within the manuscript and its Supporting Information files.

and contact information or URL).<br>• This text is appropriate if the data are owned by a third party and authors do not have permission to share the data.<br><br>* typeset	
Additional data availability information:	Tick here if the URLs/accession numbers/DOIs will be available only after acceptance of the manuscript for publication so that we can ensure their inclusion before publication.

Manuscript

Click here to access/download;Manuscript;Ethiopia_PLOS ONE_Variability of soils_2020.docx

Spatial variability of soil properties under different land-uses in Northwest Ethiopia

Gizachew Ayalew[1,#a¶], Tiringo Yilak[2¶], Derege Tsegaye Meshesha[3¶], Eduardo Saldanha, Vogelmann[4¶], José Miguel Reichert[5¶], Nigussie Haregeweyn[6¶]

[1] Department of Natural Resources Management, Faculty of Agriculture and Environmental Sciences, Debre Tabor University, P. O. Box 272, Debre Tabor, Ethiopia

[2] Department of Plant Sciences, Faculty of Agriculture and Environmental Sciences, Debre Tabor University, P. O. Box 272, Debre Tabor, Ethiopia

[3] Geospatial Data and Technology Center, College of Agriculture and Environmental Sciences, Bahir Dar University, P. O. Box 5501, Bahir Dar, Ethiopia

[4] Biological Sciences Institute, Federal University of Rio Grande, São Lourenço do Sul, Brazil

[5] Soils Department, Federal University of Santa Maria (UFSM), Av. Roraima 1000, 97105-900 Santa Maria, RS, Brazil

[6] International Platform for Dryland Research and Education, Tottori University, Tottori, Japan

[#a] Current Address: Department of Natural Resources Management, College of Agriculture and Environmental Sciences, Bahir Dar University, P. O. Box 5501, Bahir Dar, Ethiopia

* Corresponding author: E-mail: tiruneh1972@gmail.com

Abstract

Inverse Distance Weighting (IDW), pair-wise comparisons, and descriptive analysis were used to evaluate the spatial variability of soil chemical properties under different land-uses of Fogera plain in the northwest region in Ethiopia. Soil samples were collected from three representative land-uses (cropland, plantation forestland, and grazing land) at topsoil using a grid-sampling design during the dry season of 2019. The analysis of variance for soil pH, available phosphorus (av. P), organic carbon (OC), total nitrogen (TN), electrical conductivity (EC), exchangeable Ca, and K, and cation exchange capacity (CEC) showed significant variations among the land-uses. The highest mean values of pH (8.44), av. P (31.51 ppm), OC (3.49 %), TN (0.313 %), EC (2.22 dS m^{-1}), and exch. K (3.6 cmol(+) kg^{-1}) were determined under grazing land. The lowest pH (6.58), OC (2.44 %), TN (0.17 %), exch. K (0.87 cmol(+) kg^{-1}), and EC (0.13 dS m^{-1}) were recorded in cultivated land. None of the soil variables showed high variability (coefficient of variation ≥35 %). As a change from grassland to cultivated land caused a substantial decline in soil organic carbon and total nitrogen, it is recommended to replenish soil nutrient content and maintain agricultural productivity within the Fogera plain.

Keywords: land-use; soil properties; spatial variability

Introduction

Environmental degradation caused by inappropriate land use is a worldwide problem in sustainable agriculture. Land-use change markedly affects soil properties [1, 2]. Changing land-use from forest cover to plough-land could create a decline in soil fertility, nutrients, and thus productivity [3-6], and increased soil perturbation [5, 7-12].

In Ethiopia, rapid population growth and environmental factors lead to the conversion of forestland and grassland into cultivated land [13]. [14] examined that the expansion of

cultivated areas can substantially affect soil nutrient content. [15] reported changes in the amount of soil organic carbon, and total N due to changes in land-use and land-cover in the Gerado catchment, northeastern Ethiopia. [16] also investigated that deforestation has resulted in deterioration of soil organic matter in the soil. Thus, soil nutrient depletion is a serious problem and a major crop production constraint in the country [17-18]. Thus, scientific information of spatial variability and distribution of soil properties among land-use shifts are critical to optimize fertilizer use and boost crop productivity [19].

Ethiopia has been showing an increase in cultivated lands and eucalyptus plantations and a decrease in grazing lands due to the population growth [13]. The eucalyptus plantation considerably affected soil properties [20-22]. [23] stated a decrease in soil organic carbon (OC), total nitrogen (TN), exchangeable cations, and cation exchange capacity (CEC) contents owing to the shift of woodlands to croplands and grazing lands in the same country. [24] also found declining pH value and the soil organic matter content in cultivated land in the Kabe watershed, Ethiopia.

Recently, many scholars have been using geostatistics for estimating the spatial variability of soil properties [25-28]. Knowledge of the spatial variability of soil properties [29] and developing site-specific recommendations [29-30] are vital for optimizing nutrient use, improving crop productivity, and minimizing environmental hazards [31].

However, this information is scarce and there are few studies on the state of the art and assessments on the situation of soil degradation in Ethiopia. The spatial information generated using geostatistical techniques about soil properties, soil fertility status, and nutrient management to enhance food security and obtain sustainable yield in developing countries including Ethiopia have never been well-studied using spatial prediction models [32]. Knowledge of the soil fertility of various land-use types that could be used for predicting, monitoring, and evaluating the effects of changes in land use types on soil

properties, scheming appropriate land-use planning, and sustaining the productivity of agriculture in the Fogera plain area has also not been yet adequately addressed.

Thus, the objective of this research paper was to evaluate the impacts of land-use changes on spatial variability and distribution of soil fertility qualities, including pH, organic carbon (OC), total nitrogen (TN), available phosphorus (av. P), calcium (Ca^{2+}), potassium (K^{+}), electrical conductivity (EC), and cation exchange capacity (CEC) in Fogera plain, the highland of Ethiopia.

Materials and methods

Description of the study area

The study was performed within the Fogera plain area (37° 25' 15" E - 37° 59' 20" E and 11° 16' 53" N-12° 21' 51" N) in the northwest highland of Amhara region, Ethiopia (Fig 1). It covers an area of 5,646 hectares. The main crops cultivated in the study area are rice (*Oryza sativa* L.), maize (*Zea mays* L.), and Teff [*Eragrostis tef* (Zucc.)]. The important income sources for the local people are rice, onion, and eucalyptus products.

Spatial data sources and their extraction

Land-use/land-covers of the study area for 2019 were deduced from a 1:20,000 scale land-use/land-cover map made by the Ethiopia Mapping Agency (EMA), and also the major land-use system consisted of cropland (4,362.73 ha, 77.27 %), plantation forestland (6,96.29 ha, 12.33 %) and grassland (586.98 ha, 10.39 %). We also performed reconnaissance surveys in August–November/2019 to verify the map. A digital elevation model (ASTER DEM) with a 20 * 20 m resolution, downloaded from the EMA website [33] was employed to generate elevation and slope of the study area using ArcGIS software version 10.5. The agroecology belonging to Kolla (<1800 m a.s.l.), Weyna dega (1,800–2,400 m a.s.l.) and dega (>2400 m a.s.l.) has area share of 16.24 %, 71.93 % and 11.83 %, respectively (Fig 2). The digital soil

map collected from Water and Land Resource Centre (WLRC) shows that the soil types of the Fogera plain area are Chromic Vertisols (48.57 %), Eutric Nitosols (17.65 %), Orthic Luvisols (15.75 %), Eutric Cambisols (10.78 %), Chromic Luvisols (3.97 %), and Lithosols (3.27 %) [34].

Soil sampling, laboratory analysis, and statistical analysis

The fishnet tool of the ArcGIS software version 10.5 was used to generate a regularly spaced grid of sampling points on the land-uses in the study region. Next, 60 representative soil samples (20 from each use: cropland, plantation forestland, and grazing land) at a depth of 0-20 cm were recognized through a systematic purposive approach in February-April/2020. A portable Global Positioning System (Garmin 60; 2 m accuracy) was used to locate each grid center in the field and to record latitudes, longitudes, and elevations of sampling points. A kilogram of composite soil sample per site was collected, air-dried, grinded using mortar and pestle, and analyzed at the Amhara Regional Soil Laboratory Center.

Soil pH was measured potentiometrically in H_2O at the soil to solution ratio of 1: 2.5 using a combined glass electrode pH meter (Model CP-505, Zabrze ul, Poland) [35]. Electrical conductivity (EC) of the soil was measured at the soil to water ratio of 1: 5 using an EC meter (Orion Model 145, USA) [36]. Soil organic carbon (OC) was determined following the Walkley-Black procedures. A weighed amount (20-50 mg) of the dried, ground soil samples was treated with 5 ml of 0.4 N potassium dichromate solution ($K_2Cr_2O_7$) followed by the addition of 10 ml of concentrated sulfuric acid. The mixture was gently mixed and left for 16-18 hours and then, 100 ml of triple-distilled water was added to the mixture. The excess of dichromate was back-titrated potentiometrically with the standard 0.2 N ferrous ammonium sulfate solution. Blank titration of the acidic dichromate with ferrous ammonium sulfate solution was performed [37].

Total nitrogen (TN) of the soils was determined through digestion, distillation, and titration procedures of the Kjeldahl using the Kjeldahl apparatus (Gallenhamp, USA) [38]. Available phosphorus (av. P) in the soil was determined using the 0.5 M $NaHCO_3$, pH of 8.5, in 1: 20 of soil to solution ratio for half an hour. The av. P was extracted with 1 M ammonium chloride, 0.5 M ammonium fluoride, 0.1 M sodium hydro-oxide (from Blulux Laboratory Reagent (p) Ltd), and the amount was measured by a spectrophotometer (UV1700, Japan) [39-40]. Exchangeable calcium (Ca), magnesium (Mg), potassium (K), and sodium (Na) were determined by saturating the soil samples with 1 M ammonium acetate solution at pH 7.0. Subsequently, Ca and Mg were determined by using Perkin-Elmer Model 290 atomic absorption spectrophotometer (ColVisTec, Germany); while exchangeable Na and K were measured by a Model 18 Perkin-Elmer flame photometer [41].

The cation exchange capacity (CEC) of the soil was determined from the NH_4^+ saturated samples that were replaced by K^+ from a percolated KCl solution (from LOBA CHEMIE PVT.LTD). The excess salt was removed by washing with ethanol (from LOBA CHEMIE PVT.LTD), and the NH_4^+ was displaced by K^+ [42]. All the chemicals and reagents, including potassium dichromate, sulphuric acid, ferrous sulfate, ferroin, sodium bicarbonate, ammonium chloride, ammonium fluoride, and ammonium acetate were supplied by Merck KGaA and Sigma-Aldrich, Steinheim, Germany, unless otherwise specified.

Inverse Distance Weighting (IDW) has been widely used for the prediction of soil OM and soil nitrate [43], P and K levels [44], and soil pH scale [45], the current study employed IDW for mapping spatial distribution of the soil chemical properties under the ArcGIS environment. Besides, pair-wise comparisons and descriptive analysis were carried out using Statistical Analyses System (SAS) software version 9.4 and Statistical Package for Social Sciences (SPSS) software version 20, respectively. Means were compared using the Tukey test at 1% probability.

Ethics statement

The Research and Publication Directorates of Debre Tabor University and Bahir Dar University approved the present study to collect soil samples and access the field site. As the present research has no negative impacts on a human, farmers allowed us to collect the soil samples in the study region.

Results and discussion

Effect of land-use/land-cover types on soil fertility quality

Soil pH_{H2O}

Based on the scores set by [46], a substantial part of the soils of e*ucalyptus* plantation forestland (79.87 %), grazing land (68.90 %), and cropland (59.69 %) was moderately alkaline (7.4-8.4) (Table 1 and Fig 4). Higher soil pH levels obtained from plantation forestland and grazing land might be because of the existence of basic cations emanated from weathering [24], and the potash obtained from ashes [47].

Table 1. Soil pH_{H2O} rating, its area share (ha, %) by land-use, and its parametric test.

Common name	pH rating	Area share (ha, %) by land-use					
		Cropland		Plantation forestland		Grazing land	
		Area (ha)	%	Area (ha)	%	Area (ha)	%
Moderately acid	5.6-6.5	181.84	4.17	1.11	0.16	9.50	1.62
Neutral	6.6-7.3	1,576.93	36.15	139.05	19.97	173.04	29.48
Moderately alkaline	7.4-8.4	2,603.96	59.69	556.12	79.87	404.44	68.90
Total		4,362.73	100	696.29	100	586.98	100
Mean pH		6.58^{a}		7.40^{b}		8.44^{c}	

Means of pH with different letters are significantly different (Tukey, $p < 0.01$).

Available phosphorus (av. P)

A notable variation in soil av. P was obtained to land-uses. [48] rated very high (>20 ppm) av. P content in soils of all land-uses (Table 2 and Figs 3-4). Higher av. P values in the soils might be revealed by the recurrent use of mineralized phosphorus [31], and the addition of manure, compost, and ashes [49], presence of weathered soil minerals [50], and actions of microbes. Higher levels of av. P in the soils indicate that the soils have optimum the nutrient for crop growth. [23, 51] made related reports in Ethiopia.

Table 2. Soil available phosphorus (av. P) rating and its area share (ha, %) by land-use and its parametric test.

Common name	Av. P rate	Area share (%) by land-use					
		Cropland		Plantation forestland		Grazing land	
	ppm	Area (ha)	Area (%)	Area (ha)	Area (%)	Area (ha)	Area (%)
Medium	11-14	273.66	6.27	35.02	5.03	23.05	3.93
High	15-20	1,425.74	32.68	200.90	28.85	118.12	20.12
Very high	>20	2,663.33	61.05	460.37	66.12	445.82	75.95
Total		4,362.73	100	696.29	100	586.98	100
Mean av. P (ppm)		21.32^{a}		11.43^{b}		32.52^{c}	

Means of available P with different letters are significantly different (Tukey, $p < 0.01$).

Soil organic carbon (OC) and total nitrogen (TN)

A significant variation in soil organic carbon (OC) and total nitrogen (TN) was recorded from land-uses. As per the rating set by [52], low organic carbon content (2.44 %) in the soils dominated the cultivated land (87.66 %), grazing land (81.7 %), and eucalyptus plantation forestland (80.63 %) (Table 3 and Figs 3-4). This could occur due to the high

erosion rate, crop residue exclusion, increased rate of mineralization, and the deficiency of nutrients [31, 53]. The higher organic carbon and available P contents of the grazing lands indicate that OM is the principal supply of av. P [54].

Table 3. Soil organic carbon (OC) rating and its area share (ha, %) by land-use and its parametric test.

Common name	OC rate	Area share (ha, %) by land-use					
		Cropland		Plantation forestland		Grazing land	
	%	Area (ha)	Area (%)	Area (ha)	Area (%)	Area (ha)	Area (%)
Low	2-4	3,824.5	87.66	561.44	80.63	479.59	81.70
Medium	4-8	538.23	12.34	134.85	19.37	107.40	18.30
Total		4,362.73	100	696.29	100	586.98	100
Mean OC (%)		2.44^{a}		3.43^{b}		4.35^{c}	

Means of soil OC with different letters are significantly different (Tukey, $p < 0.01$).

According to the rate of [48], the cropland (16.29 %) and plantation forestland (69.14 %) dominantly exhibited, respectively, low (0.17 %) and high total nitrogen (0.35 %) contents in the soils (Table 4 and Figs 3-4). In line with this, lower soil OC content was obtained in the croplands [2, 24, 55]. Higher soil OC and TN contents recorded at plantation forests are probably attributed to the burning of grass and deposition of dung in grazing lands. Besides, researchers have encouraged grazing to maintain nutrient cycling and decomposition rates [56]. The low total nitrogen contents might be qualified to the loss of nitrogen through volatilization or leaching and rapid decomposition of OM. Therefore, the shift of grassland and eucalyptus plantation to cultivated land exacerbates soil OC and TN decline. Thus, the soils call for external nitrogen and carbon inputs for sustainable production.

Table 4. Soil total nitrogen (TN) rating and its area share (ha, %) by land-use and its parametric test.

Common name	TN rate	Area share (ha, %) by land-use					
		Cropland		Plantation forestland		Grazing land	
	%	Area (ha)	Area (%)	Area (ha)	Area (%)	Area (ha)	Area (%)
Low	0.1-0.2	1,219.37	27.95	69.66	16.29	115.14	19.62
Medium	0.2-0.3	2,159.42	49.50	62.31	14.57	244.65	41.68
high	0.3-0.4	983.94	22.55	295.63	69.14	227.18	38.70
Total		4,362.73	100	427.60	100	586.98	100
Mean TN (%)		0.17^a		0.27^b		0.35^c	

Means of TN with different letters are significantly different (Tukey, $p < 0.01$).

Exchangeable cations (K, Ca) and electrical conductivity (EC)

As shown in Tables 5-6 and Fig 3, a significant variation of the contents of soil K, Ca, and EC was observed from land-uses. Besides, the soils of the study area consisted of high contents of Ca (>20 $cmol_{(+)}\ kg^{-1}$) and K (0.51-1.51 $cmol_{(+)}\ kg^{-1}$) as per the rates by [46, 48]. Higher Ca and K levels might be attributable to the type of parent materials, weathering, land-use types, forms of fertilizer used and their leaching rate, crop remains, and litterfall. The higher contents of Ca and K in both grazing and plantation forestlands could be linked to its higher pH value [57] and clay particles [58] were stated. On the other hand, the soils showed slightly salty. This result suggests that Ca and K do not seem to be limiting nutrients to crop production in the area.

Table 5. Soil exchangeable potassium (exch. K) rating and its area share (ha, %) by land-use and its parametric test

Common name	Exch. K Rate	Area share (ha, %) by land-use					
		Cropland		Plantation forestland		Grazing land	
	$cmol_{(+)}\ kg^{-1}$	Area (ha)	Area (%)	Area (%)	Area (ha)	Area (%)	Area (ha)
High	0.51-1.51	2,609.17	59.81	320.17	45.98	257.26	43.83
Medium	1.51-2.3	1,733.37	39.73	373.31	53.61	326.57	55.64
Very high	>2.3	20.19	0.46	2.80	0.40	3.15	0.54
Total		4,362.73	100	696.29	100	586.98	100
Mean exch. K ($cmol_{(+)}\ kg^{-1}$)		0.87^{a}		1.47^{b}		1.81^{c}	

Means of exch. K with different letters are significantly different (Tukey, $p < 0.01$).

Table 6. Electrical conductivity (EC) rating and its area share (ha, %) by land-use and its parametric test.

Common name	EC Rate	Area share (ha, %) by land-use					
		Cropland		Plantation forestland		Grazing land	
	$dS\ m^{-1}$	Area (ha)	Area (%)	Area (ha)	Area (%)	Area (ha)	Area (%)
Not salty	<0.75	1,427.14	32.71	96.07	13.80	146.18	24.90
Slightly salty	0.75-2	2,660.19	60.98	538.15	77.29	372.90	63.53
Moderately salty	2-4	275.30	6.31	62.07	8.91	67.90	11.57
Sum		4,362.63	100	696.29	100	586.98	100
Mean EC ($dS\ m^{-1}$)		0.13^{a}		1.21^{b}		2.22^{c}	

Means of EC with different letters are significantly different (Tukey, $p < 0.01$).

Cation Exchange Capacity (CEC)

As shown in Table 7 and the rate set by [48], a significant variation and higher CEC indicated soils of the study area have a high potential to hold nutrients against leaching losses. The highest CEC values recorded in cultivated land-use might be caused by soil organic material, pH, quantity, and kind of clay, which adsorb and hold positive cations through electrostatic force [59]. The current findings were also in agreement with [60-62] reported higher CEC under cultivated lands in the highlands of Ethiopia.

Table 7. Cation exchange capacity (CEC) rating and its area share (ha, %) by land-use and its parametric test.

Common name	CEC rate	Area share (ha, %) by land-use					
		Cultivated land		Plantation forestland		Grazing land	
	$cmol_{(+)}$ kg^{-1}	Area (ha)	Area (%)	Area (ha)	Area (%)	Area (ha)	Area (%)
High	15-30	1,402.31	32.14	357.07	51.28	275.72	46.97
Very high	>30	2,960.42	67.86	339.22	48.72	311.26	53.03
Sum		4,362.73	100	696.29	100	586.98	100
Mean CEC		40.36^{a}		31.53^{b}		25.24^{c}	

Means of CEC with different letters are significantly different (Tukey, $p < 0.01$).

Descriptive statistics of soil fertility parameters

Table 8 shows the highest (8.44) and the lowest (6.58) soil pH values were observed under the grazing and the cultivated lands, respectively. The highest (4.35 %) and lowest (2.44 %) OC contents obtained in the grazing and croplands demonstrated that soil OC showed a strong response to land-use type [63]. Following the limit set by [46], high av. P levels (>20 ppm) and very high CEC (>20 $cmol_{(+)}$ kg^{-1}) were observed in all land-uses. Based

on the EC rate established by [64], no significant amount of soluble salts was accumulated, thus will not affect plant growth and development.

Table 8. Descriptive information soil chemical properties within the study area

Statistic	LULC	Soil fertility parameters							
		pH_{H2O}	OC	TN	Av. P	Exch. Ca	Exch. K	CEC	EC
			%	%	ppm	$cmol_{(+)}\ kg^{-1}$			$dS\ m^{-1}$
Standard deviation	CL	0.21	0.08	0.01	0.52	0.44	0.03	0.44	0.01
	FL	0.25	0.27	0.02	0.28	0.89	0.37	0.28	0.05
	GL	0.22	0.26	0.04	0.29	1.10	0.37	0.29	0.04
Coefficient of variation	CL	3.12	3.28	4.17	2.42	1.18	3.79	1.08	10.00
	FL	3.31	7.96	6.18	2.47	3.47	25.37	0.88	3.72
	GL	2.59	5.89	11.56	0.88	3.67	20.50	1.13	1.89
Kurtosis	CL	-0.67	0.34	0.08	1.78	0.86	2.28	-0.54	6.58
	FL	-0.31	-0.61	6.29	-1.27	0.35	-0.66	-0.97	-0.39
	GL	0.38	-0.53	2.23	-0.89	0.33	2.85	0.80	-0.59
Skewness	CL	0.14	0.08	0.00	-1.44	1.10	-0.73	0.36	2.14
	FL	-0.01	0.64	-2.27	0.12	0.32	-0.53	-0.29	-0.69
	GL	-0.14	0.63	-1.38	-0.15	-0.33	0.95	1.19	-0.53
Minimum	CL	6.00	2.00	0.15	20.01	37.00	1.00	40.00	0.00
	FL	7.00	3.00	0.21	11.01	24.00	1.00	31.00	1.00
	GL	8.00	4.00	0.23	32.04	27.00	1.00	25.00	2.00
Maximum	CL	7.00	3.00	0.18	21.94	39.00	1.00	41.00	0.00
	FL	8.00	4.00	0.28	11.92	28.00	2.00	32.00	1.00
	GL	9.00	5.00	0.39	32.99	32.00	3.00	26.00	2.00

OC, organic carbon; TN, total nitrogen; av. P, available phosphorus; Exch. Ca, exchangeable Calcium; Exch. K, exchangeable potassium; CEC, cation exchange capacity, EC, electrical conductivity; LULC, land-use/land-cover; CL, cropland; FL, forestland; GL, grazing land.

As indicated in Table 8, the skewness results, which varied from -2.27 to 2.14, showed almost all soil properties of the various land-uses were normally distributed. None of

the soil variables was categorized as high variability in terms of coefficient of variation (CV≥35 %). Based on the rate established by [65], soil pH, Ca, CEC, OC, TN, and av. P had low variability (CV≤15 %), which could be because of the area's high spatial homogeneity. Similar findings were also made in the country [15, 66]. Thus, the change of forestland to croplands aggravates the degradation of organic matter that affects most of the soil properties [67]. However, compost, cowl plants, manure, minimal tillage, and crop rotation could enhance organic matter content in soils [68]. Moderate variation (CV=15-34 %) in potassium at forestland and grazing land is possible because of the variations in soil management practices, parent material, and groundwater depth [19, 69].

Implications for sustainable soil fertility management and environmental conservation

Our results showed that the transition from grassland to cultivated land and eucalyptus plantation leads to a significant reduction in the total nitrogen within Fogera plain's topsoil. Nevertheless, av. P content of soils of the area was high (>20 ppm) which could be due to the repeated use of mineralized phosphorus [31]. The addition of nitrogen-containing fertilizer inputs may also improve the cultivated lands' soil nutrient supply to gain better crop yield. However, the overuse of nitrogen and phosphorus fertilizer can contribute to global climate change through their energy-intensive production and inefficient use [70]; eutrophication of water bodies [71]; and soil acidification [72]. Besides, there has been an increased total greenhouse gas emission including N_2O, CO_2 under cultivated lands [70].

The information of spatial soil variability across land-uses is vital for sustainable land-use planning which in turn could reduce soil erosion, enhance land productivity, and maintain soil and environmental quality [73, 74]. Moreover, the best land-use planning and policies should be developed to provide an optimal solution geared toward improving the soil

nutrient use efficiency and reducing the adverse environmental effects including nitrate losses to water and N_2O emissions [75-76].

Conclusion

A substantial variation of soil pH, available phosphorus (av. P), organic carbon (OC), total nitrogen (TN), electrical conductivity (EC), exchangeable bases (Ca and K), and cation exchange capacity (CEC) was obtained across land-uses. The highest mean values of pH (8.44), av. P (31.51 ppm), OC (3.487 %), TN (0.313 %), EC (2.22 dS m^{-1}), and exch. K (3.6 $cmol_{(+)}$ kg^{-1}) were recorded under grazing land, while the lowest OC (2.44 %), TN (0.17 %), soil pH (6.58), exch. K (0.87 $cmol_{(+)}$ kg^{-1}), and EC (0.13 dS m^{-1}) were recorded in cultivated land. Most soil variables confirmed low spatial variability, which can be due to the area's high spatial homogeneity. The expansion of cultivated lands caused the depletion of soil OC and TN, limiting crop growth and declining crop yield. Thus, soil management alternatives should gear to replenish the OC and N contents of the soil for increasing crop yield in the area. Besides, further studies should be double-geared to examine the effects of land-use type on long-term spatial patterns of soil properties of the Fogera plain.

Acknowledgment

Our grateful acknowledgment directs to Debre Tabor University and Bahir Dar University for the financial grant. The Amhara Regional Soil Laboratory Center was substantially recognized for laboratory analysis for this study. Water and Land Resource Centre (WLRC) and Ethiopian Mapping Agency (EMA) are also acknowledged for various data access.

Author contributions

Gizachew Ayalew designed the method and wrote the draft manuscript. Tiringo Yilak and Derege Tsegaye Meshesha have been accountable for data analysis, and interpretation of

the results. Eduardo Saldanha Vogelmann, José Miguel Reichert, and Nigussie Haregeweyn analyzed/interpreted the results, reviewed/commented on the manuscript.

Declaration of competing interests

The authors declare that they have no competing interests.

References

1. Burst M, Chauchard S, Dambrine E, Dupouey JL, Amiaud B. Distribution of soil properties along with forest-grassland interfaces: influence of permanent environmental factors or land-use after-effects. Agric Ecosyst Environ. 2020; 289:106739. https://doi.org /10.1016/j.agee.2019.106 739.
2. Fentie SF, Jembere K, Fekadu E, Wasie D. Land-use and land-cover dynamics and properties of soils under different land-uses in the tejibara watershed, Ethiopia. Sci World JP. 2020; 1-12. https://doi.org/10.1155/2020/1479460.
3. Saha D, Kukal SS. Soil Structural Stability and Water Retention Characteristics under Different Land-uses of Degraded Lower Himalayas of North-West India. Land Degrad Dev. 2013; 26(3): 263–271. doi:10.1002/ldr.2204.
4. Reichert JM, Rodrigues MF, Bervald CMP, Brunetto G, Kato OR, Schumacher MV. Fragmentation, fiber separation, decomposition, and nutrient release of secondary-forest biomass, mechanically chopped-and-mulched, and cassava production in the Amazon. Agric Ecosyst Environ. 2015; 204: 8–16. https://doi.org/10.1016/j.agee.2015.02.005.
5. Reichert JM, Rodrigues MF, Bervald CMP, Kato OR. Fire-free fallow management by mechanized chopping of biomass for sustainable agriculture in eastern Amazon: effects on soil compactness, porosity, and water retention and availability. Land Degrad Dev. 2016a; 27: 1403–1412. https://doi.org/10.1002/ldr.2395.

6. Bufebo B, Elias E. Effects of Land-Use/Land-Cover Changes on Selected Soil Physical and Chemical Properties in Shenkolla Watershed, South Central Ethiopia. Advances in Agriculture, 2020. https://doi.org/10.1155/2020/5145483.
7. Barua SK, Haque SMS. Soil characteristics and carbon sequestration potentials of vegetation in degraded hills of Chittagong, Bangladesh. Land Degrad Dev. 2013; 24: 63–71. https://doi.org/10.1002/ldr.1107.
8. Suzuki LEAS, Reichert JM, Reinert DJ, Lima CLR de. Degree of compactness and mechanical properties of a subtropical Alfisol with eucalyptus, native forest, and grazed pasture. Forest Sci. 2015; 61:716–722. https://doi.org/10.5849/forsci.14-172.
9. Reichert JM, Amado TJC, Reinert DJ, Rodrigues MF, Suzuki LEAS.. Land-use effects on subtropical, sandy soil under sandyzation/desertification processes. Agric Ecosyst Environ. 2016b; 233: 370–380. https://doi.org/10.1016/j.agee.2016.09.039.
10. Reichert JM, Cechin NF, Reinert DJ, Rodrigues MF, Suzuki LEAS. Ground-based harvesting operations of Pinus taeda affects the structure and pore functioning of clay and sandy clay soils. Geoderma. 2018; 331:38–49. https://doi.org/10.1016/j.geoderma.2018.06.012.
11. Holthusen D, Brandt AA, Reichert JM, Horn R. Soil porosity, permeability, and static and dynamic strength parameters under native forest/grassland compared to no-tillage cropping. Soil and Tillage Res. 2018a; 177:113–124. https://doi.org/10.1016/j.still.2017.12.003.
12. Holthusen D, Brandt AA, Reichert JM, Horn R, Fleige H, Zink A. Soil functions and in situ stress distribution in subtropical soils as affected by land-use, vehicle type, tire inflation pressure, and plant residue removal. Soil and Tillage Res. 2018b; 184:78–92. https://doi.org/10.1016/ j.still.2018.07.009.

13. Tesfahunegn GB, Tamene L, Vlek Paul LG. Catchment-scale spatial variability of soil properties and implications on site-specific soil management in northern Ethiopia. Soil Till Res. 2011; 117:124–139. doi.org/10.1016/j.still.2011.09.005.

14. Biro, K., Pradhan, B., Muchroithner, M., and Makeschin, F.: Land-use/land-cover change analysis and its impact on soil properties in the northern part of Gadarif region, Sudan, Land Degrad Dev. 2013; 24: 90–102. https://doi.org/10.1002/ldr.1116.

15. Asmamaw LB, Mohammed AA. Effects of slope gradient and land-use/land-cover on selected soil physical and biochemical properties of the Gerado catchment, North-eastern Ethiopia. Int J Environ Stud. 2013; 70: 111–125. https://doi.org/10.1080/00207233.2012.751167.

16. Emiru, N, Gebrekidan, H. Effect of land-use changes and soil depth on soil organic matter, total nitrogen and available phosphorus contents of soils in Senbat watershed, western Ethiopia. J Agric Biol Sci. 2013; 8: 206–212.

17. Stoorvogel JJ, Smaling EMA. Assessment of soil nutrient depletion in Sub-Saharan Africa: 1983-2000. Vol. 2: Nutrient balances per crop and land-use systems (Report/ Winand Staring Centre; No. 28). ISRIC, 1990. https://edepot.wur.nl/3 05176 19.

18. Stoorvogel JJ, Smaling EM, Janssen BH. Calculating soil nutrient balances in Africa at different scales. Fertilizer res. 1993; *35*(3): 227-235.

19. Tesfahunegn GB. Soil quality indicators response to land-use and soil management systems in Northern Ethiopia's catchment. Land Degrad Dev. 2016; 27: 438–448. https://doi.org/10.1002/ldr.2245, 2016.

20. Tadele D, Teketay D. Growth and yield of two-grain crops on sites former covered with eucalypt plantations in Koga Watershed, northwestern Ethiopia. J For Res. 2014; 25: 935–940. https://doi.org/10.1007/s11676-014-0483-9.

21. Jaleta D, Mbilinyi B, Mahoo H, Lemenih M. Eucalyptus expansion as relieving and provocative tree in Ethiopia. J Agric Ecol Res Int. 2016a; 6(3): 1-12. https://doi.org/10.9734/JAERI/2016/228 41.

22. Jaleta D, Mbilinyi B, Mahoo H, Lemenih M. Evaluation of land-use/land-cover changes and eucalyptus expansion in Meja watershed, Ethiopia. J Geo Environ Earth Sci Int. 2016b; 7(3): 1-12. https://doi.org/10.9734/JGEESI/2016/26606.

23. Fantaw Y, Abdu A. Soil property changes following the conversion of acacia woodland into grazing and farmlands in Ethiopia's rift valley area. Land Degrad Dev. 2011; 22:425–431. https://doi.org/10.1002/ldr.1022.

24. Assefa F, Elias E, Soromessa T, Ayele GT. Effect of changes in land-use management practices on soil physical and chemical properties in Kabe Watershed, Ethiopia. Air, Soil, and Water Res. 2020; 13: 1-16. https://doi.org/10.1177/1178622120939587.

25. Appel Neto E, Seidel EJ, Oliveira MS. Geostatistical-based index for spatial variability in soil properties. Rev Bras Cienc Solo. 2020; 44: e0200086. https://doi.org/10.36783/1806967r bcs20200086.

26. Ichami SM, Shepherd KD, Hoffland E, Karuku GN, Stoorvogel JJ. Soil spatial variation to guide the development of fertilizer use recommendations for smallholder farms in western Kenya. Geoderma Reg. 2020; p.e00300. https://doi.org/10.1016/j.geodrs.2020.e00300.

27. Jurišić M, Radočaj D, Krčmar S, Plaščak I, Gašparović M. Geostatistical Analysis of Soil C/N Deficiency and Its Effect on Agricultural Land Management of Major Crops in Eastern Croatia. Agronomy. 2020; 10(12): 1996. https://doi.org/10.3390/agronomy10121996.

28. Macedo Neto AAL, Farias PRS, Matos GSBD, Silva GBD, Santos AVFD, Anhê BB. Diagnosis and spatial variability of soil fertility and crop production in a teak area in

eastern Pará State Cerne. 2020; 26(1): 37-47.https://doi.org/10.1590/01047760202026012683.

29. Guan F, Xia M, Tang X, Fan S. Spatial variability of soil nitrogen, phosphorus, and potassium contents in Moso bamboo forests in Yong'an City, China. Catena, 2017; 150: 161–72. https://doi.org/10.1016/j.catena.201.11.017.
30. Rosemary F, Vitharana UWA, Indraratne SP, Weerasooriya R, Mishra U. Exploring the spatial variability of soil properties in an Alfisol soil catena. Catena, 2017; 150: 53–61. https://doi.org/10.1016/j.catena.2016.10.017.
31. Panday D, Maharjan B, Chalise D, Shrestha RK, Twanabasu B. Digital soil mapping in the Bara District of Nepal using Kriging tool in ArcGIS. PLoS ONE. 2018; 13(10):e0206350. https://doi. org/10.1371/journal.pone.0206350.
32. Behera SK, Shukla AK. Spatial distribution of surface soil acidity, electrical conductivity, soil organic carbon content, and exchangeable potassium, calcium, and magnesium in some cropped acid Soils of India. Land Degrad Dev. 2015; 26: 71–79. https://doi.org/10.1002/ldr.2306.
33. Ethiopian Mapping Agency, P.O.Box 597, Addis Abeba. [Accessed 2019 January 18]. http://www.ema.gov.et/.
34. Water and Land Research Center (WLRC). National Geospatial database System EthioGIS-2/Release. 2014.
35. Van Reeuwijk LP. Procedure for soil analysis. 4th ed. International soil reference center Wageningen. (ISRIC) Technical Paper No. 9, 1993. The Netherlands, Wageningen.
36. USDA Soil Survey Laboratory manual, Soil Survey Investigation Report no. 42, version 4, USDA-NRCS, Nebraska, USDA. 2004.

37. Walkley A, Black CA. An examination of Degtjareff methods for determining soil organic matter and the proposed modifications of the chromic acid titration method. Soil Sci. 1934; 37: 29–38. https://doi.org/10.1097/00010694-193401000-00003.
38. Bremmer JM, Mulvaney CS. Nitrogen total. In: Page, A.L. (Ed.), Method of Soil Analysis. Part 2. Chemical and Microbiological Properties. Am. Soc. Agron., WI, Agron. Monogr. 1982; 9: 595–624. doi.org/10.2134/agronmonogr9.2.2ed.c31.
39. Olsen SR, Cole CV, Watanabe FS, Dean LA. Estimation of available phosphorus in soils by extraction with sodium bicarbonate. United States Department of Agriculture Circular. Washington, DC; 1954; p. 939. https://doi.org/10.1002/jsfa.2740660423.
40. Jackson ML. Soil chemical analysis. New Delhi, India: Prentice- Hall of India Private Ltd.. 1967.https://doi.org/10.1002/jpln.19590850311.
41. Rowell DL. Soil science: Methods and application, Addison Wesley Longman, Limited, England. 1994.
42. Chapman HD. Cation exchange capacity. In C. A. Black (Ed.). Methods of soil analysis – Chemical and microbiological properties, Madison, WI: J Am Soc Agron. 1965; 9: 891–901. https://doi.org/10.2134/agronmonogr9.2.c6.
43. Gotway CA, Ferguson RB, Hergert GW, Peterson TA. Comparison of kriging and inverse-distance methods for mapping soil parameters. Am J Soil Sci. 1996; 60:1237–1247. https://doi.org/10.2136/sssaj1996.03615995006000040040x.
44. Wollenhaupt NC, Wolkowski RP, Clayton MK. Mapping soil test phosphorus and potassium for variable-rate fertilizer application. J Prod Agric. 1994; 7(4): 441-448. https: //doi.org/10.2134/jpa 1994.0441.
45. Robinson TP, Metternicht G. Comparing the performance of techniques to improve the quality of yield maps. Agric Syst. 2005; 85(1):19-41. https://doi.org/10.1016/j.agsy.2004. 07.010.

46. EthioSIS (Ethiopia Soil Information System). Soil fertility status and fertilizer recommendation atlas for Tigray regional state, Ethiopia. Addis Ababa, Ethiopia: Agricultural Transformation Agency. 2014; 76 pp.
47. Moraes JFL, Volkoff B, Cerri CC, Bernoux M. Soil properties under Amazon forest and changes due to pasture installation in Rondonia. Brazil. Geoderma. 1996; 70 (1): 63–81. https://doi.org/10.1016/0016-7061(95)00072-0.
48. Hazelton P, Murphy B. Interpreting soil test results: what do all the numbers mean? Australia: CSIRO Publishing, Collingwood Victoria–Australia. http://www.publish. CSIRO. 2007; 160 p. https://doi.org/10.1111/sum.12402.
49. Miheretu BA, Yimer AA. Spatial variability of selected soil properties to land-use and slope position in Gelana sub-watershed, Ethiopia's northern highlands. Phys Geogr. 2018; 39 (3): 230–245. https://doi.org/10.1080/02723646.2017.1380972.
50. Assefa A, van Keulen H. Modeling soil nutrient dynamics under alternative farm management practices in the Northern Highlands of Ethiopia. Soil Till Res. 2009; 103:203–215. https://doi.org/10.1016/j.still.2008.05.022.
51. Moges A, Dagnachew M, Yimer F. Land-use effects on soil quality indicators: a case study of Abo-Wonsho Southern Ethiopia. Appl Environ Soil Sci. 2013. https://doi.org/10. 1155/2013/784989.
52. Food and Agriculture Organization (FAO). Soil and plant testing as a basis of fertilizer recommendations. Soils Bulletin No. 38/2. Rome. 1980; 100 pp.
53. Tellen VA, Yerima BPK. Effects of land-use change on soil physical and chemical properties in selected areas in Cameroon's North West region. Environ Syst Res. 2018; 7:3. https://doi.org/10.1186/s40068-018-0106-0.
54. Juo ASR, Manu A. Chemical dynamics in slash-and-burn agriculture. Agric Ecosyst Environ. 1996; 58:49–60. https://doi.org/10.1016/0167-8809 (95)00656-7.

55. Kassa Cholbe M, Kebede Yeme F, Haile Woldeyohannes W. Fertility Status of Acid Soils under Different Land-Use Types in Wolaita Zone, Southern Ethiopia. Appl Environ Soil Sci. 2020. https://doi.org/10.1155/2020/3713967.

56. McNaughton SJ, Banyikwa FF, McNaughton MM. Promotion of the cycling of diet-enhancing nutrients by African grazers. Science. 1997; 278: 1798–1800. https://doi.org/10.1126/ science.278.5344.1798.

57. Mesfin Abebe. The challenges and prospects of soil chemistry in Ethiopia. In: Teshome Yizengaw, Eyasu Mekonnen, and Mintesinot Behailu, editors. Proceedings of the 3rd Conference of the Ethiopian Society of Soil Science (ESSS). Addis Ababa, Ethiopia. Ethiopian Science and Technology Commission; 1996; p. 78-96.

58. Terefe H, Argaw M, Tamene L, Mekonnen K, Recha J, Solomon D. Effects of sustainable land management interventions on selected soil properties in Geda watershed, central highlands of Ethiopia. Ecol Process. 2020; 9(1): 1-11. https://doi.org/10.1186/s13717-020-02 16- 2.

59. Gol C, Cakir M, Edis S, Yilmaz H. The effects of land-use/land-cover change and demographic processes (1950–2008) on soil properties in the Gokcay catchment, Turkey, Afr J Agric Res. 2010; 4:1670–1677. https://doi.org/10.5897/AJAR09.716.

60. Kokeb A, Muche M, Molla E. Assessing the Physical and Chemical Properties of Soil under Different Land-Use Types. J Environ Anal Toxicol. 2015; 5(5):309. https://doi.org/10.4172/2161-0525.1000309.

61. Selassie YG, Anemut F, Addisu S. The effects of land-use types, management practices and slope classes on selected soil physical and chemical properties in Zikre watershed, North-Western Ethiopia. Environ Syst Res. 2015; 4:3. https://doi.org/10.1186/s40068-015-0027-0.

62. Molla FE, Yalew AM. The effects of land-use types and soil depth on soil properties of Agedit watershed, Northwest. Ethiopia. Ethiop J Sci & Technol. 2018; 11(1): 39-56. https://doi.org/10.4314/ejst.v11i1.4.

63. Vågen TG, Winowiecki LA. Mapping of soil organic carbon stocks for spatially explicit assessments of climate change mitigation potential, Environ Res Lett. 2013; 8:1–3. https://doi .org/10.1088/1748-9326/8/1/015011.

64. Jahn R, Blume HP, Asio VB, Spaargaren O, Schad P. Guidelines for soil description. 4th ed**.** Rome, FAO. 2006; 97 pp.

65. Warrick AW. Spatial variability. In: Hillel D, editor. Environmental soil physics. Cambridge (MA): Academic Press; 1998; pp. 655–675.

66. Tadele A, Aemro T, Yihenew GS, Birru Y, Wolfgramm B, Hurni H. Soil properties and crop yields along the Terraces and toposequences of Anjeni watershed, Central Highlands of Ethiopia. J Agric Sci. 2013; 5:2. https://doi.org/10.5539/jas.v5n2p134.

67. Braimoh AK, Vlak PL. The impact of land-cover change on soil properties in northern Ghana. Land Degrad Dev. 2004; 15: 65–74. https://doi.org/10.1002/ldr.590.

68. Muñoz- Rojas M., Jordán A., Zavala LM., De la Rosa D., Abd- Elmabod SK, Anaya- Romero M. Impact of land-use and land-cover changes on organic carbon stocks in Mediterranean soils (1956–2007). Land Degrad Dev. 2015; 26(2): 168-179. https://doi. org/10.1002/ldr.2194.

69. Chemura A, Kutywayo D, Chagwesha T, Chidoko P. An Assessment of Irrigation Water Quality and Selected Soil Parameters at Mutema Irrigation Scheme, Zimbabwe. J Water Resource Prot. 2014; 6: 132-140. https://doi.org/10.4236/jwarp.2014.62018.

70. Tubiello FN, Salvatore M, Condor Golec R, Rossi S, Ferrara A, Biancalani R et al.. The contribution of agriculture, forestry, and other land-use activities to global warming, 1990 –2012. Glob Change Biol. 2015; 21(7): 2655–2660. https://doi:10.1111/gcb.12865.

71. Galloway JN, Townsend AR, Erisman JW, Bekunda M, Cai Z, Freney JR et al.. Transformation of the nitrogen cycle: recent trends, questions, and potential solutions. Science. 2008; 320:889–92. https://doi:10.1126/science.1136674.
72. Tian HQ, Lu CQ, Melillo J, Ren R, HuangY, Xu XF et al.. Food benefit and climate warming potential of nitrogen fertilizer uses in China. Environ Res Lett. 2012; 7. https://doi:10.1088/1748-9 326/7/4/044020.
73. Food and Agriculture Organization (FAO). Integrated soil management for sustainable agriculture and food security in Southern and East Africa, FAO, Rome, Italy. 1999.
74. Novara A, Gristina L, Guaitoli F, Santoro A, Cerdà A. Managing soil nitrate with cover crops and buffer strips in Sicilian vineyards, Solid Earth. 2013; 4(2): 255–262. https://doi.org/10.5194/se-4-255-2013.
75. Venterea RT, Halvorson AD, Kitchen N, Liebig MA, Cavigelli MA, Del Grosso SJ, Motavalli PP, Nelson KA, Spokas KA, Singh BP, Stewart CE, Ranaivoson AZ, Strock JS, Collins H. Challenges and opportunities for mitigating nitrous oxide emissions from fertilized cropping systems. Front Ecol Environ. 2012; 10(10): 562-570. https://doi.org/10.1890/120062.
76. Snyder CS, Davidson EA, Smith P, Venterea RT. Agriculture: sustainable crop and animal production to help mitigate nitrous oxide emissions. Curr Opin Environ Sustain. 2014; 9-10: 46-54. https://doi.org/10.1016/j.cosust.2014.07.005.

Figure Click here to access/download;Figure;Fig 1.tif

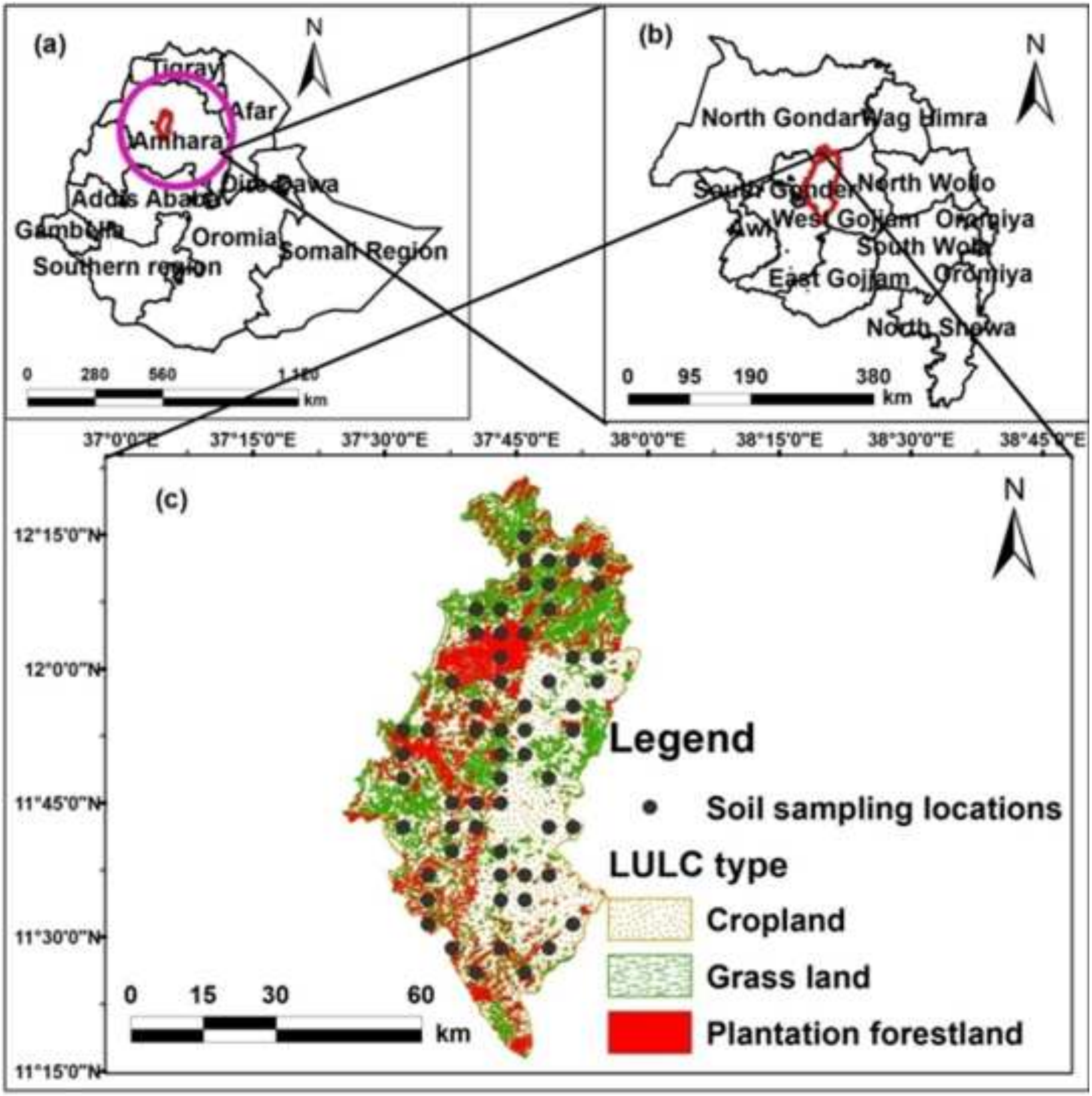

Fig 1. Location map of the study area namely (a) Ethiopia, (b) Amhara region, and (c) soil sampling locations across land-use/land-cover (LULC) types

Figure Click here to access/download;Figure;Fig 2.tif

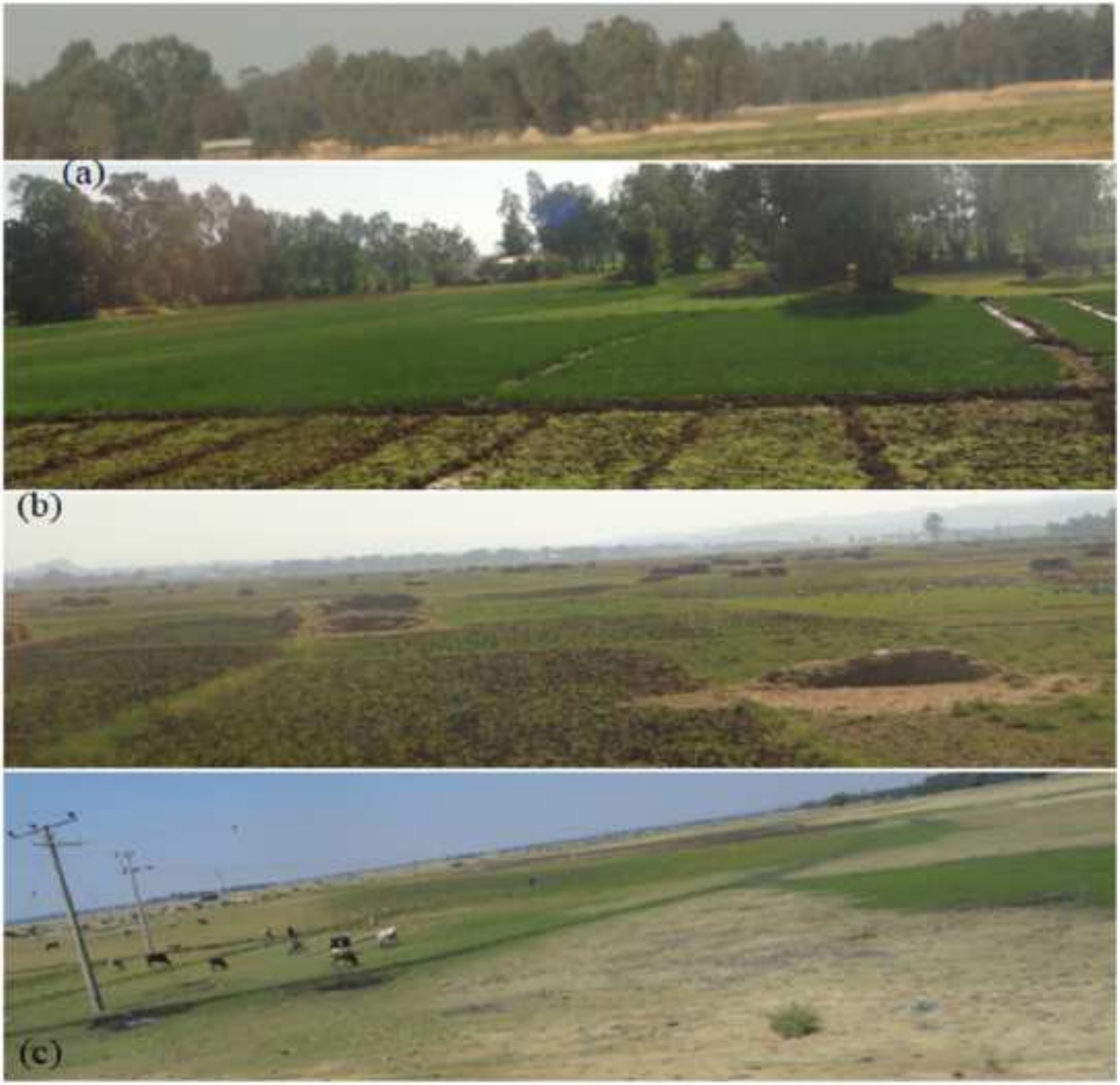

Fig 2. Major land-use/-cover types in the study area viz., (d) Rice, onion, and eucalyptus plantation forestland, (e) Rice cultivated land, and (F) grazing land

Figure Click here to access/download;Figure;Fig 3.tif

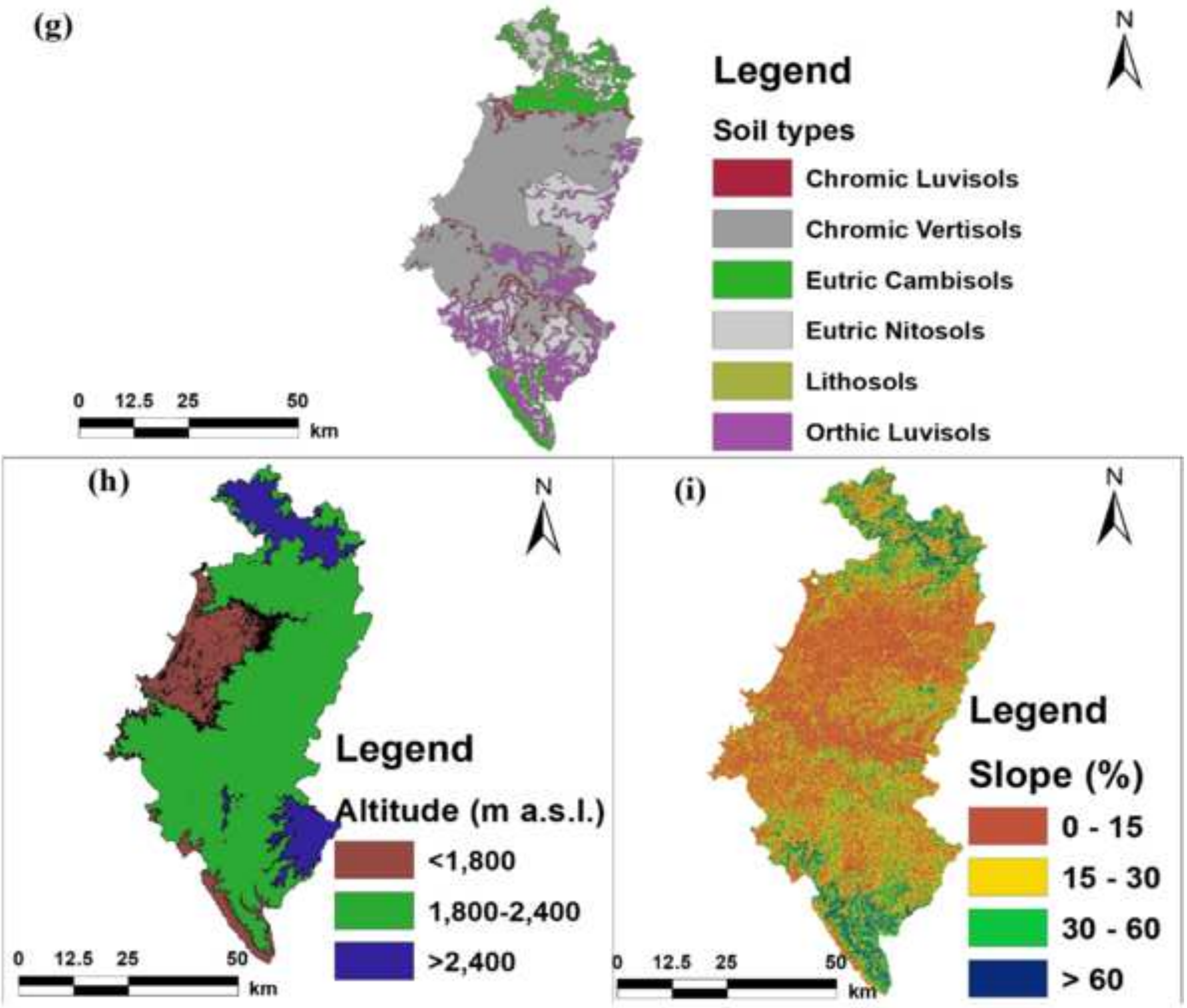

Fig 2. Different maps of the study area particularly (g) Soil types, (h) Altitude, and (i) Slope.

Figure Click here to access/download;Figure;Fig 4.tif

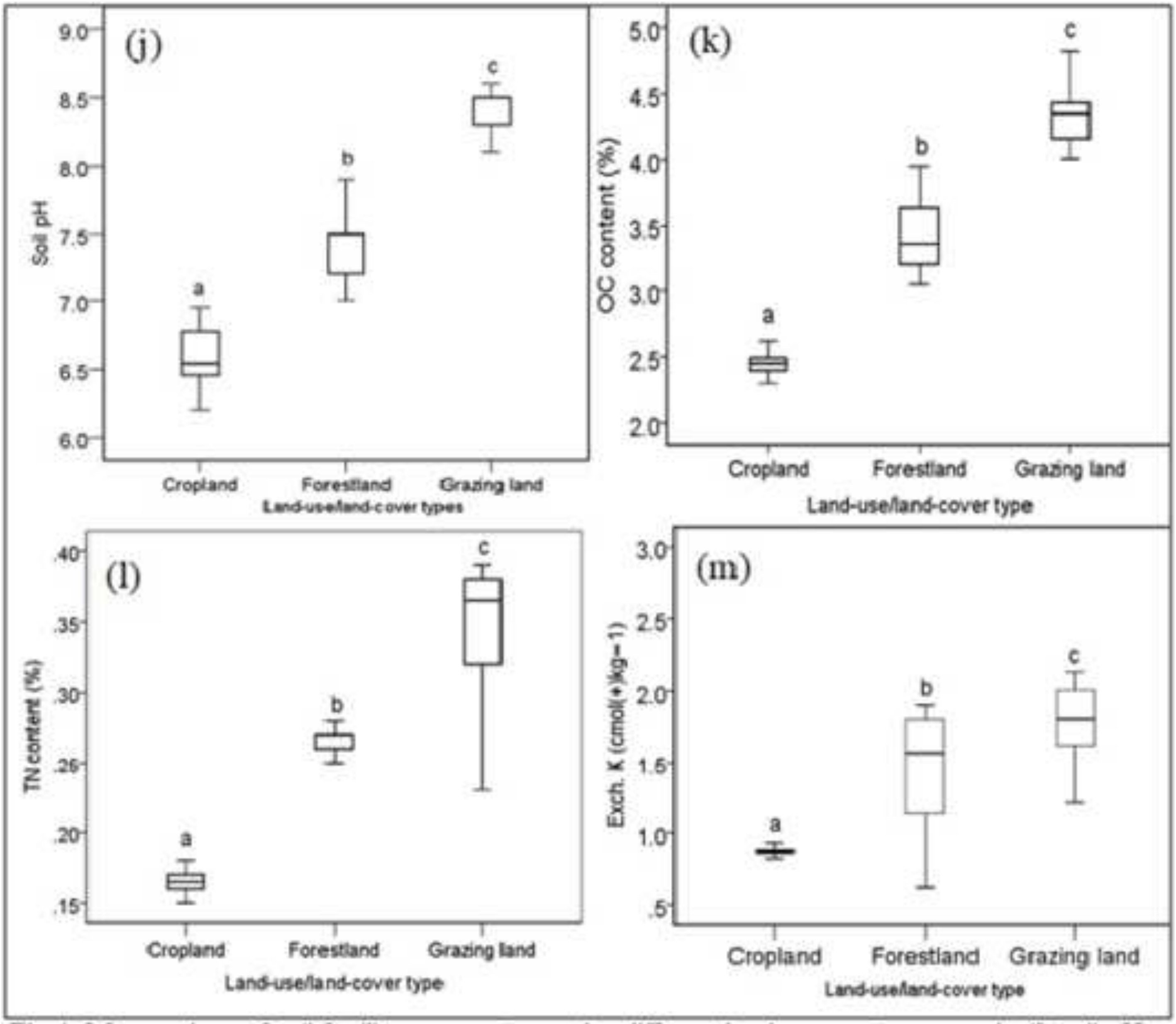

Fig 4. Mean values of soil fertility parameters under different land-use systems namely (j) soil pH, (k) organic carbon (OC), (l) Total nitrogen (TN), and (m) Exchangeable K under different land-use systems

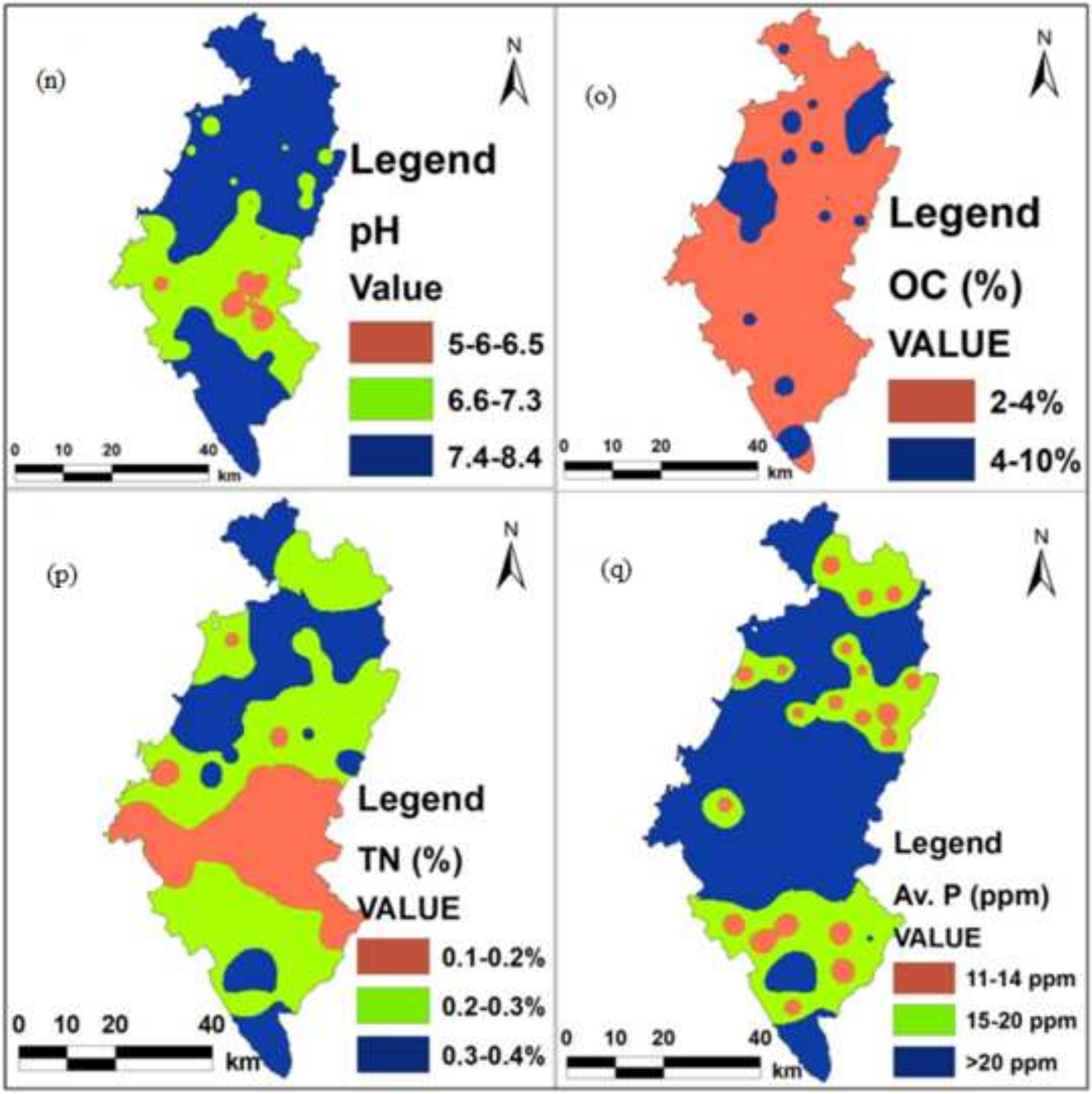

Fig. 5. Maps of soil parameters namely (n) Soil pH, (o) Organic carbon (%), (p) Total nitrogen (%), and (q) Available phosphorus (ppm)

Figure Click here to access/download;Figure;Fig 6.tif

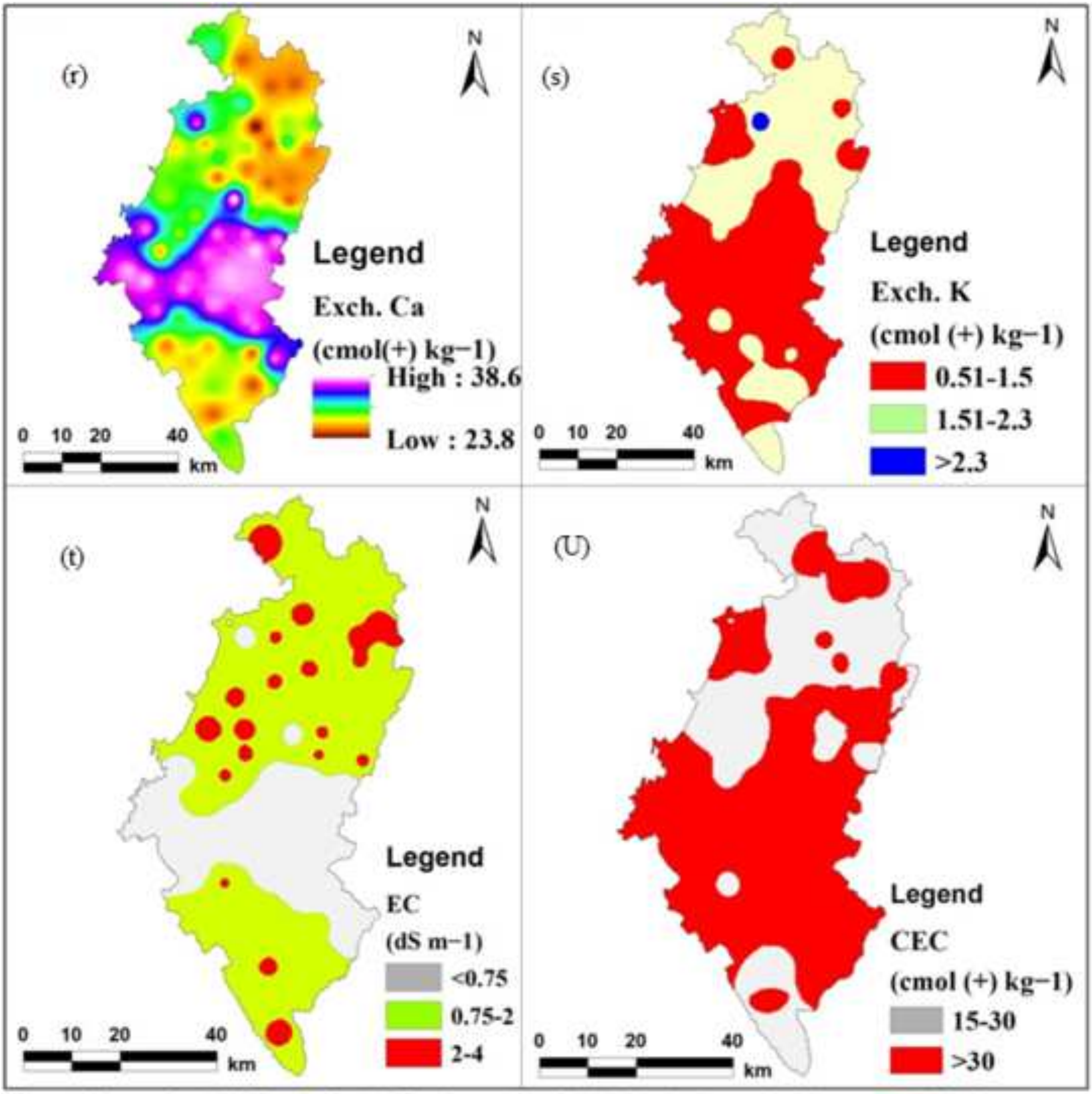

Fig. 6 . Maps of soil (r) Exch. Ca, (s) Exchangeable K, (t) Electrical conductivity, and (u) Cation exchange capacity

Click here to access/download
Supporting Information
S1 Table.xlsx

Click here to access/download
Supporting Information
S1 Appendix.docx
