# Supplementary material for: Spatial variability of soil chemical properties under different land-uses in Northwest Ethiopia
Source: PLoS One. 2021 Jun 23;16(6):e0253156. doi: 10.1371/journal.pone.0253156 (PMC8241222; doi:10.1371/journal.pone.0253156)
Supplement: Attachment — Submitted filename: PONE-D-21-03433_R2.pdf [file pone.0253156.s006.pdf]

PLOS ONE

Spatial variability of soil chemical properties under different land-uses in Northwest Ethiopia

--Manuscript Draft--

**Manuscript Number:**	PONE-D-21-03433R1
**Article Type:**	Research Article
**Full Title:**	Spatial variability of soil chemical properties under different land-uses in Northwest Ethiopia
**Short Title:**	Spatial variability of soil chemical properties under different land-uses in Northwest Ethiopia
**Corresponding Author:**	Gizachew Ayalew Tiruneh, MSc, PhD candidate<br>Bahir Dar University College of Agriculture and Environmental Sciences<br>Bahir Dar, ETHIOPIA
**Keywords:**	Inverse Distance Weighting; land-use; soil properties; spatial variability
**Abstract:**	The understanding of the spatial variation of soil chemical properties is critical in agriculture and the environment. To assess the spatial variability of soil chemical properties in the Fogera plain, Ethiopia, we used Inverse Distance Weighting (IDW), pair-wise comparisons, descriptive analysis, and principal component analysis (PCA). In 2019, soil samples were collected at topsoil (a soil depth of 0-20 cm) from three representative land-uses (cropland, plantation forestland, and grazing lands) using a grid-sampling design. The variance analysis for soil pH, available phosphorus (avP), organic carbon (OC), total nitrogen (TN), electrical conductivity (EC), exchangeable potassium (exchK), exchangeable calcium (exchCa), and cation exchange capacity (CEC) revealed significant differences between the land-uses. The highest mean values of pH (8.44), avP (31.51 ppm), OC (3.49 %), TN (0.313 %), EC (2.22 dS m −1), and exchK (3.6 cmol(+) kg -1) were determined under grazing land. The lowest pH (6.58), OC (2.44 %), TN (0.17 %), exchK (0.87 cmol(+) kg -1), and EC (0.13 dS m −1) were recorded in cultivated land. The PCA result revealed that the land-use change was responsible for most soil chemical properties, accounting for 98.55 %. Soil maps can help identify the nutrient status, update management options, and increase productivity and profit. Moreover, it promotes soil health and reduces environmental effects. The expansion of cultivated lands resulted in a significant decrease in soil organic matter. Thus, soil management strategies should tailor to replenish soil nutrient content while maintaining agricultural productivity in the Fogera plain.
**Order of Authors:**	Gizachew Ayalew Tiruneh, MSc, PhD candidate
	Tiringo Yilak Alemayehu
	Derege Tsegaye Meshesha
	Eduardo Saldanha Vogelmann
	José Miguel Reichert
	Nigussie Haregeweyn
**Response to Reviewers:**	Date: April 24 09, 2021<br>Rebuttal letter<br>PONE-D-21-03433<br><br>We are glad about the academic editor and the reviewers' comments, which strengthen the current version of the manuscript "Spatial variability of soil properties under different land-uses in Northwest Ethiopia". In addition, our utmost sincere gratitude goes to you and the reviewers who devote their valuable time to bring our manuscript to a qualified paper.<br><br>We have provided a one by one reply to all concerns and comments given below. We thank you for your consideration of this resubmission and look forward to your

response.
Best regards,
Gizachew Ayalew Tiruneh (on behalf of all co-authors)
Lecturer in Debre Tabor University
Ph.D. Fellow in soil science, Bahir Dar University
Email: tiruneh1972@gmail.com

Editor’s comments
Comments 1: A rebuttal letter that responds to each point raised by the academic editor and reviewer(s). You should upload this letter as a separate file labeled 'Response to Reviewers'.
Response: We addressed the concerns provided by the editor and reviewers and uploaded a file labeled “Response to Reviewers”.
Comments 2: A marked-up copy of your manuscript that highlights changes made to the original version. You should upload this as a separate file labeled 'Revised Manuscript with Track Changes'.
Response: We tried to do it.
Comments 3: An unmarked version of your revised paper without tracked changes. You should upload this as a separate file labeled 'Manuscript'.
Response: We have addressed accordingly.
Comments 4: If you would like to make changes to your financial disclosure, please include your updated statement in your cover letter.
Response: We have not made any changes to financial disclosure.
Comments 5: Guidelines for resubmitting your figure files are available below the reviewer comments at the end of this letter.
Response: We made corrections as per the guidelines
Comments 6: If applicable, we recommend that you deposit your laboratory protocols in protocols.io to enhance the reproducibility of your results. Protocols.io assigns your protocol its own identifier (DOI) so that it can be cited independently in the future. For instructions see: http://journals.plos.org/plosone/s/submission-guidelines#loc-laboratory-protocols. Additionally, PLOS ONE offers an option for publishing peer-reviewed Lab Protocol articles, which describe protocols hosted on protocols.io. Read more information on sharing protocols at
https://plos.org/protocols?utm_medium=editorial-email&utm_source=authorletters&utm_campaign=protocols.
Response: The majority of our protocols involve standard methods such as soil pH, available phosphorus (av. P), organic carbon (OC), total nitrogen (TN), electrical conductivity (EC), exchangeable Ca, and K, and cation exchange capacity (CEC) measurement. We have specified our Lab protocol in the revised manuscript [reference #45].
Comments 7: When submitting your revision, we need you to address these additional requirements, please ensure that your manuscript meets PLOS ONE's style requirements, including those for file naming. The PLOS ONE style templates can be found at

https://journals.plos.org/plosone/s/file?id=wjVg/PLOSOne_formatting_sample_main_body.pdf and
https://journals.plos.org/plosone/s/file?id=ba62/PLOSOne_formatting_sample_title_authors_affiliations.pdf
Response: We addressed PLOS ONE's style requirements in this revision.
Comments 8: Please include a separate caption for each figure in your manuscript.
Response: We addressed PLOS ONE's style requirements in this revision using PACE.
Comments 9: Please ensure that you refer to Figures 5 and 6 in your text as, if accepted, production will need this reference to link the reader to the figure.
Response: We cited the figures in manuscript’s text.
Comments 10: Please include captions for your Supporting Information files at the end of your manuscript, and update any in-text citations to match accordingly.
Response: We followed PLOS ONE's Supporting Information guidelines to include the captions.
Reviewers' comments:

Reviewer #1:
Comments 1: 1. Is the manuscript technically sound, and do the data support the

conclusions?
The manuscript must describe a technically sound piece of scientific research with data that supports the conclusions. Experiments must have been conducted rigorously, with appropriate controls, replication, and sample sizes. The conclusions must be drawn appropriately based on the data presented.
Reviewer #1: No
Reviewer #2: Partly
Response: Dear Reviewers, thank you so much for taking your valuable time to elevate the quality of our manuscript. We do hope that the Reviewer's concerns will be addressed.

____

2. Has the statistical analysis been performed appropriately and rigorously?
Reviewer #1: No
Reviewer #2: Yes
Response: Thank you. We have gone thoroughly the revised manuscript, and hopefully that the first Reviewer will be satisfied.

____

3. Have the authors made all data underlying the findings in their manuscript fully available?
The PLOS Data policy requires authors to make all data underlying the findings described in their manuscript fully available without restriction, with rare exception (please refer to the Data Availability Statement in the manuscript PDF file). The data should be provided as part of the manuscript or its supporting information, or deposited to a public repository. For example, in addition to summary statistics, the data points behind means, medians and variance measures should be available. If there are restrictions on publicly sharing data—e.g. participant privacy or use of data from a third party—those must be specified.
Reviewer #1: Yes
Reviewer #2: Yes
Thank you.

____

4. Is the manuscript presented in an intelligible fashion and written in standard English?
PLOS ONE does not copyedit accepted manuscripts, so the language in submitted articles must be clear, correct, and unambiguous. Any typographical or grammatical errors should be corrected at revision, so please note any specific errors here.
Reviewer #1: No
Reviewer #2: Yes
Response: Thank you. We have thoroughly revised our manuscript with the help of Grammarly (premium) and Turnitin software, and we do hope that the first Reviewer's concerns will be addressed.

____

5. Review Comments to the Author
Reviewer #1:
The manuscript "Spatial variability of soil properties under different land-uses in Northwest Ethiopia" is well written. In my opinion, the manuscript is relevant and appropriate for the PlosONE journal. Nonetheless, I have the following suggestions that should be addressed by the authors before to publish:
Title: please add 'chemical' in title
[Not a comment, but you have not defined paragraph sign meaning in author name list- double check]
Response: Thank you. We have tried to add 'chemical' the title in the way this reviewer has suggested.
L#21-23: these are not only procedure to evaluate soil variability, so please make a general sentence but focus as background information or rationale of the study
Response: Thank you. We have revised the background information or rationale of the study.
L#24: Include depth of topsoil
Abstract is poorly written. No clear explanation of measured variables. What was the tool(s) used to explore spatial variability of study area? Only descriptive statistics?
Response: Thank you. We have included depth of topsoil (0-20 cm) and have revised the Abstract section. We also incorporated more ideas on geo-statistical (IDW) tool and others (ANOVA, box plots, and PCA). We hope that this revised version will be satisfying.

L#38: what do you mean by inappropriate?
Response: Thank you. We replaced “inappropriate” by “irrelevant” as shown in [L43] of the revised manuscript.
L#43: please double check your citation style for [14] based on journal guidelines
Response: Thank you. The reference style is now made consistent, reference # 14 (in the revised manuscript) with others.
L#58-61: Besides scholars, your study should be valuable to farmers as well. Please think about other site-specific recommendation practices and mention it in Introduction section because there is not only geostatistics for optimizing nutrient use and improve crop production. Countries like, Nigeria, India, Nepal have used soil testing mobile van as well. I am not sure about similar approach in Ethiopia, but it is suggested to visit this link (https://doi.org/10.1080/15427528.2017.1387837) for more information.
Response: Thank you. We appreciate your valuable recommendation and thank for showing this important link. We mentioned some site-specific recommendation practices and included in Introduction section. However, Ethiopia has not yet used mobile van so far for soil testing like the above-mentioned countries.
L#111: Is this correct amount? 20-50 mg of dried soil; please cross-check with this information:
https://www.environment.nsw.gov.au/resources/soils/testmethods/oc.pdf
Response: Thank you for indicating this useful source. We have added a relevant source [reference # 45] to get more elaboration on the idea of “the amount of dried soil required in examining OC [L145 in the revised manuscript].
L#136-139: Why there is not any information about tools of spatial interpolation in Introduction section? You need to re-structure your Introduction and Methodology (statistical analysis) sections.
No justification- why did you select IDW only, why not others?
Results: there is repetition of information (results) in table and text many times. Also there is no any value of Table 8, just include mean and SE, and then lsd comparison. If you have this already, then delete Table 8.

Response: Thank you for the suggestion. We share with your concerns. We tried to incorporate information about tools of spatial interpolation in Introduction section. We also re-structured the Introduction and Methodology (statistical analysis) sections. The justification of IDW selection was included in Introduction section and Methodology section. The repetition of information (results) in table and text were minimized and Table 8 was removed.

L#251-252: How GHG information relates to your current study? Do not bring larger picture in your Discussion section. Please focus on that what you have done and its implications.

Response: Thank you for the concern. We added some relevant ideas of GHGs and its implications in Abstract, Introduction, Discussion, and Conclusion sections.
L#255: what is best land planning?
Response: Thank you for the comment. We replaced “relevant” instead of “best” in the phrase “best land planning” (L296). A relevant land-use planning gives time and resources to decision-making processes in order to reach conclusions on suitable or best possible use of land based on long-term objectives and benefits that are more equitable.
Reviewer #2:
The authors presented a work on "Spatial variability of soil properties under different land-uses in Northwest Ethiopia" but however dwelt so much on land use instead of the spatial variability. The authors use only 60 samples to produce the map of the distribution of soil chemical properties in the study location of more than 6,000 ha. This to me is not acceptable because the samples are too few to highlight the spatial variability in the soil properties. Even the map produced was not mentioned in the abstract let alone discussing them in the results and Discussion section. A number of information that is supposed to be in the abstract are missing. There were a lot of long sentences that need to be summarized or broken into two or more sentences.
Response: Thank you for the concern. In considering the soil variability, composite and purposive soil sampling was employed to reduce sampling intensity. Moreover, most coverage of the study area is gentle slope, bordering Lake Tana. Supportive references were also cited [43-44].
a soil sampler per 100 ha was used in India [#reference 43] in revised manuscript

a soil sample per 625 ha was taken in Turkey [#reference 44] in revised manuscript
We included the soil variability and soil maps in the Abstract, Methodology, Results, discussion, and conclusion sections and long sentences were also shortened.

____

6. PLOS authors have the option to publish the peer review history of their article (what does this mean?). If published, this will include your full peer review and any attached files.
If you choose "no", your identity will remain anonymous but your review may still be made public.
Do you want your identity to be public for this peer review? For information about this choice, including consent withdrawal, please see our Privacy Policy.
Reviewer #1: No
Reviewer #2: No
[NOTE: If reviewer comments were submitted as an attachment file, they will be attached to this email and accessible via the submission site. Please log into your account, locate the manuscript record, and check for the action link "View Attachments". If this link does not appear, there are no attachment files.]
While revising your submission, please upload your figure files to the Preflight Analysis and Conversion Engine (PACE) digital diagnostic tool, https://pacev2.apexcovantage.com/. PACE helps ensure that figures meet PLOS requirements. To use PACE, you must first register as a user. Registration is free. Then, login and navigate to the UPLOAD tab, where you will find detailed instructions on how to use the tool. If you encounter any issues or have any questions when using PACE, please email PLOS at figures@plos.org. Please note that Supporting Information files do not need this step.
Response: Thank you. We have used PACE with this submission, so this should be right.

____

Please note that once again, thank you very much. Your comments are greatly appreciated.

Best regards,
Gizachew Ayalew Tiruneh (on behalf of all co-authors)
Lecturer in Debre Tabor University
Ph.D. Fellow in soil science, Bahir Dar University
Email: tiruneh1972@gmail.com

Additional Information:

Question	Response
**Financial Disclosure**<br><br>Enter a financial disclosure statement that describes the sources of funding for the work included in this submission. Review the submission guidelines for detailed requirements. View published research articles from *PLOS ONE* for specific examples.<br><br>This statement is required for submission and **will appear in the published article** if the submission is accepted. Please make sure it is accurate.	The first author is a Ph.D. candidate in soil science at Bahir Dar University, Ethiopia. Our grateful acknowledgment directs to Debre Tabor University and Bahir Dar University for the financial grant. The Amhara Regional Soil Laboratory Center was substantially recognized for laboratory analysis for this study. Water and Land Resource Centre (WLRC) and Ethiopian Mapping Agency (EMA) are also acknowledged for various data access. The funders had no role in study design, data collection and analysis, decision to publish, or preparation of the manuscript.

Powered by Editorial Manager® and ProduXion Manager® from Aries Systems Corporation

**Unfunded studies**<br>Enter: *The author(s) received no specific funding for this work.*<br><br>**Funded studies**<br>Enter a statement with the following details:<br>• Initials of the authors who received each award<br>• Grant numbers awarded to each author<br>• The full name of each funder<br>• URL of each funder website<br>• Did the sponsors or funders play any role in the study design, data collection and analysis, decision to publish, or preparation of the manuscript?<br>• **NO** - Include this sentence at the end of your statement: *The funders had no role in study design, data collection and analysis, decision to publish, or preparation of the manuscript.*<br>• **YES** - Specify the role(s) played.<br><br>* typeset	
**Competing Interests**<br><br>Use the instructions below to enter a competing interest statement for this submission. On behalf of all authors, disclose any competing interests that could be perceived to bias this work—acknowledging all financial support and any other relevant financial or non-financial competing interests.<br><br>This statement is required for submission and **will appear in the published article** if the submission is accepted. Please make sure it is accurate and that any funding sources listed in your Funding Information later in the submission form are also declared in your Financial Disclosure statement.<br><br>View published research articles from *PLOS ONE* for specific examples.	The authors have declared that no competing interests exist.

**NO authors have competing interests**<br><br>Enter: *The authors have declared that no competing interests exist.*<br><br>**Authors with competing interests**<br><br>Enter competing interest details beginning with this statement:<br><br>*I have read the journal's policy and the authors of this manuscript have the following competing interests: [insert competing interests here]*<br><br>* typeset	
**Ethics Statement**<br><br>Enter an ethics statement for this submission. This statement is required if the study involved:<br><br>• Human participants<br>• Human specimens or tissue<br>• Vertebrate animals or cephalopods<br>• Vertebrate embryos or tissues<br>• Field research<br><br>Write "N/A" if the submission does not require an ethics statement.<br><br>General guidance is provided below. Consult the submission guidelines for detailed instructions. **Make sure that all information entered here is included in the Methods section of the manuscript.**	Debre Tabor University's Research and Publication Directorate and Bahir Dar University's Research and Publication Directorate authorized the present study to collect soil samples and access the field site. Farmers agreed to collect the soil samples in the study area, as the survey has no harmful effects on humans.

**Format for specific study types**<br><br>**Human Subject Research (involving human participants and/or tissue)**<br>• Give the name of the institutional review board or ethics committee that approved the study<br>• Include the approval number and/or a statement indicating approval of this research<br>• Indicate the form of consent obtained (written/oral) or the reason that consent was not obtained (e.g. the data were analyzed anonymously)<br><br>**Animal Research (involving vertebrate animals, embryos or tissues)**<br>• Provide the name of the Institutional Animal Care and Use Committee (IACUC) or other relevant ethics board that reviewed the study protocol, and indicate whether they approved this research or granted a formal waiver of ethical approval<br>• Include an approval number if one was obtained<br>• If the study involved *non-human primates*, add *additional details* about animal welfare and steps taken to ameliorate suffering<br>• If anesthesia, euthanasia, or any kind of animal sacrifice is part of the study, include briefly which substances and/or methods were applied<br><br>**Field Research**<br><br>Include the following details if this study involves the collection of plant, animal, or other materials from a natural setting:<br>• Field permit number<br>• Name of the institution or relevant body that granted permission	
**Data Availability**<br><br>Authors are required to make all data underlying the findings described fully available, without restriction, and from the time of publication. PLOS allows rare exceptions to address legal and ethical concerns. See the PLOS Data Policy and FAQ for detailed information.	Yes - all data are fully available without restriction

A Data Availability Statement describing where the data can be found is required at submission. Your answers to this question constitute the Data Availability Statement and **will be published in the article**, if accepted.<br><br>**Important:** Stating 'data available on request from the author' is not sufficient. If your data are only available upon request, select 'No' for the first question and explain your exceptional situation in the text box.<br><br>Do the authors confirm that all data underlying the findings described in their manuscript are fully available without restriction?	
**Describe where the data may be found in full sentences. If you are copying our sample text, replace any instances of XXX with the appropriate details.**<br><br>• If the data are **held or will be held in a public repository**, include URLs, accession numbers or DOIs. If this information will only be available after acceptance, indicate this by ticking the box below. For example: *All XXX files are available from the XXX database (accession number(s) XXX, XXX.*).<br>• If the data are all contained **within the manuscript and/or Supporting Information files**, enter the following: *All relevant data are within the manuscript and its Supporting Information files.*<br>• If neither of these applies but you are able to provide **details of access elsewhere**, with or without limitations, please do so. For example:<br><br>*Data cannot be shared publicly because of [XXX]. Data are available from the XXX Institutional Data Access / Ethics Committee (contact via XXX) for researchers who meet the criteria for access to confidential data.*<br><br>*The data underlying the results presented in the study are available from (include the name of the third party*	All relevant data are within the manuscript and its Supporting Information files.

and contact information or URL).<br>• This text is appropriate if the data are owned by a third party and authors do not have permission to share the data.<br><br>* typeset	
Additional data availability information:	

Powered by Editorial Manager® and ProduXion Manager® from Aries Systems Corporation

Manuscript Click here to access/download;Manuscript;Manuscript.docx

Spatial variability of soil chemical properties under different land-uses in Northwest Ethiopia

Gizachew Ayalew[1,#a¶], Tiringo Yilak[2¶], Derege Tsegaye Meshesha[3¶], Eduardo Saldanha Vogelmann[4¶], José Miguel Reichert[5¶], Nigussie Haregeweyn[6¶]

[1] Department of Natural Resources Management, Faculty of Agriculture and Environmental Sciences, Debre Tabor University, P. O. Box 272, Debre Tabor, Ethiopia

[2] Department of Plant Sciences, Faculty of Agriculture and Environmental Sciences, Debre Tabor University, P. O. Box 272, Debre Tabor, Ethiopia

[3] Geospatial Data and Technology Center, College of Agriculture and Environmental Sciences, Bahir Dar University, P. O. Box 5501, Bahir Dar, Ethiopia

[4] Biological Sciences Institute, Federal University of Rio Grande, São Lourenço do Sul, Brazil

[5] Soils Department, Federal University of Santa Maria (UFSM), Av. Roraima 1000, 97105-900 Santa Maria, RS, Brazil

[6] International Platform for Dryland Research and Education, Tottori University, Tottori, Japan

[#a] Current Address: Department of Natural Resources Management, College of Agriculture and Environmental Sciences, Bahir Dar University, P. O. Box 5501, Bahir Dar, Ethiopia

* Corresponding author: E-mail: tiruneh1972@gmail.com

Abstract

The understanding of the spatial variation of soil chemical properties is critical in agriculture and the environment. To assess the spatial variability of soil chemical properties in the Fogera plain, Ethiopia, we used Inverse Distance Weighting (IDW), pair-wise comparisons, descriptive analysis, and principal component analysis (PCA). In 2019, soil samples were collected at topsoil (a soil depth of 0-20 cm) from three representative land-uses (cropland, plantation forestland, and grazing lands) using a grid-sampling design. The variance analysis for soil pH, available phosphorus (avP), organic carbon (OC), total nitrogen (TN), electrical conductivity (EC), exchangeable potassium (exchK), exchangeable calcium (exchCa), and cation exchange capacity (CEC) revealed significant differences between the land-uses. The highest mean values of pH (8.44), avP (31.51 ppm), OC (3.49 %), TN (0.313 %), EC (2.22 dS m^{-1}), and exchK (3.6 cmol(+) kg^{-1}) were determined under grazing land. The lowest pH (6.58), OC (2.44 %), TN (0.17 %), exchK (0.87 cmol(+) kg^{-1}), and EC (0.13 dS m^{-1}) were recorded in cultivated land. The PCA result revealed that the land-use change was responsible for most soil chemical properties, accounting for 98.55 %. Soil maps can help identify the nutrient status, update management options, and increase productivity and profit. Moreover, it promotes soil health and reduces environmental effects. The expansion of cultivated lands resulted in a significant decrease in soil organic matter. Thus, soil management strategies should tailor to replenish soil nutrient content while maintaining agricultural productivity in the Fogera plain.

Keywords: Inverse Distance Weighting; land-use; soil properties; spatial variability

Introduction

Environmental degradation caused by irrelevant land-use is a global problem in sustainable agriculture. Land-use change markedly affects soil properties [1, 2]. Changing land-use from forest cover to plough-land may result in a decrease in soil fertility, nutrients, and thus productivity [3-6], as well as increased soil perturbation [5, 7-12].

Rapid population growth and environmental factors in Ethiopia have resulted in converting forestland and grassland to cultivated land [13]. The expansion of cultivated areas has a substantial influence on soil nutrient content [14]. [15] reported changes in the amount of soil organic carbon and total N due to changes in land-use and land-cover in the Gerado catchment, northeastern Ethiopia. [16] also investigated that deforestation has led to the deterioration of soil organic matter. As a result, soil nutrient deficiency is a critical problem in the country and a major crop production constraint [17-18].

Ethiopia has seen an increase in cultivated lands and eucalyptus plantations and decreased grazing lands because of population growth [13]. The eucalyptus plantation had a significant impact on soil properties [19-21]. [22] reported a reduction in soil organic carbon (OC), total nitrogen (TN), exchangeable cations, and cation exchange capacity (CEC) contents were owing to the shift of woodlands to croplands and grazing lands in the same country. [23] also found a decline in pH and soil organic matter content in cultivated land in Ethiopia's Kabe watershed. As a result, scientific records of spatial variability and distribution of soil properties among land-use shifts are critical for optimizing fertilizer use and increasing crop productivity [20].

In developing countries, including Ethiopia, land-use/land-cover change is a significant source of greenhouse gases (GHGs) such as carbon dioxide (CO_2), nitrous oxide (N_2O), and methane (CH_4) emissions [24]. Furthermore, nitrogen-containing fertilizers [25-26], tillage [26], and complete removal of vegetation and residues [27] have influenced

spatial variability, soil nutrient cycling, and GHGs emission. Thus, information on the spatial variability of soil due to land-use change is critical in this regard.

Accurate and scientific information about soils is essential for developing effective soil management techniques that sustain agricultural production while maintaining environmental quality. As a result, in Ethiopia, where the provision of soil information is scarce, assessing spatial variability distribution on soil properties to land use is critical for predicting rates of soil functions and processes. Furthermore, site-specific management of pH, organic carbon, available N, available P, and available K [28] improves input use efficiency [29], increases crop production economic returns, and reduces ecological risks [30].

Many researchers have recently used geostatistics to estimate the spatial variability of soil properties [31-34]. In geostatistics, the inverse distance weighting (IDW) model can be used to map the spatial distribution of any soil property measured for spatially distributed samples [35]. Understanding the spatial variability of soil properties [36] and developing site-specific recommendations [36-37] are critical for optimizing nutrient usage, improving crop performance, and minimizing environmental risks [38]. This information, however, is scarce, and there are few studies on state of the art and assessments on the situation of soil degradation in Ethiopia.

Furthermore, the spatial information produced using geostatistical techniques would be an input to improve food security and obtain sustainable yield in developing countries, including Ethiopia, which has never been thoroughly investigated using spatial prediction models [39]. Understanding of different land's soil fertility of various land-use types could be used to predict, monitor, and evaluate the effects of changes in land-use types on soil properties, scheming appropriate land-use planning, and sustaining the productivity of agriculture. In this regard, previous researches on Fogera plain area have not yet adequately

discussed the Fogera plain area. Thus, the target of this research paper was to assess the effects of land-use types on spatial variability and distribution of soil fertility qualities, such as pH, organic carbon (OC), total nitrogen (TN), available phosphorus (avP), exchangeable calcium (Ca^{2+}), exchangeable potassium (K^{+}), electrical conductivity (EC), and cation exchange capacity (CEC) in Fogera plain, the highland of Ethiopia.

Materials and methods

Description of the study area

The research was conducted in the Fogera plain area (37º 0' 0" E - 38º 45' 0" E and 11º 15' 0" N-12º 15' 0" N) in the northwest highland of Amhara region, Ethiopia (Fig 1). It has a total area of 5,646 hectares. The topography of the study area is flat land. Rice (*Oryza sativa* L.), maize (*Zea mays* L.), and *Teff* [*Eragrostis tef* (Zucc.)] are the main crops grown in the study region. Rice production (76 %) is primarily a subsistence farming operation in the study area. Crop residues are collected for use as fuelwood or animal feed. As a result, no crop residue remains in the field to serve as a source of organic amendments. Rice, onions, and eucalyptus products are essential sources of income for the local people.

Fig 1. The study area's location: (a) Ethiopia, (b) Amhara region, and (c) soil sampling.

Sources of spatial data and their extraction

The study area's land-use/land-covers for 2019 was derived from an Ethiopia Mapping Agency (EMA) 1:20,000 scale land-use/land-cover map, and the mainland-use system consisted of cropland (4,362.73 ha, 77.27 %), plantation forestland (6,96.29 ha, 12.33 %) and grassland (586.98 ha, 10.39 %) [40]. We also performed reconnaissance surveys from August to November/2019 to validate the map. Using ArcGIS software version 10.5, a digital elevation model (ASTER DEM) with a resolution of 20 * 20 m, downloaded from the EMA website [40], was used to generate elevation and slope of the study area.

As shown Figs 2-3, agroecology belonging to Kolla (<1800 m a.s.l.), Weyna dega (1,800–2,400 m a.s.l.), and dega (>2400 m a.s.l.) has an area share of 16.24 %, 71.93 %, and 11.83 %, respectively. According to the digital soil map obtained from Water and Land Resource Centre (WLRC), the soil types in the Fogera plain region are Chromic Vertisols (48.57 %), Eutric Nitosols (17.65 %), Orthic Luvisols (15.75 %), Eutric Cambisols (10.78 %), Chromic Luvisols (3.97 %), and Lithosols (3.27 %) [41].

Fig 2. Land-use of the area: (d) Eucalyptus plantation forestland, (e) Cultivated land, and (f) Grazing land.

Fig 3. Different maps of the study area, particularly (g) Soil types, (h) Altitude, and (i) Slope.

Soil sampling, laboratory analysis, and statistical analysis

We used the fishnet tool included with ArcGIS software version 10.5 to build a regularly spaced grid of sampling points on the land-uses in the study region. Following that, 60 representative soil samples (20 from each use: cropland, plantation forestland, and grazing land) were identified at topsoil (a depth of 0-20 cm) in February-April/2020 using a systematic purposive approach. The topsoil was chosen as plants and soil management practices most influence it. To record each grid center in the field and the latitudes, longitudes, and elevations of sampling points, a portable Global Positioning System (Garmin 60; 2 m accuracy) was used. Soil sampling locations were chosen to better reflect each land-use condition by taking topographic features and soil conditions into account [42]. Each soil sample was created by compositing five sub-samples, improving sampling intensity and lowering soil analysis costs [43-44]. A kilogram of soil sample was collected from each location, air-dried, ground using a mortar and pestle, and analyzed at the Amhara Regional Soil Laboratory Center following national standard research methods [45].

Soil pH was measured potentiometrically in H_2O at the soil to solution ratio (1: 2.5) using a combined glass electrode pH meter (Model CP-505, Zabrze ul, Poland)[46]. The electrical conductivity (EC) of the soil was determined using an EC meter at the soil to water ratio of 1: 5 (Orion Model 145, USA) [47]. The Walkley-Black procedures were used to measure soil organic carbon (OC). A weighed portion (1-2 gm) of the dried, ground soil samples were treated with 5 ml of 0.4 N potassium dichromate solution ($K_2Cr_2O_7$) followed by the addition of 10 ml of concentrated sulfuric acid. The mixture was gently mixed and left for 16-18 hours before being given 100 ml of triple-distilled water. The excess of dichromate was back-titrated with the standard 0.2 N ferrous ammonium sulfate solution. The acidic dichromate was blankly titrated with ferrous ammonium sulfate solution [45, 48].

The total nitrogen (TN) of the soils was determined through digestion, distillation, and titration procedures of the Kjeldahl using the Kjeldahl apparatus (Gallenhamp, USA) [49]. The soil's available phosphorus (avP) was measured using 0.5 M $NaHCO_3$, pH of 8.5, a soil to solution ratio of 1: 20 for half an hour. The (avP) was extracted with 1 M ammonium chloride, 0.5 M ammonium fluoride, 0.1 M sodium hydro-oxide (from Blulux Laboratory Reagent (p) Ltd), and the amount was measured using a spectrophotometer (UV1700, Japan) [50-51]. Exchangeable calcium (Ca), magnesium (Mg), potassium (K), and sodium (Na) were determined by saturating the soil samples with 1 M ammonium acetate solution at pH 7.0. Subsequently, Ca and Mg were determined using Perkin-Elmer Model 290 atomic absorption spectrophotometer (ColVisTec, Germany); while exchangeable Na and K were measured using a Model 18 Perkin-Elmer flame photometer [52].

The soil's cation exchange capacity (CEC) was calculated by replacing NH_4^+ saturated samples with K^+ from a percolated KCl solution (from LOBA CHEMIE PVT.LTD). Washing with ethanol (from LOBA CHEMIE PVT.LTD) eliminated excess salt, and NH_4^+ was displaced by K^+ [53]. Merck KGaA and Sigma-Aldrich, Steinheim, Germany

supplied all of the chemicals and reagents, such as potassium dichromate, sulphuric acid, ferrous sulfate, ferroin, sodium bicarbonate, ammonium chloride, ammonium fluoride, and ammonium acetate, unless otherwise mentioned.

The variation of soil organic carbon, total nitrogen, and potassium was defined using the Inverse Distance Weighting (IDW) model [54-55]. Furthermore, IDW has been widely used by scholars for the prediction of soil OM and soil nitrate [56], P and K levels [57], and soil pH scale [58]. The current study used IDW to map the spatial distribution of the soil chemical properties under the ArcGIS environment. Besides, pair-wise comparisons, descriptive analysis, and principal component analysis (PCA) were performed using Statistical Analyses System (SAS) software version 9.4 and the Statistical Package for Social Sciences (SPSS) software version 24, respectively. Means were compared through the Tukey test at 1% probability.

Ethics statement

Debre Tabor University's Research and Publication Directorate and Bahir Dar University's Research and Publication Directorate authorized the present study to collect soil samples and access the field site. Farmers agreed to collect the soil samples in the study area, as the survey has no harmful effects on humans.

Results and discussion

Effect of land-use/land-cover types on soil fertility quality

Soil pH_{H2O}

The pH of the soil varied significantly ($P < 0.05$) depending on land-use. The soil pH values were found to be the highest (8.44) and the lowest (6.58) under the grazing and the cultivated lands, respectively (Table 1 and Figs 4-5). According to [59], higher soil pH levels obtained from plantation forestland and grazing land may be due to the presence of basic cations emanated from weathering [23] and the potash obtained from ashes [60].

Table 1. Soil pH_{H2O} rating, its area share (ha, %) by land-use, and its parametric test.

Common name	pH rating	Area share (ha, %) by land-use					
		Cropland		Plantation forestland		Grazing land	
		Area (ha)	%	Area (ha)	%	Area (ha)	%
Moderately acid	5.6-6.5	181.84	4.17	1.11	0.16	9.50	1.62
Neutral	6.6-7.3	1,576.93	36.15	139.05	19.97	173.04	29.48
Moderately alkaline	7.4-8.4	2,603.96	59.69	556.12	79.87	404.44	68.90
Total		4,362.73	100	696.29	100	586.98	100
Mean pH		6.58^{a}		7.40^{b}		8.44^{c}	

Means of pH with different letters are significantly varied (Tukey, $p < 0.01$).

Available phosphorus (avP)

Soil avP varied significantly according to land-uses. Following the limit established by [61], very high (>20 ppm) avP content was observed in soils of all land-uses (Table 2 and Fig 4). Higher avP values in soils could be revealed by the recurrent use of mineralized phosphorus [38], and the addition of manure, compost, and ashes [62], presence of weathered soil minerals [63], and actions of microbes. Higher levels of avP in the soils indicate that the soils have optimum nutrients for crop growth. [22, 64] reported similar findings in Ethiopia.

Table 2. Soil available phosphorus (avP) rating and its area share (ha, %) by land-use and its parametric test.

Common name	AvP rate	Area share (%) by land-use					
		Cropland		Plantation forestland		Grazing land	
	ppm	Area (ha)	Area (%)	Area (ha)	Area (%)	Area (ha)	Area (%)
Medium	11-14	273.66	6.27	35.02	5.03	23.05	3.93
High	15-20	1,425.74	32.68	200.90	28.85	118.12	20.12
Very high	>20	2,663.33	61.05	460.37	66.12	445.82	75.95
Total		4,362.73	100	696.29	100	586.98	100
Mean avP (ppm)		21.32^{a}		11.43^{b}		32.52^{c}	

Means of available P with different letters are significantly different (Tukey, $p < 0.01$).

Fig 4. Maps of (n) Soil pH, (o) Organic carbon, (p) Total nitrogen, and (q) Available phosphorus.

Soil organic carbon (OC) and total nitrogen (TN)

Land-use changes caused a significant difference in soil organic carbon (OC) and total nitrogen (TN). According to the ranking set by [65], low organic carbon content (2.44 %) in the soils dominated the agricultural land (87.66 %), grazing land (81.7 %), and eucalyptus plantation forestland (80.63 %), as shown in Table 3 and Figs 4-5). The highest (4.35 %) and lowest (2.44 %) OC contents obtained in grazing and croplands demonstrated that soil OC showed a better response to land-use type. It may be attributed to high erosion rates, crop residue exclusion, increased mineralization rates, and nutrient deficiency [38, 66]. The higher organic carbon and available P contents of the grazing lands suggest that OM is the primary source of avP [67].

Table 3. Soil organic carbon (OC) rating and its area share (ha, %) by land-use and its parametric test.

Common name	OC rate	Area share (ha, %) by land-use					
		Cropland		Plantation forestland		Grazing land	
	%	Area (ha)	Area (%)	Area (ha)	Area (%)	Area (ha)	Area (%)
Low	2-4	3,824.5	87.66	561.44	80.63	479.59	81.70
Medium	4-8	538.23	12.34	134.85	19.37	107.40	18.30
Total		4,362.73	100	696.29	100	586.98	100
Mean OC (%)		2.44^{a}		3.43^{b}		4.35^{c}	

Means of soil OC with different letters are significantly different (Tukey, $p < 0.01$).

Fig 5. Mean values of soil parameters using box plots.

According to the rate of [61], the cropland and plantation forestland demonstrated, respectively, low (0.17 %) and high total nitrogen (0.35 %) contents in the soils (Table 4 and Figs 4-5). In line with this, croplands had lower soil OC content [2, 23, 68]. Higher soil OC and TN contents found in plantation forests and grazing lands are most likely due to grass burning and dung deposition, respectively. Furthermore, researchers have advocated for grazing to sustain nutrient cycling and decomposition rates [69]. The low total nitrogen content may be eligible to minimize nitrogen loss by volatilization or leaching and rapid decomposition of OM. Hence, the conversion of grassland and eucalyptus plantation to cultivated land worsens soil OC and TN decline [70]. Thus, for long-term development, the soils need external nitrogen and carbon inputs.

Table 4. Soil total nitrogen (TN) rating and its area share (ha, %) by land-use and its parametric test.

Common name	TN rate	Area share (ha, %) by land-use					
		Cropland		Plantation forestland		Grazing land	
	%	Area (ha)	Area (%)	Area (ha)	Area (%)	Area (ha)	Area (%)
Low	0.1-0.2	1,219.37	27.95	69.66	16.29	115.14	19.62
Medium	0.2-0.3	2,159.42	49.50	62.31	14.57	244.65	41.68
high	0.3-0.4	983.94	22.55	295.63	69.14	227.18	38.70
Total		4,362.73	100	427.60	100	586.98	100
Mean TN (%)		0.17^{a}		0.27^{b}		0.35^{c}	

Means of TN with different letters are significantly different (Tukey, $p < 0.01$).

Exchangeable cations (K, Ca) and electrical conductivity (EC)

As shown in Tables 5-6 and Figs 5-6, there was a significant variation in soil K, Ca, and EC contents based on land-use. Besides, according to [59, 61], the soils in the study region had high Ca (>20 $cmol_{(+)}$ kg^{-1}) and K (0.51-1.51 $cmol_{(+)}$ kg^{-1}) contents. In the soils, higher Ca and K levels were found. It might be due to the type of parent materials, weathering, land-use types, fertilizer types and leaching rates, crop remains, and litterfall [71]. The higher Ca and K contents present in grazing and plantation forestlands are associated with the higher pH value [72] and clay particles [73]. On the other hand, the soils showed slightly salty.

This result suggests that Ca and K do not appear to be limiting nutrients to crop production in the region. Based on the EC rate established by [74], no significant amounts of soluble salts were accumulated, implying that plant growth and development would be unaffected.

Fig 6. Maps: (r) Exchangeable calcium, (s) Exchangeable potassium, (t) Electrical conductivity, and (u) Cation exchange capacity.

Table 5. Soil exchangeable potassium (exchK) rating and its area share (ha, %) by land-use and its parametric test.

Common name	ExchK (rate)	Area share (ha, %) by land-use					
		Cropland		Plantation forestland		Grazing land	
	$cmol_{(+)}\ kg^{-1}$	Area (ha)	Area (%)	Area (%)	Area (ha)	Area (%)	Area (ha)
High	0.51-1.51	2,609.17	59.81	320.17	45.98	257.26	43.83
Medium	1.51-2.3	1,733.37	39.73	373.31	53.61	326.57	55.64
Very high	>2.3	20.19	0.46	2.80	0.40	3.15	0.54
Total		4,362.73	100	696.29	100	586.98	100
Mean exchK ($cmol_{(+)}\ kg^{-1}$)		0.87^{a}		1.47^{b}		1.81^{c}	

Means of exchK with different letters are significantly different (Tukey, $p < 0.01$).

Table 6. Electrical conductivity (EC) rating and its area share (ha, %) by land-use and its parametric test.

Common name	EC Rate	Area share (ha, %) by land-use					
		Cropland		Plantation forestland		Grazing land	
	$dS\ m^{-1}$	Area (ha)	Area (%)	Area (ha)	Area (%)	Area (ha)	Area (%)
Not salty	<0.75	1,427.14	32.71	96.07	13.80	146.18	24.90
Slightly salty	0.75-2	2,660.19	60.98	538.15	77.29	372.90	63.53
Moderately salty	2-4	275.30	6.31	62.07	8.91	67.90	11.57
Sum		4,362.63	100	696.29	100	586.98	100
Mean EC ($dS\ m^{-1}$)		0.13^{a}		1.21^{b}		2.22^{c}	

Means of EC with different letters are significantly different (Tukey, $p < 0.01$).

Cation Exchange Capacity (CEC)

According to the rate set by [61], a significant variance and higher CEC indicated that soils in the study area have a high capacity to retain nutrients against leaching losses (Table 7 and Fig 6). The highest CEC values recorded in cultivated land-use may be due to soil organic material, pH, quantity, and type of clay, which adsorb and retain positive cations through electrostatic force [75]. The current findings were also consistent with [76-78], who reported higher CEC under cultivated lands in Ethiopia's highlands.

Table 7. Cation exchange capacity (CEC) rating and its area share (ha, %) by land-use and its parametric test.

Common name	CEC (rate)	Area share (ha, %) by land-use					
		Cultivated land		Plantation forestland		Grazing land	
	$cmol_{(+)}\ kg^{-1}$	Area (ha)	Area (%)	Area (ha)	Area (%)	Area (ha)	Area (%)
High	15-30	1,402.31	32.14	357.07	51.28	275.72	46.97
Very high	>30	2,960.42	67.86	339.22	48.72	311.26	53.03
Sum		4,362.73	100	696.29	100	586.98	100
Mean CEC		40.36^{a}		31.53^{b}		25.24^{c}	

Means of CEC with different letters are significantly different (Tukey, $p < 0.01$).

Principal component analysis (PCA) of soil chemical properties

The two principal component analyses (PCA) with eigenvalues greater than one were able to explain the most significant variance (98.55%) of the analyzed soil chemical properties (S1 Table) [79]. Moreover, 79.97 % of the variation in data was explained by pH, organic carbon (OC), total nitrogen (TN), exchangeable potassium (exchK), electrical conductivity (EC), and cation exchangeable capacity (CEC) on the first PC. Simultaneously, the second component notably loaded the available phosphorus (avP) and exchangeable

calcium (exchCa) (Table 8 and S1 Fig). As a result, the PCA reduces the dimensions and complexity of the soil data matrix [80].

Table 8. Principal component analysis of soil chemical properties about land-uses in Fogera plain, northwest Ethiopia.

**Principal component**	**PC1**	**PC2**
Eigenvalue	6.07	1.29
Variance (%)	79.97	18.58
**Variables**	**Eigenvectors**	
pH	**0.96**	0.12
Available phosphorus	0.48	**0.86**
Organic carbon	**0.96**	0.03
Total nitrogen	**0.96**	0.00
Exchangeable potassium	**0.81**	-0.08
Exchangeable calcium	-0.67	**0.73**
Electrical conductivity	**0.99**	0.03
Cation exchangeable capacity	**-1.00**	0.04

Bold eigenvector values referred to highly weighted variables in the PC.

Implications for sustainable soil fertility management and environmental conservation

Our results showed that the transition from grassland to cultivated land and eucalyptus plantation results in a significant reduction in the total nitrogen within Fogera plain's topsoil. Organic materials may be used to reduce nitrogen depletion. Nevertheless, avP content of the area’s soils was high (>20 ppm), which could be attributed to the frequent use of mineralized phosphorus [38]. The addition of nitrogen-containing fertilizer inputs might also improve the cultivated lands' soil nutrient supply to better crop yield and farming

profitability. However, the overuse of nitrogen and phosphorus fertilizer may lead to global climate change due to their energy-intensive processing and inefficient use [81], eutrophication of water bodies [82], and soil acidification [83]. Besides, total greenhouse gas emissions, including N_2O, CO_2 have increased under cultivated lands, depending on the decomposition of organic materials in the soil [81]. Soil management methods, optimum N application rate [84], and organic resources and nitrification inhibitors are all possible soil management approaches.

The information of spatial soil variability across land-uses is vital for sustainable land management practices, which could reduce soil erosion, enhance land productivity, improve farmers' livelihood, reduce GHGs, and maintain environmental quality [85-86]. Furthermore, we should develop relevant land-use planning and policies to provide an optimal solution geared toward improving the soil's nutrient use efficiency and reducing the adverse environmental effects, including nitrate losses to water and N_2O emissions [87-88].

Conclusion

The majority of soil properties across various land-uses were normally distributed (S2 Fig). We obtained a substantial difference in soil pH, available phosphorus (avP), organic carbon (OC), total nitrogen (TN), electrical conductivity (EC), exchangeable bases (Ca and K), and cation exchange capacity (CEC) over land-uses. Under these conditions, grazing land had the highest mean values of pH (8.44), avP (31.51 ppm), OC (3.487 %), TN (0.313 %), EC (2.22 dS m^{-1}), and exchK (3.6 $cmol_{(+)}$ kg^{-1}), while in cultivated land had the lowest OC (2.44 %), TN (0.17 %), soil pH (6.58), exchK (0.87 $cmol_{(+)}$ kg^{-1}), and EC (0.13 dS m^{-1}). Besides, PCA explained 98.55% of the soil variability because of land-use change. The expansion of cultivated lands depleted soil OC and TN, restricting crop growth and decreasing crop yield.

Thus, soil management alternatives should gear to replenish the OC and N contents of the soil for increasing crop yield in the area. The interpolated maps of soil properties help farmers and decision-makers in improve soil management alternatives, optimize fertilizer use, enhance crop productivity, and reducing GHGs emissions. As small soil samples limited the study, many models on the larger scale should gear in future studies to understand better the spatial variability of soil properties of the Fogera plain, Ethiopia.

Acknowledgment

Our grateful acknowledgment directs to Debre Tabor University and Bahir Dar University for the financial grant. We substantially recognized the Amhara Regional Soil Laboratory Center for laboratory analysis for this study. Besides, we acknowledged the Water and Land Resource Centre (WLRC) and Ethiopian Mapping Agency (EMA) for various data access.

Author contributions

Gizachew Ayalew designed the method and wrote the draft manuscript. Tiringo Yilak and Derege Tsegaye Meshesha have been accountable for data analysis and interpretation of the results. Eduardo Saldanha Vogelmann, José Miguel Reichert, and Nigussie Haregeweyn analyzed/interpreted the results, reviewed/commented on the manuscript.

Declaration of conflicting interests

The authors declare have no conflicting interests.

References

1. Burst M, Chauchard S, Dambrine E, Dupouey JL, Amiaud B. Distribution of soil properties and forest-grassland interfaces: influence of permanent environmental factors or land-use after-effects. Agric Ecosyst Environ. 2020; 289:106739. https://doi.org /10.1016/j.agee.2019.106 739.
2. Fentie SF, Jembere K, Fekadu E, Wasie D. Land-use and land-cover dynamics and soil properties under different land-uses in the tejibara watershed, Ethiopia. Sci World JP. 2020; 1-12. https://doi.org/10.1155/2020/1479460.
3. Saha D, Kukal SS. Soil Structural Stability and Water Retention Characteristics under Different Land-uses of Degraded Lower Himalayas of North-West India. Land Degrad Dev. 2013; 26(3): 263-271. doi:10.1002/ldr.2204.
4. Reichert JM, Rodrigues MF, Bervald CMP, Brunetto G, Kato OR, Schumacher MV. Fragmentation, fiber separation, decomposition, and nutrient release of secondary-forest biomass, mechanically chopped-and-mulched, and cassava production in the Amazon. Agric Ecosyst Environ. 2015; 204: 8-16. https://doi.org/10.1016/j.agee.2015.02.005.
5. Reichert JM, Rodrigues MF, Bervald CMP, Kato OR. Fire-free fallow management by mechanized chopping of biomass for sustainable agriculture in eastern Amazon: effects on soil compactness, porosity, and water retention and availability. Land Degrad Dev. 2016a; 27: 1403–1412. https://doi.org/10.1002/ldr.2395.
6. Bufebo B, Elias E. Effects of Land-Use/Land-Cover Changes on Selected Soil Physical and Chemical Properties in Shenkolla Watershed, South Central Ethiopia. Advances in Agriculture. 2020. https://doi.org/10.1155/2020/5145483.
7. Barua SK, Haque SMS. Soil characteristics and carbon sequestration potentials of vegetation in degraded hills of Chittagong, Bangladesh. Land Degrad Dev. 2013; 24: 63-71. https://doi.org/10.1002/ldr.1107.

8. Suzuki LEAS, Reichert JM, Reinert DJ, Lima CLR de. Degree of compactness and mechanical properties of a subtropical Alfisol with eucalyptus, native forest, and grazed pasture. Forest Sci. 2015; 61:716-722. https://doi.org/10.5849/forsci.14-172.
9. Reichert JM, Amado TJC, Reinert DJ, Rodrigues MF, Suzuki LEAS. Land-use effects on subtropical, sandy soil under sandyzation/desertification processes. Agric Ecosyst Environ. 2016b; 233: 370-380. https://doi.org/10.1016/j.agee.2016.09.039.
10. Reichert JM, Cechin NF, Reinert DJ, Rodrigues MF, Suzuki LEAS. Ground-based harvesting operations of Pinus taeda affects the structure and pore functioning of clay and sandy clay soils. Geoderma. 2018; 331:38–49. https://doi.org/10.1016/j.geoderma.2018.06.012.
11. Holthusen D, Brandt AA, Reichert JM, Horn R. Soil porosity, permeability, and static and dynamic strength parameters under native forest/grassland compared to no-tillage cropping. Soil and Till Res. 2018a; 177:113–124. https://doi.org/10.1016/j.still.2017.12.003.
12. Holthusen D, Brandt AA, Reichert JM, Horn R, Fleige H, Zink A. Soil functions and in situ stress distribution in subtropical soils as affected by land-use, vehicle type, tire inflation pressure, and plant residue removal. Soil and Till Res. 2018b; 184:78–92. https://doi.org/10.1016/ j.still.2018.07.009.
13. Tesfahunegn GB. Soil quality indicators response to land-use and soil management systems in Northern Ethiopia's catchment. Land Degrad Dev. 2016; 27: 438–448. https://doi.org/10.1002/ldr.2245, 2016.
14. Biro K, Pradhan B., Muchroithner M, Makeschin F. Land-use/land-cover change analysis and its impact on soil properties in the northern part of Gadarif region, Sudan, Land Degrad Dev. 2013; 24: 90–102. https://doi.org/10.1002/ldr.1116.

15. Asmamaw LB, Mohammed AA. Effects of slope gradient and land-use/land-cover on selected soil physical and biochemical properties of the Gerado catchment, North-eastern Ethiopia. Int J Environ Stud. 2013; 70: 111–125. https://doi.org/10.1080/00207233.2012.751167.

16. Emiru N, Gebrekidan H. Effect of land-use changes and soil depth on soil organic matter, total nitrogen and available phosphorus contents of soils in Senbat watershed, western Ethiopia. J Agric Biol Sci. 2013; 8: 206–212.

17. Stoorvogel JJ, Smaling EMA. Assessment of soil nutrient depletion in Sub-Saharan Africa: 1983-2000. Vol. 2: Nutrient balances per crop and land-use systems (Report/ Winand Staring Centre; No. 28). ISRIC, 1990. https://edepot.wur.nl/3 05176 19.

18. Stoorvogel JJ, Smaling EM, Janssen BH. Calculating soil nutrient balances in Africa at different scales. Fertilizer res. 1993; *35*(3): 227-235.

19. Tadele D, Teketay D. Growth and yield of two-grain crops on sites former covered with eucalypt plantations in Koga Watershed, northwestern Ethiopia. J For Res. 2014; 25: 935–940. https://doi.org/10.1007/s11676-014-0483-9.

20. Jaleta D, Mbilinyi B, Mahoo H, Lemenih M. Eucalyptus expansion as relieving and provocative tree in Ethiopia. J Agric Ecol Res Int. 2016a; 6(3): 1-12. https://doi.org/10.9734/JAERI/2016/228 41.

21. Jaleta D, Mbilinyi B, Mahoo H, Lemenih M. Evaluation of land-use/land-cover changes and eucalyptus expansion in Meja watershed, Ethiopia. J Geo Environ Earth Sci Int. 2016b; 7(3): 1-12. https://doi.org/10.9734/JGEESI/2016/26606.

22. Fantaw Y, Abdu A. Soil property changes following the conversion of acacia woodland into grazing and farmlands in Ethiopia's rift valley area. Land Degrad Dev. 2011; 22:425–431. https://doi.org/10.1002/ldr.1022.

23. Assefa F, Elias E, Soromessa T, Ayele GT. Effect of changes in land-use management practices on soil physical and chemical properties in Kabe Watershed, Ethiopia. Air, Soil, and Water Res. 2020; 13: 1-16. https://doi.org/10.1177/1178622120939587.

24. Valentini R, Arneth A, Bombelli A, Castaldi S, Cazzolla Gatti R, Chevallier F, et al. A full greenhouse gases budget of Africa: synthesis, uncertainties, and vulnerabilities. Biogeosciences. 2014; 11(2):381-407. https://doi.org/10.5194/bg-11-381-2014.

25. Allen DE, Kingston G, Rennenberg H, Dalal RC, Schmidt S. Effect of nitrogen fertilizer management and waterlogging on nitrous oxide emission from subtropical sugarcane soils. Agric Ecosyst Environ. 2010; 136 (3-4): 209-217. https://doi.org/10.1016/j.agee. 2009.11.002.

26. Venterea RT, Halvorson AD, Kitchen N, Liebig MA, Cavigelli MA, Del Grosso SJ, et al. Challenges and opportunities for mitigating nitrous oxide emissions from fertilized cropping systems. Front Ecol Environ. 2012; 10(10): 562-570. https://doi.org/10.1890/12 0062.

27. Hickman JE, Tully KL, Groffman PM, Diru W, Palm CA. A potential tipping point in tropical agriculture: Avoiding rapid increases in nitrous oxide fluxes from agricultural intensification in Kenya. J Geophys Res Biogeosci. 2015; 120(5): 938-951. https://doi. org/10.1002/2015JG002913.

28. Qiu SJ, Ju XT, Ingwersen J, Qin ZC, Li L, Streck T, et al. Changes in soil carbon and nitrogen pools after shifting from conventional cereal to greenhouse vegetable production. Soil and Till Res. 2010; 107(2): 80-87. https://doi.org/10.1016/ j.still.2010.02.006.

29. Tesfahunegn GB, Tamene L, Vlek Paul LG. Catchment-scale spatial variability of soil properties and implications on site-specific soil management in northern Ethiopia. Soil and Till Res. 2011; 117:124–139. doi.org/10.1016/j.still.2011.09.005.

30. Yasrebi J, Saffari M, Fathi H, Karimian N, Emadi M, Baghernejad M. Spatial variability of soil fertility properties for precision agriculture in Southern Iran. J. Appl. Sci. 2008 8:1642-1650. https://doi: 10.3923/jas.2008.1642.1650.

31. Appel Neto E, Seidel EJ, Oliveira MS. Geostatistical-based index for spatial variability in soil properties. Rev Bras Cienc Solo. 2020; 44: e0200086. https://doi.org/10.36783/1806 967r bcs20200086.

32. Ichami SM, Shepherd KD, Hoffland E, Karuku GN, Stoorvogel JJ. Soil spatial variation to guide the development of fertilizer use recommendations for smallholder farms in western Kenya. Geoderma Reg. 2020; p.e00300. https://doi.org/10.1016/j.geodrs.2020. e00300.

33. Jurišić M, Radočaj D, Krčmar S, Plaščak I, Gašparović M. Geostatistical Analysis of Soil C/N Deficiency and Its Effect on Agricultural Land Management of Major Crops in Eastern Croatia. Agronomy. 2020; 10(12): 1996. https://doi.org/10.3390/agronomy1012 1996.

34. Macedo Neto AAL, Farias PRS, Matos GSBD, Silva GBD, Santos AVFD, Anhê BB. Diagnosis and spatial variability of soil fertility and crop production in a teak area in eastern Pará State Cerne. 2020; 26(1): 37-47.https://doi.org/10.1590/01047760202026012 683.

35. Burgess TM, Webster R. Optimal interpolation and isar1thmic mapping of soil properties, Eur J Soil Sci. 1980; 31:315-331. https://doi.org/10.1111/ejss.12749.

36. Guan F, Xia M, Tang X, Fan S. Spatial variability of soil nitrogen, phosphorus, and potassium contents in Moso bamboo forests in Yong’an City, China. Catena. 2017; 150: 161–72. https://doi.org/10.1016/j.catena.201.11.017.

37. Rosemary F, Vitharana UWA, Indraratne SP, Weerasooriya R, Mishra U. Exploring the spatial variability of soil properties in an Alfisol soil catena. Catena. 2017; 150: 53–61. https://doi.org/10.1016/j.catena.2016.10.017.
38. Panday D, Maharjan B, Chalise D, Shrestha RK, Twanabasu B. Digital soil mapping in the Bara District of Nepal using Kriging tool in ArcGIS. PLoS ONE. 2018; 13(10):e020 6350. https://doi. org/10.1371/journal.pone.0206350.
39. Behera SK, Shukla AK. Spatial distribution of surface soil acidity, electrical conductivity, soil organic carbon content, and exchangeable potassium, calcium, and magnesium in some cropped acid Soils of India. Land Degrad Dev. 2015; 26: 71–79. https://doi.org/10.1 002/ldr.2306.
40. Ethiopian Mapping Agency, P.O.Box 597, Addis Abeba. [Accessed 2019 January 18]. http://www.ema.gov.et/.
41. Water and Land Research Center (WLRC). National Geospatial database System EthioGIS-2/Release. 2014.
42. Buttafuoco G, Castrignanò A, Cucci G, Lacolla G, Lucà F. Geostatistical modelling of within-field soil and yield variability for management zones delineation: A case study in a durum wheat field. Precis. Agric. 2017; 18:37-58.
43. Reza SK, Baruah U, Sarkar D, Singh SK. Spatial variability of soil properties using geostatistical method: a case study of lower Brahmaputra plains, India. Arab. J. Geosci. 2016; 9(6): 446. https://doi:10.1080/03650340.2017.1296134.
44. Özyazici MA, Dengiz O, Sağlam M, Erkoçak A, Türkmen F. Mapping and assessment-based modeling of soil fertility differences in the central and eastern parts of the Black Sea region using GIS and geostatistical approaches. Arab. J. Geosci. 2017; 10(2): 45.

45. Bekele T, Sertsu S. Procedures for soil and plant analysis. National Soil Research Centre. Ethiopian Agricultural Research Organization Technical paper No. 74, 2000. Addis Ababa, Ethiopia.
46. Van Reeuwijk LP. Procedure for soil analysis. 4th ed. International soil reference center Wageningen. (ISRIC) Technical Paper No. 9, 1993. The Netherlands, Wageningen.
47. USDA Soil Survey Laboratory manual, Soil Survey Investigation Report no. 42, version 4, USDA-NRCS, Nebraska, USDA. 2004.
48. Walkley A, Black CA. An examination of Degtjareff methods for determining soil organic matter and the proposed modifications of the chromic acid titration method. Soil Sci. 1934; 37: 29–38. https://doi.org/10.1097/00010694-193401000-00003.
49. Bremmer JM, Mulvaney CS. Nitrogen total. In: Page, A.L. (Ed.), Method of Soil Analysis. Part 2. Chemical and Microbiological Properties. Am. Soc. Agron., WI, Agron. Monogr. 1982; 9: 595–624. doi.org/10.2134/agronmonogr9.2.2ed.c31.
50. Olsen SR, Cole CV, Watanabe FS, Dean LA. Estimation of available phosphorus in soils by extraction with sodium bicarbonate. United States Department of Agriculture Circular. Washington, DC; 1954; p. 939. https://doi.org/10.1002/jsfa.2740660423.
51. Jackson ML. Soil chemical analysis. New Delhi, India: Prentice- Hall of India Private Ltd.. 1967.https://doi.org/10.1002/jpln.19590850311.
52. Rowell DL. Soil science: Methods and application, Addison Wesley Longman, Limited, England. 1994.
53. Chapman HD. Cation exchange capacity. In C.A. Black (Ed.). Methods of soil analysis – Chemical and microbiological properties, Madison, WI: J Am Soc Agron. 1965; 9: 891–901. https://doi.org/10.2134/agronmonogr9.2.c6.

54. Venteris E, Basta N, Bigham J, Rea R. Modeling Spatial Patterns in Soil Arsenic to Estimate Natural Baseline Concentrations. J. Environ. Qual. 2014; 43: 936-946. https:// doi.org/10.2134/jeq2013.11.0459.
55. Tripathi R, Nayak AK, Shahid M, Raja R, Panda BB, Mohanty S, et al. Characterizing Spatial Variability of Soil Properties in Salt Affected Coastal India Using Geostatistics and Kriging. Arab. J. Geosci. 2015; 8: 10693-10703. https://doi.org/10. 1007/s12517-015-2003-4.
56. Gotway CA, Ferguson RB, Hergert GW, Peterson TA. Comparison of kriging and inverse-distance methods for mapping soil parameters. Am J Soil Sci. 1996; 60:1237–1247. https://doi.org/10.2136/sssaj1996.03615995006000040040x.
57. Wollenhaupt NC, Wolkowski RP, Clayton MK. Mapping soil test phosphorus and potassium for variable-rate fertilizer application. J Prod Agric. 1994; 7(4): 441-448. https: //doi.org/10.2134/jpa 1994.0441.
58. Robinson TP, Metternicht G. Comparing the performance of techniques to improve the quality of yield maps. Agric Syst. 2005; 85(1):19-41. https://doi.org/10.1016/j.agsy.2004. 07.010.
59. EthioSIS (Ethiopia Soil Information System). Soil fertility status and fertilizer recommendation atlas for Tigray regional state, Ethiopia. Addis Ababa, Ethiopia: Agricultural Transformation Agency. 2014; 76 pp.
60. Moraes JFL, Volkoff B, Cerri CC, Bernoux M. Soil properties under Amazon forest and changes due to pasture installation in Rondonia. Brazil. Geoderma. 1996; 70 (1): 63–81. https://doi.org/10.1016/0016-7061(95)00072-0.
61. Hazelton P, Murphy B. Interpreting soil test results: what do all the numbers mean? Australia: CSIRO Publishing, Collingwood Victoria–Australia. http://www.publish. CSIRO. 2007; 160 p. https://doi.org/10.1111/sum.12402.

62. Miheretu BA, Yimer AA. Spatial variability of selected soil properties to land-use and slope position in Gelana sub-watershed, Ethiopia's northern highlands. Phys Geogr. 2018; 39 (3): 230–245. https://doi.org/10.1080/02723646.2017.1380972.
63. Assefa A, van Keulen H. Modeling soil nutrient dynamics under alternative farm management practices in the Northern Highlands of Ethiopia. Soil and Till Res. 2009; 103:203–215. https://doi.org/10.1016/j.still.2008.05.022.
64. Moges A, Dagnachew M, Yimer F. Land-use effects on soil quality indicators: a case study of Abo-Wonsho Southern Ethiopia. Appl Environ Soil Sci. 2013. https://doi.org/10.1155/2013/784989.
65. Food and Agriculture Organization (FAO). Soil and plant testing as a basis of fertilizer recommendations. Soils Bulletin No. 38/2. Rome. 1980; 100 pp.
66. Tellen VA, Yerima BPK. Effects of land-use change on soil physical and chemical properties in selected areas in Cameroon's North West region. Environ Syst Res. 2018; 7:3. https://doi.org/10.1186/s40068-018-0106-0.
67. Juo ASR, Manu A. Chemical dynamics in slash-and-burn agriculture. Agric Ecosyst Environ. 1996; 58:49–60. https://doi.org/10.1016/0167-8809 (95)00656-7.
68. Kassa Cholbe M, Kebede Yeme F, Haile Woldeyohannes W. Fertility Status of Acid Soils under Different Land-Use Types in Wolaita Zone, Southern Ethiopia. Appl Environ Soil Sci. 2020. https://doi.org/10.1155/2020/3713967.
69. McNaughton SJ, Banyikwa FF, McNaughton MM. Promotion of the cycling of diet-enhancing nutrients by African grazers. Science. 1997; 278: 1798–1800. https://doi.org/10.1126/ science.278.5344.1798.
70. Braimoh AK, Vlak PL. The impact of land-cover change on soil properties in northern Ghana. Land Degrad Dev. 2004; 15: 65–74. https://doi.org/10.1002/ldr.590.

71. Uzoho BU, C Ekeh. Potassium status of soils in relation to land use types in Ngor-Okpala, South-eastern Nigeria. J. Nat. Sci. Res. 2014; 4 (6):105–14.
72. Mesfin Abebe. The challenges and prospects of soil chemistry in Ethiopia. In: Teshome Yizengaw, Eyasu Mekonnen, and Mintesinot Behailu, editors. Proceedings of the 3rd Conference of the Ethiopian Society of Soil Science (ESSS). Addis Ababa, Ethiopia. Ethiopian Science and Technology Commission; 1996; p. 78-96.
73. Terefe H, Argaw M, Tamene L, Mekonnen K, Recha J, Solomon D. Effects of sustainable land management interventions on selected soil properties in Geda watershed, central highlands of Ethiopia. Ecol Process. 2020; 9(1): 1-11. https://doi.org/10.1186/s13 717-020-02 16- 2.
74. Jahn R, Blume HP, Asio VB, Spaargaren O, Schad P. Guidelines for soil description. 4th ed**.** Rome, FAO. 2006; 97 pp.
75. Gol C, Cakir M, Edis S, Yilmaz H. The effects of land-use/land-cover change and demographic processes (1950–2008) on soil properties in the Gokcay catchment, Turkey. Afr J Agric Res. 2010; 4:1670–1677. https://doi.org/10.5897/AJAR09.716.
76. Kokeb A, Muche M, Molla E. Assessing the Physical and Chemical Properties of Soil under Different Land-Use Types. J Environ Anal Toxicol. 2015; 5(5):309. https://doi.org/10.4172/2161-0525.1000309.
77. Selassie YG, Anemut F, Addisu S. The effects of land-use types, management practices and slope classes on selected soil physical and chemical properties in Zikre watershed, North-Western Ethiopia. Environ Syst Res. 2015; 4:3. https://doi.org/10.1186/s40068-015-0027-0.
78. Molla FE, Yalew AM. The effects of land-use types and soil depth on soil properties of Agedit watershed, Northwest Ethiopia. Ethiop J Sci & Technol. 2018; 11(1): 39-56. https://doi.org/10.4314/ejst.v11i1.4.

79. Gniazdowski Z. New interpretation of principal components analysis. Zesz Nauk WWSI. 2017; 11:43-65. https://doi.org/10.26348/znwwsi.16.43.

80. Jolliffe IT, Cadima J. Principal component analysis: a review and recent developments, Philos Trans A Math Phys Eng Sci. 2016; 374(2065):20150202. Epub 2016/03/10. https://doi.org/10.1098/rsta.2015.0202.

81. Tubiello FN, Salvatore M, Condor Golec R, Rossi S, Ferrara A, Biancalani R, et al. The contribution of agriculture, forestry, and other land-use activities to global warming, 1990 –2012. Glob Change Biol. 2015; 21(7): 2655–2660. https://doi:10.1111/gcb.12865.

82. Galloway JN, Townsend AR, Erisman JW, Bekunda M, Cai Z, Freney JR, et al. Transformation of the nitrogen cycle: recent trends, questions, and potential solutions. Science. 2008; 320: 889–92. https://doi:10.1126/science.1136674.

83. Tian HQ, Lu CQ, Melillo J, Ren R, HuangY, Xu XF, et al. Food benefit and climate warming potential of nitrogen fertilizer uses in China. Environ Res Lett. 2012; 7. https://doi:10.1088/1748-9 326/7/4/044020.

84. Millar N, Robertso GP, Grace PR, Gehl RJ, Hoben JP. Nitrogen fertilizer management for nitrous oxide (N_2O) mitigation in intensive corn (Maize) production: an emissions reduction protocol for US Midwest agriculture. Mitig Adapt Strateg Glob Chang. 2010; 15(2):185-204. https://doi 10.1007/s11027-010-9212-7.

85. Food and Agriculture Organization (FAO). Integrated soil management for sustainable agriculture and food security in Southern and East Africa, FAO, Rome, Italy. 1999.

86. Novara A, Gristina L, Guaitoli F, Santoro A, Cerdà A. Managing soil nitrate with cover crops and buffer strips in Sicilian vineyards. Solid Earth. 2013; 4(2): 255–262. https:// doi.org/10.5194/se-4-255-2013.

87. Venterea RT, Maharjan B, Dolan MS. Fertilizer source and tillage effects on yield- scaled nitrous oxide emissions in a corn cropping system. J. Environ. Qual. 2011; 40(5): 1521-1531. https://doi:10.2134/jeq2011.0039.

88. Snyder CS, Davidson EA, Smith P, Venterea RT. Agriculture: sustainable crop and animal production to help mitigate nitrous oxide emissions. Curr Opin Environ Sustain. 2014; 9-10: 46-54. https://doi.org/10.1016/j.cosust.2014.07.005.

Supporting information

S1 Table. Laboratory results of soil chemical properties in Fogera plain

S1 Fig. Loading plot: Organic carbon (OC), Total nitrogen (TN) Available phosphorus (avP), exchangeable calcium (ExchCa), Exchangeable potassium (ExchK), Cation exchange capacity (CEC), and Electrical conductivity (EC).

S2 Fig. Q-Q plots of soil fertility parameters in Fogera plain.

Figure

Click here to access/download;Figure;Fig 1.tif

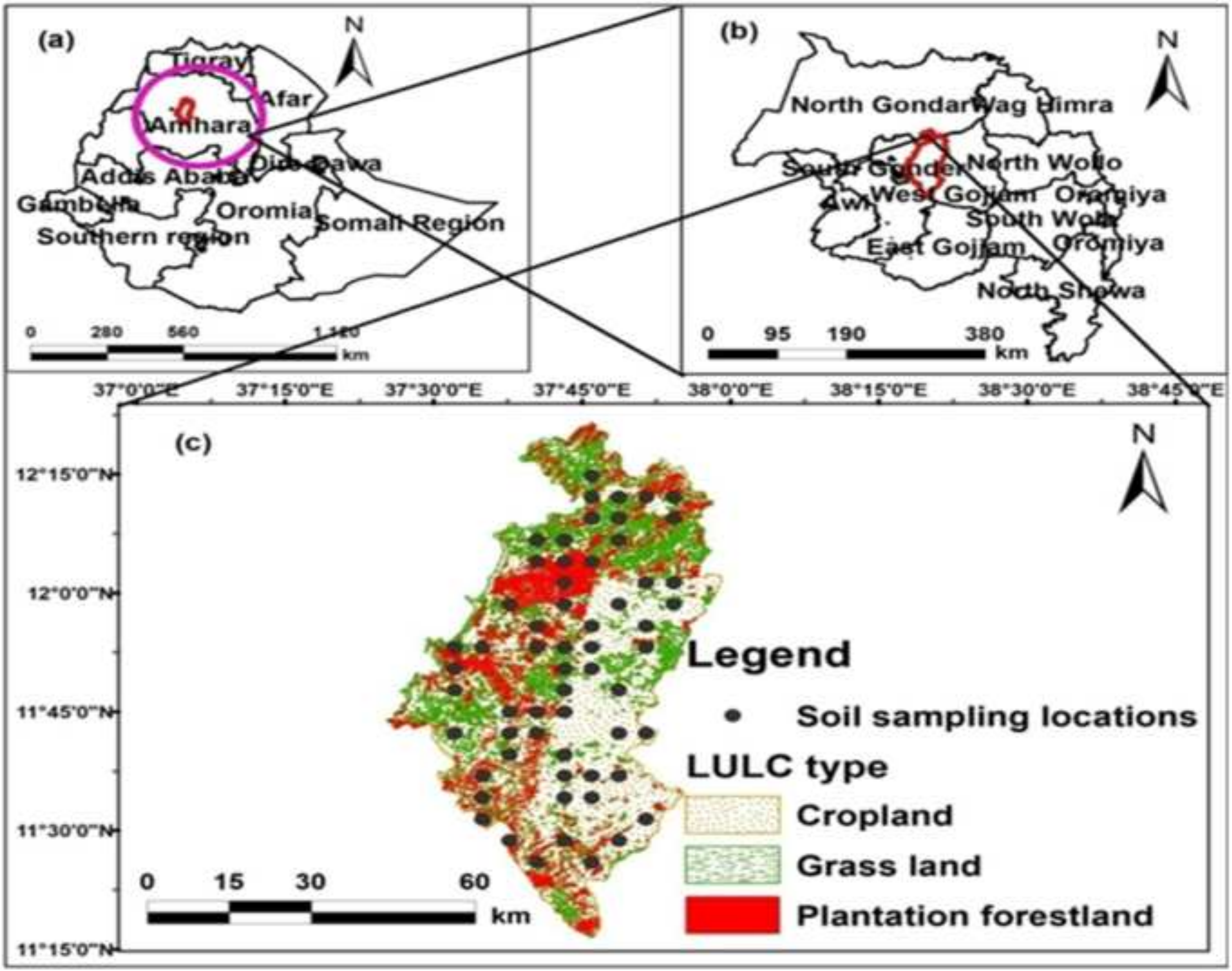

Click here to access/download;Figure;Fig 2.tif

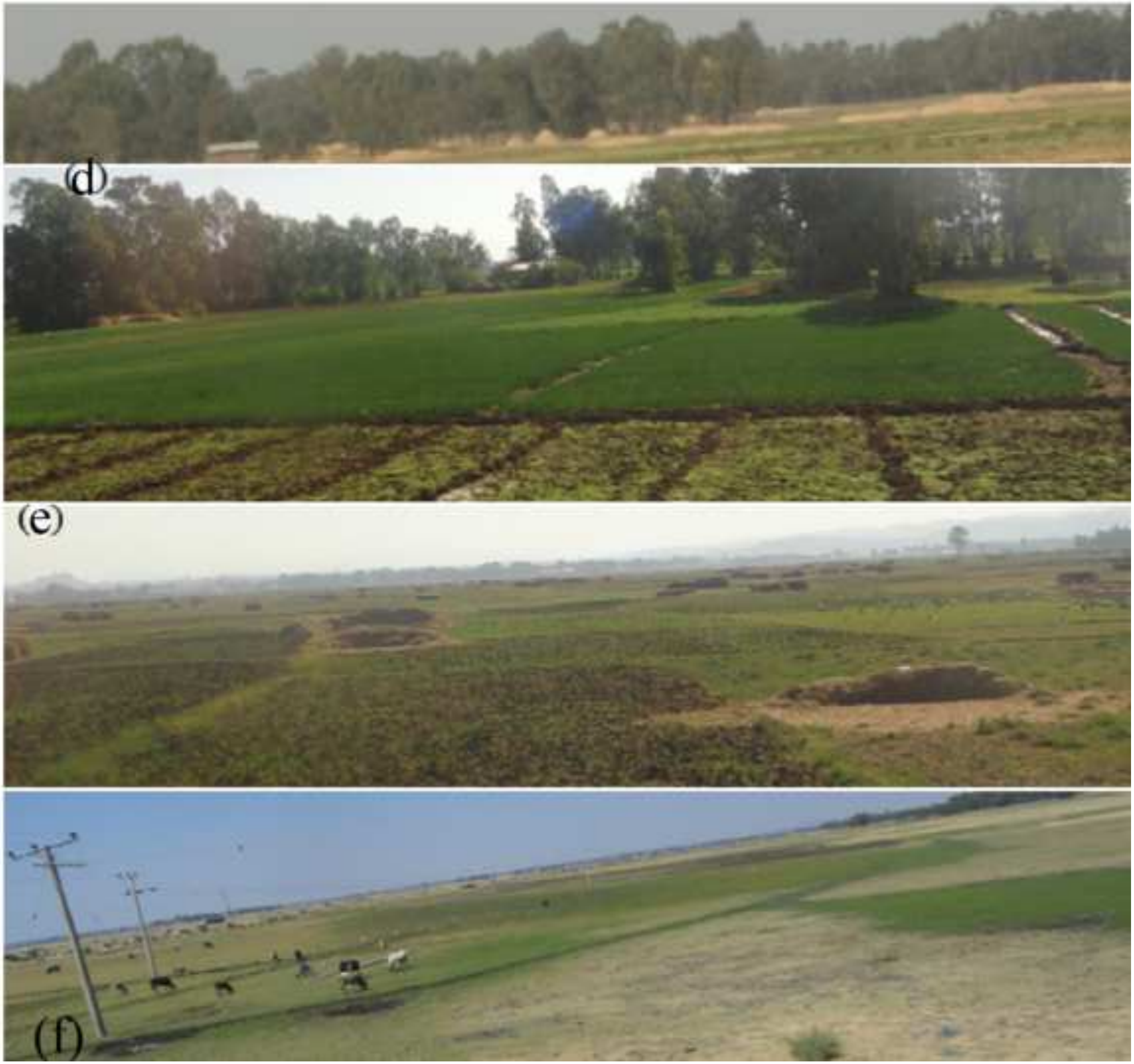

Figure Click here to access/download;Figure;Fig 3.tif

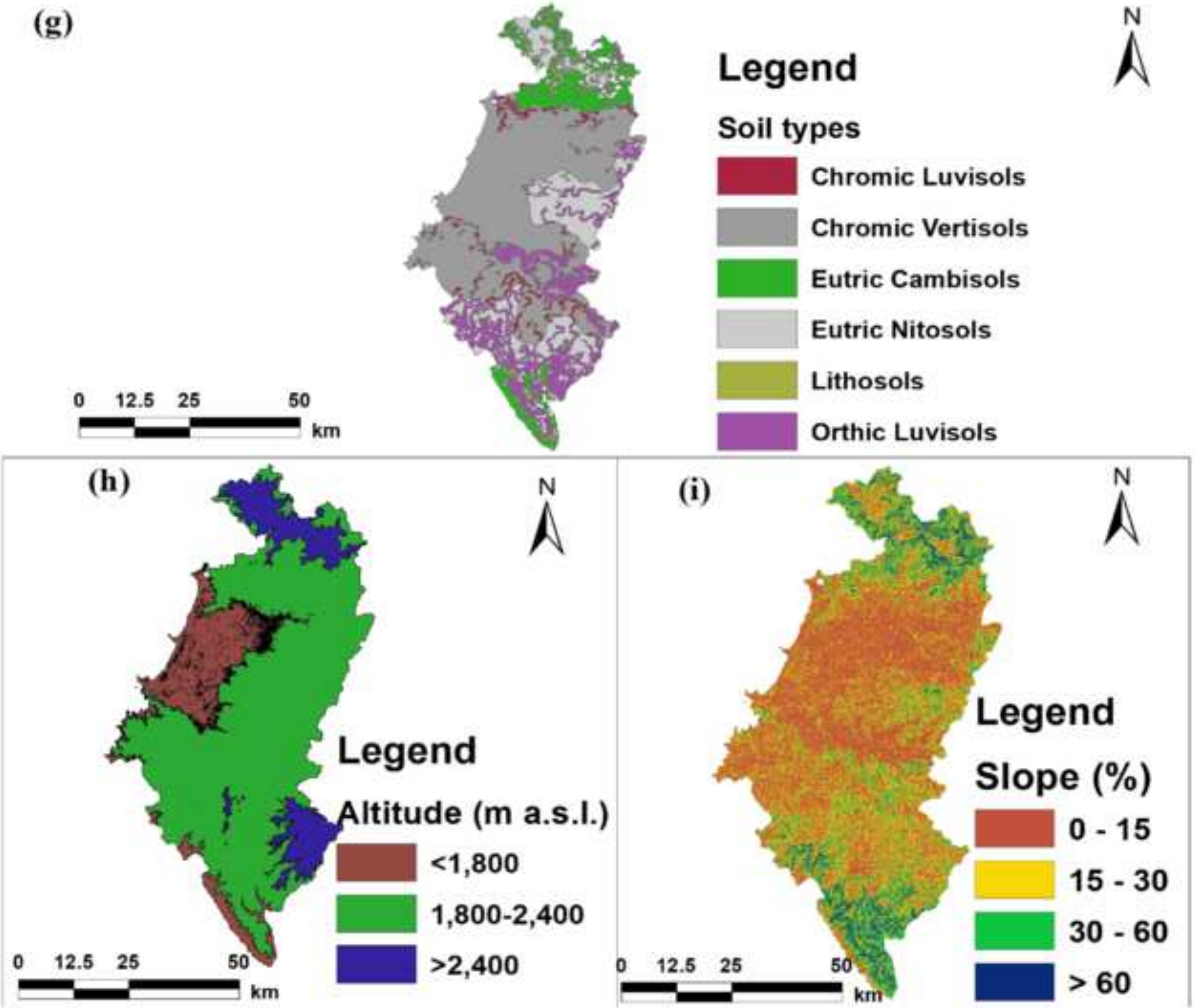

Click here to access/download;Figure;Fig 4.tif

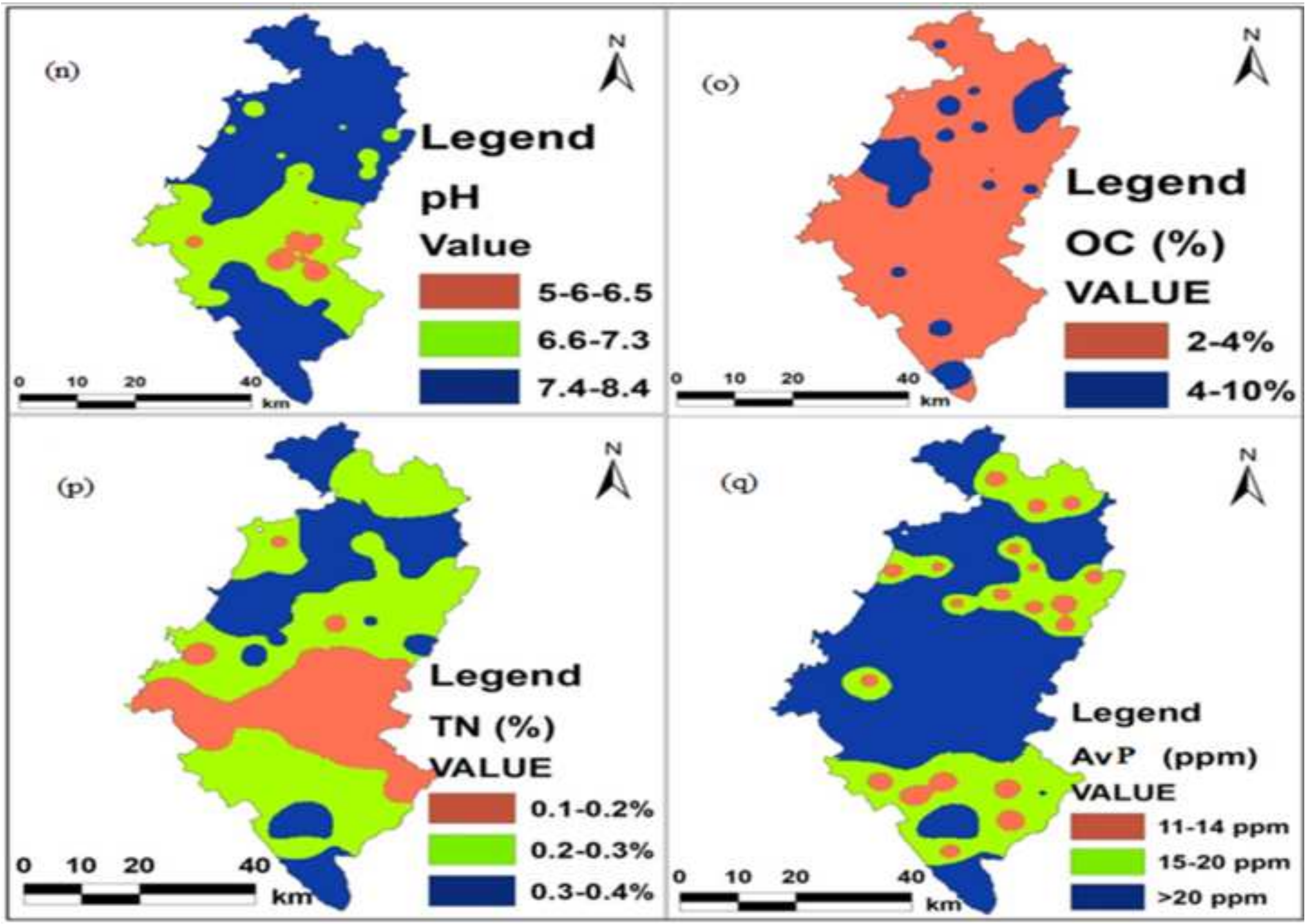

Figure

Click here to access/download;Figure;Fig 5.tif

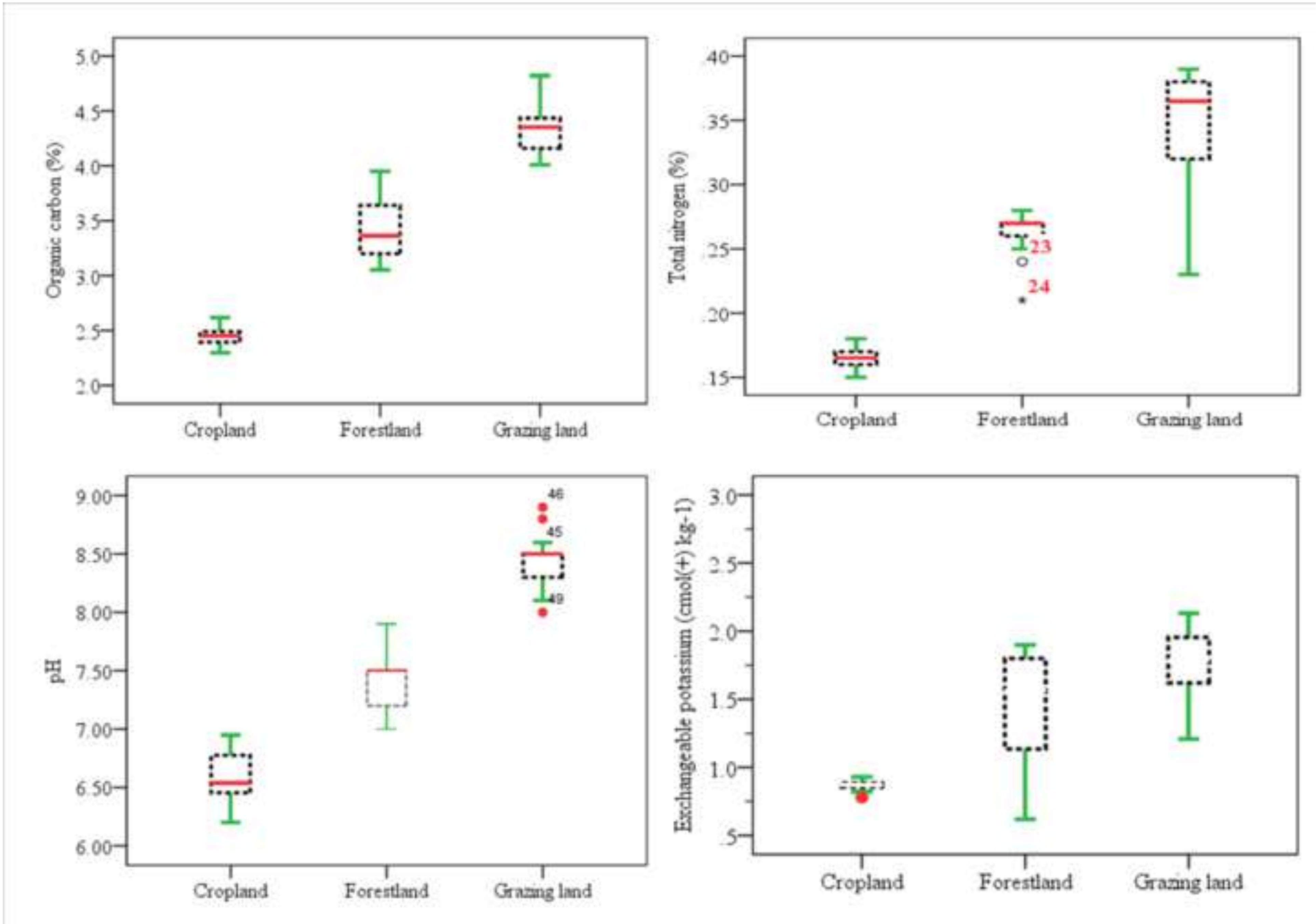

Figure

Click here to access/download;Figure;Fig 6.tif

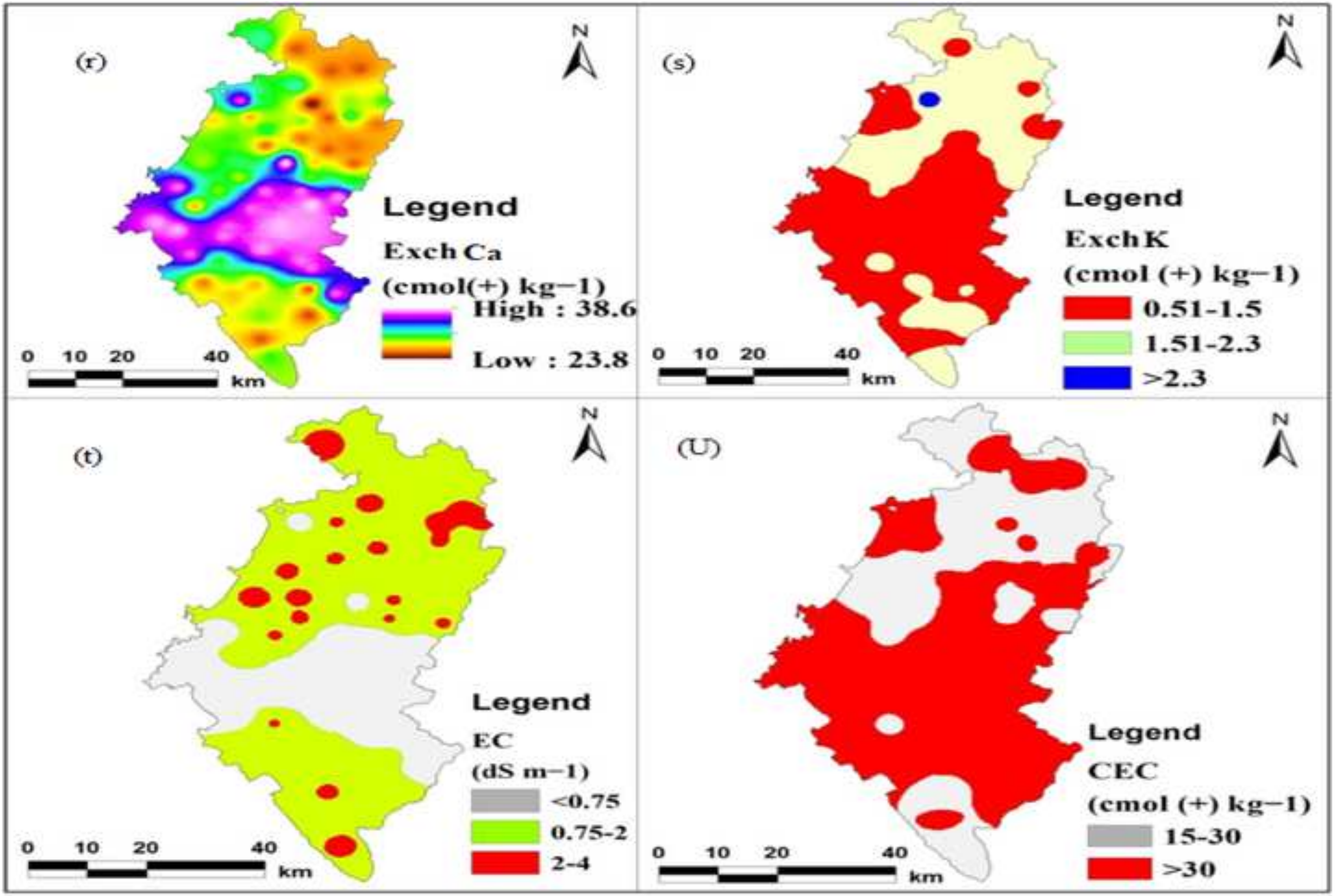

S1_Table

Click here to access/download
Supporting Information
S1_Table.xlsx

S2_Fig

Click here to access/download
Supporting Information
S2_Fig.tif.tif

S1_Fig

Click here to access/download

Supporting Information

S1_Fig.tif

Spatial variability of soil chemical properties under different land-uses in Northwest Ethiopia

Gizachew Ayalew[1,#a¶], Tiringo Yilak[2¶], Derege Tsegaye Meshesha[3¶], Eduardo Saldanha, Vogelmann[4¶], José Miguel Reichert[5¶], Nigussie Haregeweyn[6¶]

[1] Department of Natural Resources Management, Faculty of Agriculture and Environmental Sciences, Debre Tabor University, P. O. Box 272, Debre Tabor, Ethiopia

[2] Department of Plant Sciences, Faculty of Agriculture and Environmental Sciences, Debre Tabor University, P. O. Box 272, Debre Tabor, Ethiopia

[3] Geospatial Data and Technology Center, College of Agriculture and Environmental Sciences, Bahir Dar University, P. O. Box 5501, Bahir Dar, Ethiopia

[4] Biological Sciences Institute, Federal University of Rio Grande, São Lourenço do Sul, Brazil

[5] Soils Department, Federal University of Santa Maria (UFSM), Av. Roraima 1000, 97105-900 Santa Maria, RS, Brazil

[6] International Platform for Dryland Research and Education, Tottori University, Tottori, Japan

[#a] Current Address: Department of Natural Resources Management, College of Agriculture and Environmental Sciences, Bahir Dar University, P. O. Box 5501, Bahir Dar, Ethiopia

* Corresponding author: E-mail: tiruneh1972@gmail.com

Abstract

The understanding of the spatial variation of soil chemical properties is critical in agriculture and the environment. To assess the spatial variability of soil chemical properties in the Fogera plain, Ethiopia, we used Inverse Distance Weighting (IDW), pair-wise comparisons, descriptive analysis, and principal component analysis (PCA)~~ were used to evaluate the spatial variability of soil chemical properties under different land-uses of Fogera plain in the northwest region of in Ethiopia~~. In 2019, ~~S~~soil samples were collected at topsoil (a soil depth of 0-20 cm) from three representative land-uses (cropland, plantation forestland, and grazing lands) using a grid-sampling design~~ 2019~~. The ~~analysis of ~~variance analysis for soil pH, available phosphorus (avP), organic carbon (OC), total nitrogen (TN), electrical conductivity (EC), exchangeable potassium (exchK), ~~and~~ exchangeable calcium (exchCa), and cation exchange capacity (CEC) revealed ~~showed~~ significant differences~~variations~~ between ~~among~~ the land-uses. The highest mean values of pH (8.44), avP (31.51 ppm), OC (3.49 %), TN (0.313 %), EC (2.22 dS m^{-1}), and exchK (3.6 cmol(+) kg^{-1}) were determined under grazing land. The lowest pH (6.58), OC (2.44 %), TN (0.17 %), exchK (0.87 cmol(+) kg^{-1}), and EC (0.13 dS m^{-1}) were recorded in cultivated land. ~~None of the soil variables showed high variability (coefficient of variation ≥35 %) over the study area. Besides,~~ The PCA result revealed that the land-use change was responsible for most soil chemical properties, accounting for 98.55 %. Soil maps can help identify the nutrient status, update management options, and increase productivity and profit. Moreover, it promotes soil health and reduces environmental effects. The expansion of cultivated lands resulted ~~caused~~ a significant ~~substantial~~decrease~~ decline~~ in soil organic ~~carbon and total nitrogen~~matter. Thus, soil management strategies should tailor to replenish soil nutrient content while~~and~~ maintaining agricultural productivity ~~with~~in the Fogera plain.

Keywords: Inverse Distance Weighting; land-use; soil properties; spatial variability

Introduction

Environmental degradation caused by irrelevant ~~inappropriate~~ land-use is a global ~~worldwide~~ problem in sustainable agriculture. Land-use change markedly affects soil properties [1, 2]. Changing land-use from forest cover to plough-land may result~~could create~~ a ~~dec~~ decrease ~~line~~ in soil fertility, nutrients, and thus productivity [3-6], as well as ~~and~~ increased soil perturbation [5, 7-12].

~~In Ethiopia, r~~Rapid population growth and environmental factors in Ethiopia ~~lead to~~ have resulted in converting~~the conversion of~~ forestland and grassland ~~in~~to cultivated land [13]. ~~[14] examined that theT~~ The expansion of cultivated areas has ~~can~~ a substantial~~ly~~ influence ~~affect~~ on soil nutrient content [14]. [15] reported changes in the amount of soil organic carbon~~,~~ and total N as a result of ~~due to~~ changes in land-use and land-cover in the Gerado catchment, northeastern Ethiopia. [16] also investigated that deforestation has led to ~~resulted in~~ the deterioration of soil organic matter ~~in the soil~~. As a result,~~Thus,~~ soil nutrient deficiency~~depletion~~ is a critical ~~serious~~ problem in the country and a major crop production constraint ~~in the country~~ [17-18].

Ethiopia has seen an increase ~~has been showing an increase~~ in cultivated lands and eucalyptus plantations, and a decrease in grazing lands, because of ~~due to the~~ population growth [13]. The eucalyptus plantation had a significant impact on ~~considerably affected~~ soil properties [19-21]. [22] reported ~~stated~~ a reduction~~decrease~~ in soil organic carbon (OC), total nitrogen (TN), exchangeable cations, and cation exchange capacity (CEC) contents were owing to the shift of woodlands to croplands and grazing lands in the same country. [23] also found a declin~~e~~ing in pH ~~value~~ and ~~the~~ soil organic matter content in cultivated land in Ethiopia's ~~the~~ Kabe watershed.~~, Ethiopia.~~ As a result, ~~Thus,~~ scientific records ~~information~~ of spatial variability and distribution of soil properties among land-use shifts are critical for ~~to~~ optimizing ~~e~~ fertilizer use and increasing~~boost~~ crop productivity [20] .

In developing countries, including Ethiopia, land-use/land-cover change is a significant source of greenhouse gases (GHGs) such as carbon dioxide (CO_2), nitrous oxide (N_2O), and methane (CH_4) emissions [24]. Furthermore, nitrogen-containing fertilizers [25-26], tillage [26]; and complete removal of vegetation and residues [27] have influenced spatial variability, soil nutrient cycling, and GHGs emission. Thus, information on the spatial variability of soil due to and-use change is critical in this regard.

Accurate and scientific information about soils is essential for developing effective soil management techniques that sustain agricultural production while maintaining environmental quality. As a result, in Ethiopia, where the provision of soil information is scarce, assessing spatial variability distribution on soil properties in relation to land use is critical for predicting rates of soil functions and processes. Furthermore, site-specific management of pH, organic carbon, available N, available P, and available K [28] improves input use efficiency [29], increases crop production economic returns, and reduces ecological risks [30].

~~Recently, m~~Many researchers ~~scholars~~ have recently used~~been using~~ geostatistics to estimate ~~for estimating~~ the spatial variability of soil properties [31-34]. In geostatistics, the inverse distance weighting (IDW) model can be used to map the spatial distribution of any soil property measured for spatially distributed samples [35]. Understanding~~Knowledge of~~ the spatial variability of soil properties [36] and developing site-specific recommendations [36-37] are critical ~~vital~~ for optimizing nutrient usage, improving crop performance~~productivity~~, and minimizing environmental risks ~~hazards~~ [38]. This information, however, is scarce, and there are few ~~However, this information is scarce and there are few~~ studies on ~~the~~ state of the art and assessments on the situation of soil degradation in Ethiopia.

Furthermore, Besides, the spatial information produced using geostatistical techniques would be generated using geostatistical techniques about soil properties, soil fertility status, and nutrient management an input to improve food security and obtain enhance food security and obtain sustainable yield in developing countries, including Ethiopia, has have never been thoroughly investigated well studied using spatial prediction models [39]. Understanding of different land's Knowledge of the soil fertility of various land-use types that could be used for predicting, monitoring, and evaluating could be used to predict, monitor, and evaluate the effects of changes in land-use types on soil properties, scheming appropriate land-use planning, and sustaining the productivity of agriculture. In this regard, previous researches on , the Fogera plain area have area have not yet not been yet adequately discussedaddressed the Fogera plain area.

Thus, the target objective of this research paper was to assessevaluate the effectsimpacts of land-use types changes on spatial variability and distribution of soil fertility qualities, such asincluding pH, organic carbon (OC), total nitrogen (TN), available phosphorus (avP), exchangeable calcium (Ca^{2+}), exchangeable potassium (K^{+}), electrical conductivity (EC), and cation exchange capacity (CEC) in Fogera plain, the highland of Ethiopia.

Materials and methods

Description of the study area

The research study was performed was conducted within the Fogera plain area (37º 0' 0" E - 38º 45' 0" E and 11º 15' 0" N-12º 15' 0" N) in the northwest highland of Amhara region, Ethiopia (Fig 1). It has covers an a total area of 5,646 hectares. The topography of the study area is flat land. The main crops cultivated in the study area are rRice (*Oryza sativa* L.), maize (*Zea mays* L.), and *Teff* [*Eragrostis tef* (Zucc.)] are the main crops grown in the study region. Rice production (76 %) is primarily a subsistence farming operation in the study

area. Crop residues are collected for use as fuelwood or animal feed. As a result, no crop residue remains in the field to serve as a source of organic amendments. Rice, onions, and eucalyptus products The are important income sources of income for the local people. are rice, onion, and eucalyptus products.

Fig 1. The study area's location: (a) Ethiopia, (b) Amhara region, and (c) soil sampling.

Sources of Spatial spatial data sources and their extraction

The study area's Lland-use/land-covers of the study area for 2019 wasere deriveddeduced from an Ethiopia Mapping Agency (EMA) 1:20,000 scale land-use/land-cover map made by the Ethiopia Mapping Agency (EMA), andmap, and also the mainjor land-use system consisted of cropland (4,362.73 ha, 77.27 %), plantation forestland (6,96.29 ha, 12.33 %) and grassland (586.98 ha, 10.39 %) [40]. We also performed reconnaissance surveys from in August – Novemberto November/2019 to validateerify the map. Using ArcGIS software version 10.5, A a digital elevation model (ASTER DEM) with a resolution of 20 * 20 m resolution, downloaded from the EMA website [40], was usedemployed to generate elevation and slope of the study area using ArcGIS software version 10.5.

As shown Figs 2-3, agroecology belonging to Kolla (<1800 m a.s.l.), Weyna dega (1,800–2,400 m a.s.l.), and dega (>2400 m a.s.l.) has an area share of 16.24 %, 71.93 %, and 11.83 %, respectively (Fig 2). According to Tthe digital soil map obtained collected from Water and Land Resource Centre (WLRC), shows that the soil types in of the Fogera plain region area are Chromic Vertisols (48.57 %), Eutric Nitosols (17.65 %), Orthic Luvisols (15.75 %), Eutric Cambisols (10.78 %), Chromic Luvisols (3.97 %), and Lithosols (3.27 %) [41].

Fig 2. Land-use of the area: (d) Eucalyptus plantation forestland, (e) Cultivated land, and (f) Grazing land.

Fig 3. Different maps of the study area, particularly (g) Soil types, (h) Altitude, and (i) Slope.

Soil sampling, laboratory analysis, and statistical analysis

We used Tthe fishnet tool -included with ArcGIS software version 10.5 was used to build generate a regularly spaced grid of sampling points on the land-uses in the study region. Following that, Next,60 representative soil samples (20 from each use: cropland, plantation forestland, and grazing land) were identified at topsoil (a depth of 0-20 cm) were recognized through in February-April/2020 using a systematic purposive approach.. in February-April/2020. The topsoil was chosen as it is most influenced by plants and soil management practices. To record each grid center in the field and the latitudes, longitudes, and elevations of sampling points, a A portable Global Positioning System (Garmin 60; 2 m accuracy) was used. Soil sampling locations were chosen to better reflect each land-use condition by taking topographic features and soil conditions into account [42]. Each soil sample was created by compositing five sub-samples, which could improve sampling intensity and lower soil analysis costs [43-44]. A kilogram of composite soil sample per site was collected from each location, air-dried, groundinded using a mortar and pestle, and analyzed at the Amhara Regional Soil Laboratory Center in accordance with national standard research methods [45].

Soil pH was measured potentiometrically in H_2O at the soil to solution ratio of 1: 2.5 using a combined glass electrode pH meter (Model CP-505, Zabrze ul, Poland) [46]. The Eelectrical conductivity (EC) of the soil was determinedmeasured using an EC meter at the soil to water ratio of 1: 5 using an EC meter (Orion Model 145, USA) [47]. The Walkley-Black procedures were used to measure Ssoil organic carbon (OC). was determined following the Walkley-Black procedures. A weighed portionamount (1-2 gm 20-50 mg) of the dried, ground soil samples were as treated with 5 ml of 0.4 N potassium dichromate solution ($K_2Cr_2O_7$) followed by the addition of 10 ml of concentrated sulfuric acid. The mixture was gently mixed and left for 16-18 hours before being given and then, 100 ml of triple-distilled water. was added to the mixture. The excess of dichromate was back-titrated

potentiometrically with the standard 0.2 N ferrous ammonium sulfate solution. The acidic dichromate was Bblankly titrated ion of the acidic dichromate with ferrous ammonium sulfate solution was performed [448].

TheT total nitrogen (TN) of the soils was determined through digestion, distillation, and titration procedures of the Kjeldahl using the Kjeldahl apparatus (Gallenhamp, USA) [49]. The soil'sA available phosphorus (avP) in the soil was measured determined using the 0.5 M $NaHCO_3$, pH of 8.5, in 1: 20 of a soil to solution ratio of- 1: 20 for half an hour. The (avP) was extracted with 1 M ammonium chloride, 0.5 M ammonium fluoride, 0.1 M sodium hydro-oxide (from Blulux Laboratory Reagent (p) Ltd), and the amount was measured by ameasured using a spectrophotometer (UV1700, Japan) -[50-51]. Exchangeable calcium (Ca), magnesium (Mg), potassium (K), and sodium (Na) were determined by saturating the soil samples with 1 M ammonium acetate solution at pH 7.0. Subsequently, Ca and Mg was were determined by using Perkin-Elmer Model 290 atomic —absorption spectrophotometer (ColVisTec, Germany); while exchangeable Na and K were measured by a Model 18 Perkin-Elmer flame photometer [52].

The soil's cation exchange capacity (CEC) of the soil was calculateddetermined by replacing from the NH_4^+ saturated samples that were replaced bywith K^+ from a percolated KCl solution (from LOBA CHEMIE PVT.LTD). The excess salt was removed by wWashing with ethanol (from LOBA CHEMIE PVT.LTD) eliminated , and the excess salt, and NH_4^+ was displaced by K^+ [53]. All- of the chemicals and reagents, such as including potassium dichromate, sulphuric acid, ferrous sulfate, ferroin, sodium bicarbonate, ammonium chloride, ammonium fluoride, and ammonium acetate, -were supplied by Merck KGaA and Sigma-Aldrich, Steinheim, Germany, unless otherwise mentioned. specified.

The variation of soil organic carbon, total nitrogen, and potassium was defined using the Inverse Distance Weighting (IDW) model [54-55]. Furthermore, IDW has been widely

used by scholars for the prediction of soil OM and soil nitrate [56]-, P and K levels [57], and soil pH scale [58]. the The current study used employed IDW to for map the spatial distribution of the soil chemical properties under the ArcGIS environment. Besides, pair-wise comparisons, and descriptive analysis, and principal component analysis (PCA) were performed carried out using Statistical Analyses System (SAS) software version 9.4 and the Statistical Package for Social Sciences (SPSS) software version 20 24, respectively. Means were compared using the Tukey test at 1% probability.

Ethics statement

Debre Tabor University's Research and Publication Directorate and Bahir Dar University's The Research and Publication Directorate authorized approved the present study to collect soil samples and access the field site. Farmers agreed to allow us collect the soil samples in the study area, as As the present research hasstudy has no harmful effects negative impacts on a humans., farmers allowed us to collect the soil samples in the study region.

Results and discussion

Effect of land-use/land-cover types on soil fertility quality

Soil pH_{H2O}

Based on the scores set by [46], a substantial part of the soils of *eucalyptus* plantation forestland (79.87 %), grazing land (68.90 %), and cropland (59.69 %) was moderately alkaline (7.4-8.4).The pH of the soil varied significantly ($P < 0.05$) depending on land-use. The soil pH values were found to be the highest (8.44) and the lowest (6.58) under the grazing and the cultivated lands, respectively (Table 1 and Figs 4-5). According to [59], Hhigher soil pH levels -obtained from plantation forestland and grazing land may ight be due to because of the presence existence of basic cations emanated from weathering [23], - and the potash obtained from ashes [60].

Table 1. Soil pH$_{H2O}$ rating, its area share (ha, %) by land-use, and its parametric test.

Formatted: Caption,Caption Char,style3,Char,style4,Caption 1,Caption Char Char Char,Caption Char Char Char Char Char Char, Line spacing: single

Common name	pH rating	Area share (ha, %) by land-use					
		Cropland		Plantation forestland		Grazing land	
		Area (ha)	%	Area (ha)	%	Area (ha)	%
Moderately acid	5.6-6.5	181.84	4.17	1.11	0.16	9.50	1.62
Neutral	6.6-7.3	1,576.93	36.15	139.05	19.97	173.04	29.48
Moderately alkaline	7.4-8.4	2,603.96	59.69	556.12	79.87	404.44	68.90
Total		4,362.73	100	696.29	100	586.98	100
Mean pH		6.58^{a}		7.40^{b}		8.44^{c}	

Means of pH with different letters are significantly varieddifferent (Tukey, $p < 0.01$).

Available phosphorus (avP)

A notable variation in Ssoil avP –varied significantly according towas obtained to land-uses. Following the limit established by [61], rated very high (>20 ppm) avP content was inobserved in soils of all land-uses. Following the limit set by [46], high levels (>20 ppm) were recorded (Table 2 and Figs 4-). 3-4). Higher avP values in soils could be might be revealed by the recurrent use of mineralized phosphorus [38], and the addition of manure, compost, and ashes [62] , presence of weathered soil minerals [63], and actions of microbes. Higher levels of avP in the soils indicate that the soils have optimum the nutrients for crop growth. [22, 64] reported similar findings made related reports in Ethiopia.

Table 2. Soil available phosphorus (avP) rating and its area share (ha, %) by land-use and its parametric test.

Common name	avP rate	Area share (%) by land-use					
		Cropland		Plantation forestland		Grazing land	
	ppm	Area (ha)	Area (%)	Area (ha)	Area (%)	Area (ha)	Area (%)
Medium	11-14	273.66	6.27	35.02	5.03	23.05	3.93
High	15-20	1,425.74	32.68	200.90	28.85	118.12	20.12
Very high	>20	2,663.33	61.05	460.37	66.12	445.82	75.95
Total		4,362.73	100	696.29	100	586.98	100
Mean avP (ppm)		21.32^{a}		11.43^{b}		32.52^{c}	

Means of available P with different letters are significantly different (Tukey, $p < 0.01$).

Fig 4. Maps of (n) Soil pH, (o) Organic carbon, (p) Total nitrogen, and (q) Available phosphorus.

Soil organic carbon (OC) and total nitrogen (TN)

Land-use changes caused a significant difference A significant variation in soil organic carbon (OC) and total nitrogen (TN). was recorded from land-uses. According to the ranking set by [65] As per the rating set by [52], low organic carbon content (2.44 %) in the soils dominated the agricultural landcultivated land (87.66 %), grazing land (81.7 %), and eucalyptus plantation forestland (80.63 %), as shown in (Table 3 and Figs 4-5 3-4). The highest (4.35 %) and lowest (2.44 %) OC contents obtained in grazing and croplands demonstrated that soil OC showed a better response to land-use type. This It may be attributed could occur due to the high erosion rates, crop residue exclusion, increased rate of mineralization rates, and the nutrient deficiency of nutrients [38, 66]. The higher organic carbon and available P contents of the grazing lands suggest indicate that OM is the primary source principal supply of avP [67].

Table 3. Soil organic carbon (OC) rating and its area share (ha, %) by land-use and its parametric test.

	OC	Area share (ha, %) by land-use

Formatted: Caption,Caption Char,style3,Char,style4,Caption 1,Caption Char Char Char,Caption Char Char Char Char Char Char, Line spacing: single

Common name	rate	Cropland		Plantation forestland		Grazing land	
	%	Area (ha)	Area (%)	Area (ha)	Area (%)	Area (ha)	Area (%)
Low	2-4	3,824.5	87.66	561.44	80.63	479.59	81.70
Medium	4-8	538.23	12.34	134.85	19.37	107.40	18.30
Total		4,362.73	100	696.29	100	586.98	100
Mean OC (%)		2.44^{a}		3.43^{b}		4.35^{c}	

Means of soil OC with different letters are significantly different (Tukey, $p < 0.01$).

Fig 5. Mean values of soil parameters using box plots.

According to the rate of [61], the cropland (16.29 %) and plantation forestland (69.14 %) demonstrated, dominantly exhibited, respectively, low (0.17 %) and high total nitrogen (0.35 %) contents in the soils (Table 4 and Figs 4-53 4). In line with this, croplands had lower soil OC content was obtained in the croplands [2, 23, 68]. Higher soil OC and TN contents found recorded atin plantation forests and grazing lands are most likely due to probably attributed to the grass burning of grass and deposition of dung deposition, respectively. Furthermore, Besides, researchers have advocated encouragedfor grazing in order to sustain maintain nutrient cycling and decomposition rates [69]. The low total nitrogen content s may be might be eligible qualified to minimize the loss of nitrogen loss by through volatilization or leaching and rapid decomposition of OM. As a result, Therefore, the conversion shift of grassland and eucalyptus plantation to cultivated land worsens exacerbates soil OC and TN decline [70]. Thus, for long-term development, the soils call for need external nitrogen and carbon inputs for sustainable production.

Table 4. Soil total nitrogen (TN) rating and its area share (ha, %) by land-use and its parametric test.

Common name	TN rate (%)	Area share (ha, %) by land-use					
		Cropland		Plantation forestland		Grazing land	
		Area (ha)	Area (%)	Area (ha)	Area (%)	Area (ha)	Area (%)
Low	0.1-0.2	1,219.37	27.95	69.66	16.29	115.14	19.62
Medium	0.2-0.3	2,159.42	49.50	62.31	14.57	244.65	41.68
high	0.3-0.4	983.94	22.55	295.63	69.14	227.18	38.70
Total		4,362.73	100	427.60	100	586.98	100
Mean TN (%)		0.17[a]		0.27[b]		0.35[c]	

Means of TN with different letters are significantly different (Tukey, $p < 0.01$).

Exchangeable cations (K, Ca) and electrical conductivity (EC)

As shown in Tables 5-6 and Figs 5-6, there was a significant variation in ~~of~~ the contents of soil K, Ca, and EC based on ~~was observed from~~ land-use~~s~~. Besides, according to [59, 61], the soils in the study region ~~area~~ had ~~consisted of~~ high ~~contents of~~ Ca (>20 cmol$_{(+)}$ kg^{-1}) and K (0.51-1.51 cmol$_{(+)}$ kg^{-1}) contents ~~as per the rates by [46, 48]~~. In the soils, higher Ca and K levels were found. It might be due to ~~attributable to~~ the type of parent materials, weathering, land-use types, fertilizer types ~~used~~ and ~~their~~ leaching rates, crop remains, and litterfall [71] ~~(Uzoho & Ekeh, 2014)~~. The higher ~~contents of~~ Ca and K contents present in both grazing and plantation forestlands are associated with ~~could be linked to its~~ the higher pH value [72] and clay particles [73]~~were stated~~. On the other hand, the soils showed slightly salty.

This result suggests that Ca and K do not appear~~seem~~ to be limiting nutrients to crop production in the region~~area~~. Based on the EC rate established by [74], no significant ~~amount of soluble salts~~ amounts of soluble salts were ~~was~~ accumulated, implying ~~thus~~ that plant growth and development would be ~~will not~~ unaffected. ~~affect plant growth and development.~~

Formatted: Caption,Caption Char,style3,Char,style4,Caption 1,Caption Char Char Char,Caption Char Char Char Char Char Char, Left

Formatted Table

Formatted: Centered

Fig 6. Maps: (r) Exchangeable calcium, (s) Exchangeable potassium, (t) Electrical conductivity, and (u) Cation exchange capacity.

Table 5. Soil exchangeable potassium (exchK) rating and its area share (ha, %) by land-use and its parametric test

Common name	exchK rRate	Area share (ha, %) by land-use					
		Cropland		Plantation forestland		Grazing land	
	$cmol_{(+)}\ kg^{-1}$	Area (ha)	Area (%)	Area (%)	Area (ha)	Area (%)	Area (ha)
High	0.51-1.51	2,609.17	59.81	320.17	45.98	257.26	43.83
Medium	1.51-2.3	1,733.37	39.73	373.31	53.61	326.57	55.64
Very high	>2.3	20.19	0.46	2.80	0.40	3.15	0.54
Total		4,362.73	100	696.29	100	586.98	100
Mean exchK ($cmol_{(+)}\ kg^{-1}$)		0.87^{a}		1.47^{b}		1.81^{c}	

Means of exchK with different letters are significantly different (Tukey, $p < 0.01$).

Formatted: Caption,Caption Char,style3,Char,style4,Caption 1,Caption Char Char Char,Caption Char Char Char Char Char Char, Line spacing: single

Formatted Table

Table 6. Electrical conductivity (EC) rating and its area share (ha, %) by land-use and its parametric test.

Formatted: Caption,Caption Char,style3,Char,style4,Caption 1,Caption Char Char Char,Caption Char Char Char Char Char Char, Line spacing: single

Common name	EC ~~R~~rate dS m^{-1}	Area share (ha, %) by land-use					
		Cropland		Plantation forestland		Grazing land	
		Area (ha)	Area (%)	Area (ha)	Area (%)	Area (ha)	Area (%)
Not salty	<0.75	1,427.14	32.71	96.07	13.80	146.18	24.90
Slightly salty	0.75-2	2,660.19	60.98	538.15	77.29	372.90	63.53
Moderately salty	2-4	275.30	6.31	62.07	8.91	67.90	11.57
Sum		4,362.63	100	696.29	100	586.98	100
Mean EC (dS m^{-1})		0.13^{a}		1.21^{b}		2.22^{c}	

Means of EC with different letters are significantly different (Tukey, $p < 0.01$).

Cation Exchange Capacity (CEC)

According to ~~As shown in Table 7, Fig 6, and~~ the rate set by~~–~~ [61], a significant variance ~~variation~~ and higher CEC indicated that soils in ~~of~~ the study area have a high capacity ~~potential~~ to retain ~~hold~~ nutrients against leaching losses (Table 7 and Fig 6). The highest CEC values recorded in cultivated land-use may ~~might~~ be due to ~~caused by~~ soil organic material, pH, quantity, and type ~~kind~~ of clay, which adsorb and retain ~~hold~~ positive cations through electrostatic force [75]. The current findings were also consistent ~~in agreement~~ with [76-78], who reported higher CEC under cultivated lands in Ethiopia's ~~the~~ highlands ~~of Ethiopia~~.

Table 7. Cation exchange capacity (CEC) rating and its area share (ha, %) by land-use and its parametric test.

Common name	CEC rate	Area share (ha, %) by land-use					
		Cultivated land		Plantation forestland		Grazing land	
	$cmol_{(+)}\ kg^{-1}$	Area (ha)	Area (%)	Area (ha)	Area (%)	Area (ha)	Area (%)
High	15-30	1,402.31	32.14	357.07	51.28	275.72	46.97
Very high	>30	2,960.42	67.86	339.22	48.72	311.26	53.03
Sum		4,362.73	100	696.29	100	586.98	100
Mean CEC		40.36^{a}		31.53^{b}		25.24^{c}	

Means of CEC with different letters are significantly different (Tukey, $p < 0.01$).

Descriptive statistics of soil fertility parameters

Table 8 shows the highest (8.44) and the lowest (6.58) soil pH values were observed under the grazing and the cultivated lands, respectively. The highest (4.35 %) and lowest (2.44 %) OC contents obtained in the grazing and croplands demonstrated that soil OC showed a strong response to land-use type [63]. Following the limit set by [46], high avP levels (>20 ppm) and very high CEC (>20 $cmol_{(+)}\ kg^{-1}$) were observed in all land-uses. Based on the EC rate established by [64], no significant amount of soluble salts was accumulated, thus will not affect plant growth and development.

Formatted: Caption,Caption Char,style3,Char,style4,Caption 1,Caption Char Char Char,Caption Char Char Char Char Char Char, Line spacing: single

Table 8. Descriptive information soil chemical properties within the study area.

Statistic	LULC	Soil fertility parameters							
		pH_{H2O}	OC	TN	AvP	Exch Ca	Exch K	CEC	EC
			%	%	ppm	cmol$_{(+)}$ kg^{-1}			dS m^{-1}
Standard deviation	CL	0.21	0.08	0.01	0.52	0.44	0.03	0.44	0.01
	FL	0.25	0.27	0.02	0.28	0.89	0.37	0.28	0.05
	GL	0.22	0.26	0.04	0.29	1.10	0.37	0.29	0.04
Coefficient of variation	CL	3.12	3.28	4.17	2.42	1.18	3.79	1.08	10.00
	FL	3.31	7.96	6.18	2.47	3.47	25.37	0.88	3.72
	GL	2.59	5.89	11.56	0.88	3.67	20.50	1.13	1.89
Kurtosis	CL	-0.67	0.34	0.08	1.78	0.86	2.28	-0.54	6.58
	FL	-0.31	-0.61	6.29	-1.27	0.35	-0.66	-0.97	-0.39
	GL	0.38	-0.53	2.23	-0.89	0.33	2.85	0.80	-0.59
Skewness	CL	0.14	0.08	0.00	-1.44	1.10	-0.73	0.36	2.14
	FL	-0.01	0.64	-2.27	0.12	0.32	-0.53	-0.29	-0.69
	GL	-0.14	0.63	-1.38	-0.15	-0.33	0.95	1.19	-0.53
Minimum	CL	6.00	2.00	0.15	20.01	37.00	1.00	40.00	0.00
	FL	7.00	3.00	0.21	11.01	24.00	1.00	31.00	1.00
	GL	8.00	4.00	0.23	32.04	27.00	1.00	25.00	2.00
Maximum	CL	7.00	3.00	0.18	21.94	39.00	1.00	41.00	0.00
	FL	8.00	4.00	0.28	11.92	28.00	2.00	32.00	1.00
	GL	9.00	5.00	0.39	32.99	32.00	3.00	26.00	2.00

OC, organic carbon; TN, total nitrogen; avP, available phosphorus; ExchCa, exchangeable Calcium; ExchK, exchangeable potassium; CEC, cation exchange capacity, EC, electrical conductivity; LULC, land-use/land-cover; CL, cropland; FL, forestland; GL, grazing land.

As indicated in Table 8 and S1 Fig., the skewness results, which varied from -2.27 to 2.14, showed almost all soil properties of the various land uses were normally distributed. None of the soil variables was categorized as high variability in terms of coefficient of variation (CV≥35 %). Based on the rate established by [65], soil pH, Ca, CEC, OC, TN, and

avP had low variability (CV≤15 %), which could be because of the area's high spatial homogeneity. Similar findings were also made in the country [15, 66]. Thus, the change of forestland to croplands aggravates the degradation of organic matter that affects most of the soil properties [67]. However, compost, cowl plants, manure, minimal tillage, and crop rotation could enhance organic matter content in soils [68]. Moderate variation (CV=15-34 %) in potassium at forestland and grazing land is possible because of the variations in soil management practices, parent material, and groundwater depth [19, 69].

Principal component analysis (PCA) of soil chemical properties

The two principal component analyses (PCA) with eigenvalues greater than one were able to explain the greatest variance (98.55%) of the analyzed soil chemical properties (S1 Table) [79]. Moreover, 79.97 % of the variation in data was explained by pH, organic carbon (OC), total nitrogen (TN), exchangeable potassium (exchK), electrical conductivity (EC), and cation exchangeable capacity (CEC) on the first PC. Simultaneously, the second component notably loaded the available phosphorus (avP) and exchangeable calcium (exchCa) (Table 8 and S1 Fig). As a result, the PCA reduces the dimensions and complexity of the soil data matrix [80].

Table 8. Principal component analysis of soil chemical properties about land-uses in Fogera plain, northwest Ethiopia

**Principal component**	**PC1**	**PC2**
Eigenvalue	6.07	1.29
Variance (%)	79.97	18.58
**Variables**	**Eigenvectors**	
pH	**0.96**	0.12
Available phosphorus	0.48	**0.86**
Organic carbon	**0.96**	0.03
Total nitrogen	**0.96**	0.00
Exchangeable potassium	**0.81**	-0.08
Exchangeable calcium	-0.67	**0.73**
Electrical conductivity	**0.99**	0.03
Cation exchangeable capacity	**-1.00**	0.04

Bold eigenvector values referred to highly weighted variables in the PC.

Formatted Table

Implications for sustainable soil fertility management and environmental conservation

Our results showed that the transition from grassland to cultivated land and eucalyptus plantation results in ~~leads to~~ a significant reduction in the total nitrogen within Fogera plain's topsoil. Organic materials may be used to reduce nitrogen depletion. Nevertheless, avP content of the area's soils ~~of the area~~ was high (>20 ppm), which could be attributed ~~due~~ to the frequent ~~repeated~~ use of mineralized phosphorus -[38]. The addition of nitrogen-containing fertilizer inputs might~~ay~~ also improve the cultivated lands' soil nutrient supply to ~~gain~~ better crop yield and farming profitability. However, the overuse of nitrogen and phosphorus fertilizer may lead ~~can contribute~~ to global climate change due to ~~through~~ their energy-intensive processing ~~production~~ and inefficient use– [81],~~;~~ eutrophication of

water bodies [82],; and soil acidification [83]. Besides, there has been an increased total greenhouse gas emissions, including N_2O, CO_2 have increased under cultivated lands, which depend on the rate of decomposition of organic materials in the soil [81]. Soil management methods, optimum N application rate [84], and the use of organic resources and nitrification inhibitors are all possible soil management approaches.

The information of spatial soil variability across land-uses is vital for sustainable land management practices, which in turn could reduce soil erosion, enhance land productivity, and improve farmers' the livelihood livelihoodof farmers, reduce GHGs, and maintain soil and environmental quality [85-86]. Furthermore, we should develop relevant land-use planning and policies should be developed to provide an optimal solution geared toward improving the soil's nutrient use efficiency and reducing the adverse environmental effects, including nitrate losses to water and N_2O emissions [87-88]-.

Conclusion

The majority of soil chemical properties across various land-uses were normally distributed (S2 Fig). We obtained A a substantial difference variation ofin soil pH, available phosphorus (avP), organic carbon (OC), total nitrogen (TN), electrical conductivity (EC), exchangeable bases (Ca and K), and cation exchange capacity (CEC) was obtained over across land-uses. Under these conditions, grazing land had the highest mean values of pH (8.44), avP (31.51 ppm), OC (3.487 %), TN (0.313 %), EC (2.22 dS m^{-1}), and exchK (3.6 $cmol_{(+)}$ kg^{-1}) were recorded under grazing land, while in cultivated land had the lowest OC (2.44 %), TN (0.17 %), soil pH (6.58), exchK (0.87 $cmol_{(+)}$ kg^{-1}), and EC (0.13 dS m^{-1}). were recorded in cultivated land. Most soil variables confirmed low spatial variability, which can be due to the area's high spatial homogeneity. Besides, PCA explained 98.55% of the soil variability because of land-use change. The expansion of cultivated lands depleted caused the

depletion of soil OC and TN, restricting limiting crop growth and decreasing declining crop yield.

Thus, soil management alternatives should gear to replenish the OC and N contents of the soil for increasing crop yield in the area. The interpolated maps of soil properties help farmers and decision-makers in improving soil management alternatives, optimizing fertilizer use, enhancing crop productivity, and reducing GHGs emissions. As small soil samples limited the study, many samples on the larger scale should gear in future studies to understand better the spatial variability of soil properties of the Fogera plain, Ethiopia.

Acknowledgment

Our grateful acknowledgment directs to Debre Tabor University and Bahir Dar University for the financial grant. We substantially recognized tThe Amhara Regional Soil Laboratory Center was substantially recognized for laboratory analysis for this study. Besides, we acknowledged the Water and Land Resource Centre (WLRC) and Ethiopian Mapping Agency (EMA) are also acknowledged for various data access.

Author contributions

Gizachew Ayalew designed the method and wrote the draft manuscript. Tiringo Yilak and Derege Tsegaye Meshesha have been accountable for data analysis, and interpretation of the results. Eduardo Saldanha Vogelmann, José Miguel Reichert, and Nigussie Haregeweyn analyzed/interpreted the results, reviewed/commented on the manuscript.

Declaration of competing interests

The authors declare that they have no competing interests.

References

1. Burst M, Chauchard S, Dambrine E, Dupouey JL, Amiaud B. Distribution of soil properties andalong with forest-grassland interfaces: influence of permanent environmental factors or land-use after-effects. Agric Ecosyst Environ. 2020; 289:106739. https://doi.org /10.1016/j.agee.2019.106 739.
2. Fentie SF, Jembere K, Fekadu E, Wasie D. Land-use and land-cover dynamics and soil properties of soils under different land-uses in the tejibara watershed, Ethiopia. Sci World JP. 2020; 1-12. https://doi.org/10.1155/2020/1479460.
3. Saha D, Kukal SS. Soil Structural Stability and Water Retention Characteristics under Different Land-uses of Degraded Lower Himalayas of North-West India. Land Degrad Dev. 2013; 26(3): 263-–271. doi:10.1002/ldr.2204.
4. Reichert JM, Rodrigues MF, Bervald CMP, Brunetto G, Kato OR, Schumacher MV. Fragmentation, fiber separation, decomposition, and nutrient release of secondary-forest biomass, mechanically chopped-and-mulched, and cassava production in the Amazon. Agric Ecosyst Environ. 2015; 204: 8-–16. https://doi.org/10.1016/j.agee.2015.02.005.
5. Reichert JM, Rodrigues MF, Bervald CMP, Kato OR. Fire-free fallow management by mechanized chopping of biomass for sustainable agriculture in eastern Amazon: effects on soil compactness, porosity, and water retention and availability. Land Degrad Dev. 2016a; 27: 1403–1412. https://doi.org/10.1002/ldr.2395.
6. Bufebo B, Elias E. Effects of Land-Use/Land-Cover Changes on Selected Soil Physical and Chemical Properties in Shenkolla Watershed, South Central Ethiopia. Advances in Agriculture,. 2020. https://doi.org/10.1155/2020/5145483.
7. Barua SK, Haque SMS. Soil characteristics and carbon sequestration potentials of vegetation in degraded hills of Chittagong, Bangladesh. Land Degrad Dev. 2013; 24: 63-–71. https://doi.org/10.1002/ldr.1107.

8. Suzuki LEAS, Reichert JM, Reinert DJ, Lima CLR de. Degree of compactness and mechanical properties of a subtropical Alfisol with eucalyptus, native forest, and grazed pasture. Forest Sci. 2015; 61:716–722. https://doi.org/10.5849/forsci.14-172.

9. Reichert JM, Amado TJC, Reinert DJ, Rodrigues MF, Suzuki LEAS. Land-use effects on subtropical, sandy soil under sandyzation/desertification processes. Agric Ecosyst Environ. 2016b; 233: 370–380. https://doi.org/10.1016/j.agee.2016.09.039.

10. Reichert JM, Cechin NF, Reinert DJ, Rodrigues MF, Suzuki LEAS. Ground-based harvesting operations of Pinus taeda affects the structure and pore functioning of clay and sandy clay soils. Geoderma. 2018; 331:38–49. https://doi.org/10.1016/j.geoderma.2018.06.012.

~~10.~~11. Holthusen D, Brandt AA, Reichert JM, Horn R. Soil porosity, permeability, and static and dynamic strength parameters under native forest/grassland compared to no-tillage cropping. Soil and Till Res. 2018a; 177:113–124. https://doi.org/10.1016/j.still.2017.12.003.

~~11.~~12. Holthusen D, Brandt AA, Reichert JM, Horn R, Fleige H, Zink A. Soil functions and in situ stress distribution in subtropical soils as affected by land-use, vehicle type, tire inflation pressure, and plant residue removal. Soil and Till Res. 2018b; 184:78–92. https://doi.org/10.1016/ j.still.2018.07.009.

13. Tesfahunegn GB. Soil quality indicators response to land-use and soil management systems in Northern Ethiopia's catchment. Land Degrad Dev. 2016; 27: 438–448. https://doi.org/10.1002/ldr.2245, 2016.

14. Biro K, Pradhan B., Muchroithner M, Makeschin F. Land-use/land-cover change analysis and its impact on soil properties in the northern part of Gadarif region, Sudan, Land Degrad Dev. 2013; 24: 90–102. https://doi.org/10.1002/ldr.1116.

15. Asmamaw LB, Mohammed AA. Effects of slope gradient and land-use/land-cover on selected soil physical and biochemical properties of the Gerado catchment, North-eastern Ethiopia. Int J Environ Stud. 2013; 70: 111–125. https://doi.org/10.1080/00207233.2012.751167.
16. Emiru N, Gebrekidan H. Effect of land-use changes and soil depth on soil organic matter, total nitrogen and available phosphorus contents of soils in Senbat watershed, western Ethiopia. J Agric Biol Sci. 2013; 8: 206–212.
17. Stoorvogel JJ, Smaling EMA. Assessment of soil nutrient depletion in Sub-Saharan Africa: 1983-2000. Vol. 2: Nutrient balances per crop and land-use systems (Report/ Winand Staring Centre; No. 28). ISRIC, 1990. https://edepot.wur.nl/3 05176 19.
18. Stoorvogel JJ, Smaling EM, Janssen BH. Calculating soil nutrient balances in Africa at different scales. Fertilizer res. 1993; *35*(3): 227-235.
19. Tadele D, Teketay D. Growth and yield of two-grain crops on sites former covered with eucalypt plantations in Koga Watershed, northwestern Ethiopia. J For Res. 2014; 25: 935–940. https://doi.org/10.1007/s11676-014-0483-9.
20. Jaleta D, Mbilinyi B, Mahoo H, Lemenih M. Eucalyptus expansion as relieving and provocative tree in Ethiopia. J Agric Ecol Res Int. 2016a; 6(3): 1-12. https://doi.org/10.9734/JAERI/2016/228 41.
~~13.~~21. Jaleta D, Mbilinyi B, Mahoo H, Lemenih M. Evaluation of land-use/land-cover changes and eucalyptus expansion in Meja watershed, Ethiopia. J Geo Environ Earth Sci Int. 2016b; 7(3): 1-12. https://doi.org/10.9734/JGEESI/2016/26606.
22. Fantaw Y, Abdu A. Soil property changes following the conversion of acacia woodland into grazing and farmlands in Ethiopia's rift valley area. Land Degrad Dev. 2011; 22:425–431. https://doi.org/10.1002/ldr.1022.

23. Assefa F, Elias E, Soromessa T, Ayele GT. Effect of changes in land-use management practices on soil physical and chemical properties in Kabe Watershed, Ethiopia. Air, Soil, and Water Res. 2020; 13: 1-16. https://doi.org/10.1177/1178622120939587.

24. Valentini R, Arneth A, Bombelli A, Castaldi S, Cazzolla Gatti R, Chevallier F, et al. A full greenhouse gases budget of Africa: synthesis, uncertainties, and vulnerabilities. Biogeosciences. 2014; 11(2):381-407. https://doi.org/10.5194/bg-11-381-2014.

25. Allen DE, Kingston G, Rennenberg H, Dalal RC, Schmidt S. Effect of nitrogen fertilizer management and waterlogging on nitrous oxide emission from subtropical sugarcane soils. Agric Ecosyst Environ. 2010; 136 (3-4): 209-217. https://doi.org/10.1016/j.agee.2009.11.002.

26. Venterea RT, Halvorson AD, Kitchen N, Liebig MA, Cavigelli MA, Del Grosso SJ, et al. Challenges and opportunities for mitigating nitrous oxide emissions from fertilized cropping systems. Front Ecol Environ. 2012; 10(10): 562-570. https://doi.org/10.1890/120062.

27. Hickman JE, Tully KL, Groffman PM, Diru W, Palm CA. A potential tipping point in tropical agriculture: Avoiding rapid increases in nitrous oxide fluxes from agricultural intensification in Kenya. J Geophys Res Biogeosci. 2015; 120(5): 938-951. https://doi.org/10.1002/2015JG002913.

28. Qiu SJ, Ju XT, Ingwersen J, Qin ZC, Li L, Streck T, et al. Changes in soil carbon and nitrogen pools after shifting from conventional cereal to greenhouse vegetable production. Soil and Till Res. 2010; 107(2): 80-87. https://doi.org/10.1016/j.still.2010.02.006.

29. Tesfahunegn GB, Tamene L, Vlek Paul LG. Catchment-scale spatial variability of soil properties and implications on site-specific soil management in northern Ethiopia. Soil and Till Res. 2011; 117:124–139. doi.org/10.1016/j.still.2011.09.005.

30. Yasrebi J, Saffari M, Fathi H, Karimian N, Emadi M, Baghernejad M. Spatial variability of soil fertility properties for precision agriculture in Southern Iran. J. Appl. Sci. 2008 8:1642-1650. https://doi: 10.3923/jas.2008.1642.1650.

31. Appel Neto E, Seidel EJ, Oliveira MS. Geostatistical-based index for spatial variability in soil properties. Rev Bras Cienc Solo. 2020; 44: e0200086. https://doi.org/10.36783/1806967r bcs20200086.

32. Ichami SM, Shepherd KD, Hoffland E, Karuku GN, Stoorvogel JJ. Soil spatial variation to guide the development of fertilizer use recommendations for smallholder farms in western Kenya. Geoderma Reg. 2020; p.e00300. https://doi.org/10.1016/j.geodrs.2020.e00300.

33. Jurišić M, Radočaj D, Krčmar S, Plaščak I, Gašparović M. Geostatistical Analysis of Soil C/N Deficiency and Its Effect on Agricultural Land Management of Major Crops in Eastern Croatia. Agronomy. 2020; 10(12): 1996. https://doi.org/10.3390/agronomy10121996.

34. Macedo Neto AAL, Farias PRS, Matos GSBD, Silva GBD, Santos AVFD, Anhê BB. Diagnosis and spatial variability of soil fertility and crop production in a teak area in eastern Pará State Cerne. 2020; 26(1): 37-47.https://doi.org/10.1590/01047760202026012683.

35. Burgess TM, Webster R. Optimal interpolation and isar1thmic mapping of soil properties, Eur J Soil Sci. 1980; 31:315-331. https://doi.org/10.1111/ejss.12749.

36. Guan F, Xia M, Tang X, Fan S. Spatial variability of soil nitrogen, phosphorus, and potassium contents in Moso bamboo forests in Yong'an City, China. Catena. 2017; 150: 161–72. https://doi.org/10.1016/j.catena.201.11.017.

37. Rosemary F, Vitharana UWA, Indraratne SP, Weerasooriya R, Mishra U. Exploring the spatial variability of soil properties in an Alfisol soil catena. Catena. 2017; 150: 53–61. https://doi.org/10.1016/j.catena.2016.10.017.

38. Panday D, Maharjan B, Chalise D, Shrestha RK, Twanabasu B. Digital soil mapping in the Bara District of Nepal using Kriging tool in ArcGIS. PLoS ONE. 2018; 13(10):e0206350. https://doi. org/10.1371/journal.pone.0206350.

39. Behera SK, Shukla AK. Spatial distribution of surface soil acidity, electrical conductivity, soil organic carbon content, and exchangeable potassium, calcium, and magnesium in some cropped acid Soils of India. Land Degrad Dev. 2015; 26: 71–79. https://doi.org/10.1002/ldr.2306.

40. Ethiopian Mapping Agency, P.O.Box 597, Addis Abeba. [Accessed 2019 January 18]. http://www.ema.gov.et/.

41. Water and Land Research Center (WLRC). National Geospatial database System EthioGIS-2/Release. 2014.

42. Buttafuoco G, Castrignanò A, Cucci G, Lacolla G, Lucà F. Geostatistical modelling of within-field soil and yield variability for management zones delineation: A case study in a durum wheat field. Precis. Agric. 2017; 18:37-58.

43. Reza SK, Baruah U, Sarkar D, Singh SK. Spatial variability of soil properties using geostatistical method: a case study of lower Brahmaputra plains, India. Arab. J. Geosci. 2016; 9(6): 446. https://doi:10.1080/03650340.2017.1296134.

44. Özyazici MA, Dengiz O, Sağlam M, Erkoçak A, Türkmen F. Mapping and assessment-based modeling of soil fertility differences in the central and eastern parts of the Black Sea region using GIS and geostatistical approaches. Arab. J. Geosci. 2017; 10(2): 45.

45. Bekele T, Sertsu S. Procedures for soil and plant analysis. National Soil Research Centre Ethiopian Agricultural Research Organization Technical paper No. 74, 2000. Addis Ababa, Ethiopia.

46. Van Reeuwijk LP. Procedure for soil analysis. 4th ed. International soil reference center Wageningen. (ISRIC) Technical Paper No. 9, 1993. The Netherlands, Wageningen.

47. USDA Soil Survey Laboratory manual, Soil Survey Investigation Report no. 42, version 4, USDA-NRCS, Nebraska, USDA. 2004.

48. Walkley A, Black CA. An examination of Degtjareff methods for determining soil organic matter and the proposed modifications of the chromic acid titration method. Soil Sci. 1934; 37: 29–38. https://doi.org/10.1097/00010694-193401000-00003.

49. Bremmer JM, Mulvaney CS. Nitrogen total. In: Page, A.L. (Ed.), Method of Soil Analysis. Part 2. Chemical and Microbiological Properties. Am. Soc. Agron., WI, Agron. Monogr. 1982; 9: 595–624. doi.org/10.2134/agronmonogr9.2.2ed.c31.

50. Olsen SR, Cole CV, Watanabe FS, Dean LA. Estimation of available phosphorus in soils by extraction with sodium bicarbonate. United States Department of Agriculture Circular. Washington, DC; 1954; p. 939. https://doi.org/10.1002/jsfa.2740660423.

51. Jackson ML. Soil chemical analysis. New Delhi, India: Prentice- Hall of India Private Ltd.. 1967.https://doi.org/10.1002/jpln.19590850311.

52. Rowell DL. Soil science: Methods and application, Addison Wesley Longman, Limited, England. 1994.

53. Chapman HD. Cation exchange capacity. In C.A. Black (Ed.). Methods of soil analysis – Chemical and microbiological properties, Madison, WI: J Am Soc Agron. 1965; 9: 891–901. https://doi.org/10.2134/agronmonogr9.2.c6.

54. Venteris E, Basta N, Bigham J, Rea R. Modeling Spatial Patterns in Soil Arsenic to Estimate Natural Baseline Concentrations. J. Environ. Qual. 2014; 43: 936-946. https://doi.org/10.2134/jeq2013.11.0459.

55. Tripathi R, Nayak AK, Shahid M, Raja R, Panda BB, Mohanty S, et al. Characterizing Spatial Variability of Soil Properties in Salt Affected Coastal India Using Geostatistics and Kriging. Arab. J. Geosci. 2015; 8: 10693-10703. https://doi.org/10.1007/s12517-015-2003-4.

56. Gotway CA, Ferguson RB, Hergert GW, Peterson TA. Comparison of kriging and inverse-distance methods for mapping soil parameters. Am J Soil Sci. 1996; 60:1237–1247. https://doi.org/10.2136/sssaj1996.03615995006000040040x.

57. Wollenhaupt NC, Wolkowski RP, Clayton MK. Mapping soil test phosphorus and potassium for variable-rate fertilizer application. J Prod Agric. 1994; 7(4): 441-448. https://doi.org/10.2134/jpa 1994.0441.

58. Robinson TP, Metternicht G. Comparing the performance of techniques to improve the quality of yield maps. Agric Syst. 2005; 85(1):19-41. https://doi.org/10.1016/j.agsy.2004.07.010.

59. EthioSIS (Ethiopia Soil Information System). Soil fertility status and fertilizer recommendation atlas for Tigray regional state, Ethiopia. Addis Ababa, Ethiopia: Agricultural Transformation Agency. 2014; 76 pp.

60. Moraes JFL, Volkoff B, Cerri CC, Bernoux M. Soil properties under Amazon forest and changes due to pasture installation in Rondonia. Brazil. Geoderma. 1996; 70 (1): 63–81. https://doi.org/10.1016/0016-7061(95)00072-0.

61. Hazelton P, Murphy B. Interpreting soil test results: what do all the numbers mean? Australia: CSIRO Publishing, Collingwood Victoria–Australia. http://www.publish.CSIRO. 2007; 160 p. https://doi.org/10.1111/sum.12402.

62. Miheretu BA, Yimer AA. Spatial variability of selected soil properties to land-use and slope position in Gelana sub-watershed, Ethiopia's northern highlands. Phys Geogr. 2018; 39 (3): 230–245. https://doi.org/10.1080/02723646.2017.1380972.

63. Assefa A, van Keulen H. Modeling soil nutrient dynamics under alternative farm management practices in the Northern Highlands of Ethiopia. Soil and Till Res. 2009; 103:203–215. https://doi.org/10.1016/j.still.2008.05.022.

64. Moges A, Dagnachew M, Yimer F. Land-use effects on soil quality indicators: a case study of Abo-Wonsho Southern Ethiopia. Appl Environ Soil Sci. 2013. https://doi.org/10.1155/2013/784989.

65. Food and Agriculture Organization (FAO). Soil and plant testing as a basis of fertilizer recommendations. Soils Bulletin No. 38/2. Rome. 1980; 100 pp.

66. Tellen VA, Yerima BPK. Effects of land-use change on soil physical and chemical properties in selected areas in Cameroon's North West region. Environ Syst Res. 2018; 7:3. https://doi.org/10.1186/s40068-018-0106-0.

67. Juo ASR, Manu A. Chemical dynamics in slash-and-burn agriculture. Agric Ecosyst Environ. 1996; 58:49–60. https://doi.org/10.1016/0167-8809 (95)00656-7.

68. Kassa Cholbe M, Kebede Yeme F, Haile Woldeyohannes W. Fertility Status of Acid Soils under Different Land-Use Types in Wolaita Zone, Southern Ethiopia. Appl Environ Soil Sci. 2020. https://doi.org/10.1155/2020/3713967.

69. McNaughton SJ, Banyikwa FF, McNaughton MM. Promotion of the cycling of diet-enhancing nutrients by African grazers. Science. 1997; 278: 1798–1800. https://doi.org/10.1126/ science.278.5344.1798.

~~14.~~70. Braimoh AK, Vlak PL. The impact of land-cover change on soil properties in northern Ghana. Land Degrad Dev. 2004; 15: 65–74. https://doi.org/10.1002/ldr.590.

71. Uzoho BU, C Ekeh. Potassium status of soils in relation to land use types in Ngor-Okpala, South-eastern Nigeria. J. Nat. Sci. Res. 2014; 4 (6):105–14.
72. Mesfin Abebe. The challenges and prospects of soil chemistry in Ethiopia. In: Teshome Yizengaw, Eyasu Mekonnen, and Mintesinot Behailu, editors. Proceedings of the 3rd Conference of the Ethiopian Society of Soil Science (ESSS). Addis Ababa, Ethiopia. Ethiopian Science and Technology Commission; 1996; p. 78-96.
73. Terefe H, Argaw M, Tamene L, Mekonnen K, Recha J, Solomon D. Effects of sustainable land management interventions on selected soil properties in Geda watershed, central highlands of Ethiopia. Ecol Process. 2020; 9(1): 1-11. https://doi.org/10.1186/s13 717-020-02 16- 2.
74. Jahn R, Blume HP, Asio VB, Spaargaren O, Schad P. Guidelines for soil description. 4th ed. Rome, FAO. 2006; 97 pp.
75. Gol C, Cakir M, Edis S, Yilmaz H. The effects of land-use/land-cover change and demographic processes (1950–2008) on soil properties in the Gokcay catchment, Turkey. Afr J Agric Res. 2010; 4:1670–1677. https://doi.org/10.5897/AJAR09.716.
76. Kokeb A, Muche M, Molla E. Assessing the Physical and Chemical Properties of Soil under Different Land-Use Types. J Environ Anal Toxicol. 2015; 5(5):309. https://doi.org/10.4172/2161-0525.1000309.
77. Selassie YG, Anemut F, Addisu S. The effects of land-use types, management practices and slope classes on selected soil physical and chemical properties in Zikre watershed, North-Western Ethiopia. Environ Syst Res. 2015; 4:3. https://doi.org/10.1186/s40068-015-0027-0.
78. Molla FE, Yalew AM. The effects of land-use types and soil depth on soil properties of Agedit watershed, Northwest. Ethiopia. Ethiop J Sci & Technol. 2018; 11(1): 39-56. https://doi.org/10.4314/ejst.v11i1.4.

79. Gniazdowski Z. New interpretation of principal components analysis. Zesz Nauk WWSI. 2017; 11:43-65. https://doi.org/10.26348/znwwsi.16.43.

80. Jolliffe IT, Cadima J. Principal component analysis: a review and recent developments, Philos Trans A Math Phys Eng Sci. 2016; 374(2065):20150202. Epub 2016/03/10. https://doi.org/10.1098/rsta.2015.0202.

81. Tubiello FN, Salvatore M, Condor Golec R, Rossi S, Ferrara A, Biancalani R et al.. The contribution of agriculture, forestry, and other land-use activities to global warming, 1990 –2012. Glob Change Biol. 2015; 21(7): 2655–2660. https://doi:10.1111/gcb.12865.

82. Galloway JN, Townsend AR, Erisman JW, Bekunda M, Cai Z, Freney JR et al.. Transformation of the nitrogen cycle: recent trends, questions, and potential solutions. Science. 2008; 320: 889–92. https://doi:10.1126/science.1136674.

83. Tian HQ, Lu CQ, Melillo J, Ren R, HuangY, Xu XF et al.. Food benefit and climate warming potential of nitrogen fertilizer uses in China. Environ Res Lett. 2012; 7. https://doi:10.1088/1748-9 326/7/4/044020.

84. Millar N, Robertso GP, Grace PR, Gehl RJ, Hoben JP. Nitrogen fertilizer management for nitrous oxide (N_2O) mitigation in intensive corn (Maize) production: an emissions reduction protocol for US Midwest agriculture. Mitig Adapt Strateg Glob Chang. 2010; 15(2):185-204. https://doi 10.1007/s11027-010-9212-7.

85. Food and Agriculture Organization (FAO). Integrated soil management for sustainable agriculture and food security in Southern and East Africa, FAO, Rome, Italy. 1999.

86. Novara A, Gristina L, Guaitoli F, Santoro A, Cerdà A. Managing soil nitrate with cover crops and buffer strips in Sicilian vineyards. Solid Earth. 2013; 4(2): 255–262. https://doi.org/10.5194/se-4-255-2013.

87. Venterea RT, Maharjan B, Dolan MS. Fertilizer source and tillage effects on yield- scaled nitrous oxide emissions in a corn cropping system. J. Environ. Qual. 2011; 40(5): 1521-1531. https://doi:10.2134/jeq2011.0039.

~~15.~~88. Snyder CS, Davidson EA, Smith P, Venterea RT. Agriculture: sustainable crop and animal production to help mitigate nitrous oxide emissions. Curr Opin Environ Sustain. 2014; 9-10: 46-54. https://doi.org/10.1016/j.cosust.2014.07.005.

Supporting information

S1 Table. Laboratory results of soil chemical properties in Fogera plain

S1 Fig. Factor loading plot: Organic carbon (OC), Total nitrogen (TN) Available phosphorus (avP), exchangeable calcium (ExchCa), Exchangeable potassium (ExchK), Cation exchange capacity (CEC), and Electrical conductivity (EC).

S2 Fig. Q-Q plots of soil fertility parameters in Fogera plain.
