# Supplementary material for: Spatial variability of soil chemical properties under different land-uses in Northwest Ethiopia
Source: PLoS One. 2021 Jun 23;16(6):e0253156. doi: 10.1371/journal.pone.0253156 (PMC8241222; doi:10.1371/journal.pone.0253156)
Supplement: Attachment — Submitted filename: Response to Reviewers.docx [file pone.0253156.s007.docx]

Date: May 23, 2021

**Rebuttal letter**

PONE-D-21-03433

We are glad about the academic editor and the reviewers’ comments, which strengthen the current version of the manuscript **“Spatial variability of soil properties under different land-uses in Northwest Ethiopia”.** In addition, our utmost sincere gratitude goes to you and the reviewers who devote their valuable time to bring our manuscript to a qualified paper.

We have provided a one by one reply to all concerns and comments given below. We thank you for your consideration of this resubmission and look forward to your response.

Best regards,

Gizachew Ayalew Tiruneh (on behalf of all co-authors)

Lecturer in Debre Tabor University

Ph.D. Fellow in soil science, Bahir Dar University

Email: tiruneh1972@gmail.com

**Editor’s comments**

Comments 1: A rebuttal letter that responds to each point raised by the academic editor and reviewer(s). You should upload this letter as a separate file labeled 'Response to Reviewers'.

Response: We addressed the concerns provided by the editor and reviewers and uploaded a file labeled “Response to Reviewers”.

Comments 2: A marked-up copy of your manuscript that highlights changes made to the original version. You should upload this as a separate file labeled 'Revised Manuscript with Track Changes'.

Response: We tried to do it.

Comments 3: An unmarked version of your revised paper without tracked changes. You should upload this as a separate file labeled 'Manuscript'.

Response: We have addressed accordingly.

Comments 4: If you would like to make changes to your financial disclosure, please include your updated statement in your cover letter.

Response: We have not made any changes to financial disclosure.

Comments 5: Guidelines for resubmitting your figure files are available below the reviewer comments at the end of this letter.

Response: We made corrections as per the guidelines

Comments 6: If applicable, we recommend that you deposit your laboratory protocols in [protocols.io](http://www.google.com/url?q=http%3A%2F%2Fprotocols.io&sa=D&sntz=1&usg=AFQjCNFk01OGntovdK1g5gZcchbvuKd9dA) to enhance the reproducibility of your results. Protocols.io assigns your protocol its own identifier (DOI) so that it can be cited independently in the future. For instructions see: [http://journals.plos.org/plosone/s/submission-guidelines#loc-laboratory-protocols](http://www.google.com/url?q=http%3A%2F%2Fjournals.plos.org%2Fplosone%2Fs%2Fsubmission-guidelines%23loc-laboratory-protocols&sa=D&sntz=1&usg=AFQjCNEu_U0-j5qqxwbP8TQOAcC7yBEtIA). Additionally, PLOS ONE offers an option for publishing peer-reviewed Lab Protocol articles, which describe protocols hosted on [protocols.io](http://www.google.com/url?q=http%3A%2F%2Fprotocols.io&sa=D&sntz=1&usg=AFQjCNFk01OGntovdK1g5gZcchbvuKd9dA). Read more information on sharing protocols at [https://plos.org/protocols?utm_medium=editorial-email&utm_source=authorletters&utm_campaign=protocols](https://www.google.com/url?q=https%3A%2F%2Fplos.org%2Fprotocols%3Futm_medium%3Deditorial-email%26utm_source%3Dauthorletters%26utm_campaign%3Dprotocols&sa=D&sntz=1&usg=AFQjCNHT2ELkBJUpUJ87fBMekTDtKfPoQw).

Response: We do not have our own publishable laboratory protocols

Comments 7: Please review your reference list to ensure that it is complete and correct. If you have cited papers that have been retracted, please include the rationale for doing so in the manuscript text, or remove these references and replace them with relevant current references. Any changes to the reference list should be mentioned in the rebuttal letter that accompanies your revised manuscript. If you need to cite a retracted article, indicate the article’s retracted status in the References list and also include a citation and full reference for the retraction notice.

Response: We have reviewed and checked that the references are complete and correct.

**Reviewers' comments**:

**Reviewer #1**:

Comments 1. If the authors have adequately addressed your comments raised in a previous round of review and you feel that this manuscript is now acceptable for publication, you may indicate that here to bypass the “Comments to the Author” section, enter your conflict of interest statement in the “Confidential to Editor” section, and submit your "Accept" recommendation.

Reviewer #1: All comments have been addressed

Reviewer #2: (No Response)

Response: Dear Reviewers, thank you so much for taking your valuable time to elevate the quality of our manuscript.

2. Is the manuscript technically sound, and do the data support the conclusions?
The manuscript must describe a technically sound piece of scientific research with data that supports the conclusions. Experiments must have been conducted rigorously, with appropriate controls, replication, and sample sizes. The conclusions must be drawn appropriately based on the data presented.

Reviewer #1: Yes

Reviewer #2: Partly

Response: Thank you. We have gone thoroughly the revised manuscript, and hopefully that the second Reviewer will be satisfied.

3. Has the statistical analysis been performed appropriately and rigorously?

Reviewer #1: Yes

Reviewer #2: Yes

Response: Thank you.

4. Have the authors made all data underlying the findings in their manuscript fully available?

The [PLOS Data policy](http://www.google.com/url?q=http%3A%2F%2Fwww.plosone.org%2Fstatic%2Fpolicies.action%23sharing&sa=D&sntz=1&usg=AFQjCNEJj_X58xRcqbJNp-2AK_B_FYZKGw) requires authors to make all data underlying the findings described in their manuscript fully available without restriction, with rare exception (please refer to the Data Availability Statement in the manuscript PDF file). The data should be provided as part of the manuscript or its supporting information, or deposited to a public repository. For example, in addition to summary statistics, the data points behind means, medians and variance measures should be available. If there are restrictions on publicly sharing data—e.g. participant privacy or use of data from a third party—those must be specified.

Reviewer #1: No

Reviewer #2: No

Response: We have described the data in the manuscript and attached as supporting information (S1 table and S2 table), and we do hope that the Reviewers’ concerns will be addressed.

5. Is the manuscript presented in an intelligible fashion and written in standard English?

PLOS ONE does not copyedit accepted manuscripts, so the language in submitted articles must be clear, correct, and unambiguous. Any typographical or grammatical errors should be corrected at revision, so please note any specific errors here.

Reviewer #1: Yes

Reviewer #2: No

Response: Thank you. We have thoroughly revised our manuscript with the help of Grammarly (premium) and Turnitin software, and we do hope that the second Reviewer’s concerns will be addressed.

6. Review Comments to the Author

Please use the space provided to explain your answers to the questions above. You may also include additional comments for the author, including concerns about dual publication, research ethics, or publication ethics. (Please upload your review as an attachment if it exceeds 20,000 characters)

Reviewer #1: Dear author(s),
Thank you very much for addressing my concerns.
It was a pleasure to work on your manuscript and provide some constructive suggestions/ comments.

Reviewer #2: It is true that the authors have tried to address some of the concerns raised in the previous version of the manuscript. However, the authors have failed to present their results in a clear and concise manner. Some of the sentences are not clear and many are laden with lots of grammatical errors. The authors are also advised to write their conclusion in such a manner that it represents the result of their study

Response: Thank you. We have revised the results and conclusions and we do hope that the second Reviewer’s concerns will be addressed. .

7. PLOS authors have the option to publish the peer review history of their article ([what does this mean?](https://www.google.com/url?q=https%3A%2F%2Fjournals.plos.org%2Fplosone%2Fs%2Feditorial-and-peer-review-process%23loc-peer-review-history&sa=D&sntz=1&usg=AFQjCNFLAUVWL2CBiof2AyELQ5heZ-N9BQ)). If published, this will include your full peer review and any attached files.

If you choose “no”, your identity will remain anonymous but your review may still be made public.

**Do you want your identity to be public for this peer review?** For information about this choice, including consent withdrawal, please see our [Privacy Policy](https://www.google.com/url?q=https%3A%2F%2Fwww.plos.org%2Fprivacy-policy&sa=D&sntz=1&usg=AFQjCNFbCnwIHtkhQjFNrWELvfbJHxmI2w).

Reviewer #1: No

Reviewer #2: No

Response: Yes

[NOTE: If reviewer comments were submitted as an attachment file, they will be attached to this email and accessible via the submission site. Please log into your account, locate the manuscript record, and check for the action link "View Attachments". If this link does not appear, there are no attachment files.]

While revising your submission, please upload your figure files to the Preflight Analysis and Conversion Engine (PACE) digital diagnostic tool, [https://pacev2.apexcovantage.com/](https://www.google.com/url?q=https%3A%2F%2Fpacev2.apexcovantage.com%2F&sa=D&sntz=1&usg=AFQjCNFEo2kwIg9_2n258L5WDvGur7KdjQ). PACE helps ensure that figures meet PLOS requirements. To use PACE, you must first register as a user. Registration is free. Then, login and navigate to the UPLOAD tab, where you will find detailed instructions on how to use the tool. If you encounter any issues or have any questions when using PACE, please email PLOS at [figures@plos.org](https://mail.google.com/mail/u/0/h/1vklefp1x8h9x/?&cs=wh&v=b&to=figures@plos.org). Please note that Supporting Information files do not need this step.

Response: Thank you. We have used PACE with this submission, so this should be right.

Please note that once again, thank you very much. Your comments are greatly appreciated.

Best regards,

Gizachew Ayalew Tiruneh (on behalf of all co-authors)

Lecturer in Debre Tabor University

Ph.D. Fellow in soil science, Bahir Dar University

Email: tiruneh1972@gmail.com
